# Subcutaneous trastuzumab with pertuzumab and docetaxel in HER2-positive metastatic breast cancer: Final analysis of MetaPHER, a phase IIIb single-arm safety study

**DOI:** 10.1007/s10549-021-06145-3

**Published:** 2021-03-21

**Authors:** Sherko Kuemmel, Carlo A. Tondini, Jacinta Abraham, Zbigniew Nowecki, Bartosz Itrych, Erika Hitre, Bogusława Karaszewska, Alejandro Juárez-Ramiro, Flavia Morales-Vásquez, Jose Manuel Pérez-García, Servando Cardona-Huerta, Estefania Monturus, Marco Sequi, Eleonora Restuccia, Mark Benyunes, Miguel Martín

**Affiliations:** 1 Breast Unit, Kliniken Essen-Mitte, Henricistrasse 92, 45136 Essen, Germany; 2 Clinic for Gynecology With Breast Center, Charité–Universitätsmedizin Berlin, Berlin, Germany; 3 Department of Onco-Hematology, ASST Papa Giovanni XXIII, Bergamo, Italy; 4 Department of Clinical Oncology, Velindre Cancer Centre, Cardiff, UK; 5 Klinika Nowotworów Piersi i Chirurgii Rekonstrukcyjnej, Centrum Onkologii-Instytut, Warsaw, Poland; 6 Department of Oncology, Magodent, Warsaw, Poland; 7 Department of Medical Oncology and Clinical Pharmacology “B”, National Institute of Oncology, Budapest, Hungary; 8 Przychodnia Lekarska KOMED, Konin, Poland; 9 Medical Oncology, CME Consultorio de Medicina Especializada, Mexico City, Mexico; 10 FUCAM, Instituto Nacional de Cancerología de Mexico, Mexico City, Mexico; 11 Medical Oncology Department, Vall D´Hebron Institute of Oncology (VHIO), Hospital Universitari Vall D’Hebron, Barcelona, Spain; 12 Centro de Cáncer de Mama, Hospital Zambrano-Hellion, Tecnológico de Monterrey, Monterrey, Mexico; 13 Product Development Oncology, F. Hoffmann-La Roche Ltd, Basel, Switzerland; 14 Biostatistics, F. Hoffmann-La Roche Ltd, Basel, Switzerland; 15 Present Address: Biostatistics, PAREXEL, Milan, Italy; 16 Product Development Oncology, Genentech, Inc., South San Francisco, CA USA; 17 Departamento de Medicina, Universidad Complutense de Madrid, Instituto de Investigación Sanitaria Gregorio Marañón, CIBERONC, Madrid, Spain; 18 Present Address: Klinika Onkologii i Chorób Piersi CMKP, Centralny Szpital Kliniczny MSWiA, Warsaw, Poland; 19 Present Address: International Breast Cancer Center (IBCC), Quiron Group, Barcelona, Spain

**Keywords:** HER2-positive breast cancer, Metastatic breast cancer, Pertuzumab, Route of administration, Safety, Subcutaneous trastuzumab

## Abstract

**Purpose:**

Intravenous trastuzumab, pertuzumab, and docetaxel are first-line standard of care for patients with HER2-positive metastatic breast cancer (mBC). MetaPHER is the first study assessing the safety and tolerability of subcutaneous trastuzumab plus intravenous pertuzumab and chemotherapy in a global patient population with HER2-positive mBC.

**Methods:**

In this open-label, single-arm, multicenter, phase 3b study, eligible patients were ≥ 18 years old with histologically/cytologically confirmed previously untreated HER2-positive mBC. All received ≥ 1 subcutaneous trastuzumab 600 mg fixed dose plus intravenous pertuzumab (loading dose: 840 mg/kg; maintenance: 420 mg/kg) and docetaxel (≥ 6 cycles; initial dose 75 mg/m^2^) every 3 weeks. The primary objective was safety and tolerability; secondary objectives included efficacy.

**Results:**

At clinical cutoff, 276 patients had completed the study; median duration of follow-up was 27 months. The most common any-grade adverse events were diarrhea, alopecia, and asthenia; the most common grade ≥ 3 events were neutropenia, febrile neutropenia, and hypertension. There were no cardiac deaths and mean left ventricular ejection fraction was stable over time. Median investigator-assessed progression-free survival was 18.7 months; objective response rate was 75.6%.

**Conclusions:**

Safety and efficacy with subcutaneous trastuzumab plus intravenous pertuzumab and docetaxel in mBC are consistent with historical evidence of intravenous trastuzumab with this combination. Findings further support subcutaneous administration not affecting safety/efficacy profiles of trastuzumab in HER2-positive BC with increased flexibility in patient care. A fixed-dose combination of pertuzumab and trastuzumab for subcutaneous injection has recently been approved for the treatment of HER2-positive early/mBC, further addressing the increasing relevance of and need for patient-centric treatment strategies.

**Trial registration:**

NCT02402712

**Supplementary Information:**

The online version contains supplementary material available at 10.1007/s10549-021-06145-3.

## Introduction

In previously untreated patients with human epidermal growth factor receptor 2 (HER2)-positive metastatic breast cancer (mBC), the pivotal phase 3 CLEOPATRA study demonstrated improved progression-free survival (PFS: 18.5 vs. 12.4 months, hazard ratio [HR] 0.62; 95% confidence interval, 0.51–0.75; *P* < 0.001), as assessed by an independent review facility, with first-line intravenous fixed-dose pertuzumab (P IV), weight-based intravenous trastuzumab (H IV), and docetaxel (D IV) compared with placebo, H IV, and D IV. Statistical significance for improved overall survival (OS) with P IV plus H IV and D IV was reached in a secondary interim analysis and further confirmed after an additional year (at 30-month follow-up: not reached vs. 37.6 months, HR 0.66; at 4 years’ follow-up: 56.5 vs. 40.8 months, HR 0.68) [1–3]. Based on these results, H IV plus P IV and D IV is the first-line standard of care for these patients [4]. The CLEOPATRA end-of-study analysis at 99-month follow-up (maximum 120 months) has continued to show the improved OS benefit of this regimen (57.1 vs. 40.8 months, HR 0.69) and confirmed the consistency of its long-term safety, including maintained cardiac safety, compared with placebo, H IV, and D IV [5].

Despite the benefit of H IV in HER2-positive mBC [6], the current IV formulation involves dose calculations, aseptic preparation of infusion fluids, long infusion durations (~ 30–90 min), and placement of a central line for administration [7, 8]. Subcutaneous trastuzumab (H SC) contains a fixed dose of 600 mg of H co-formulated with 2000 U/m of recombinant human hyaluronidase (rHuPH20), a permeant enhancer that allows absorption and dispersion of large fluid volumes through degradation of hyaluronan [9]. It can be administered in ~ 2–5 min and has been shown to reduce patient chair and active healthcare professional time, compared with H IV (20.9 vs. 77.8 min [*P* < 0.0001] and 5.1 vs. 20.8 min [*P* < 0.0001], respectively) [8, 10]. In contrast to H IV, a loading dose and weight-adjusted dose are not required for H SC. Phase 2 and 3 studies have also reported higher patient preference and healthcare professional satisfaction with H SC compared with H IV, in both HER2-positive early breast cancer (eBC) and mBC (PrefHer and MetaspHer, respectively) [11–13].

In the pivotal phase 3 HannaH study, H SC was non-inferior to H IV in patients with HER2-positive eBC, based on co-primary endpoints of pathologic complete response in the breast and serum trough concentration at pre-dose cycle 8 [14]. Event-free survival and OS, as well as safety, were also shown to be comparable between the two arms [14–17]. SafeHer further supported safety and tolerability of H SC as adjuvant therapy with concurrent or sequential chemotherapy for HER2-positive eBC; MetaspHer showed similar results in the metastatic setting [13, 18–20]. SAPPHIRE showed similar safety and tolerability of H SC compared with H IV plus P IV and a taxane in the metastatic setting; however, this was a study of only 50 patients and such results have not yet been demonstrated globally.

Here, we report results from the primary and final analysis of MetaPHER (NCT02402712). To our knowledge, this is the largest study to evaluate safety and tolerability of first-line H SC plus P IV and D IV in patients with HER2-positive mBC.

## Materials and methods

### Study design and patients

MetaPHER was an open-label, single-arm, multicenter, phase 3b study. Full details of the study design are provided in the trial protocol in the supplementary materials (Online Resource 1). Eligible patients were aged ≥ 18 years with histologically or cytologically confirmed HER2-positive mBC previously untreated with systemic non-hormonal anti-cancer therapy. Prior treatment with ≤ 2 lines of hormonal therapy, one of which could be in combination with everolimus, was permitted. Hormonal therapy concomitant with the use of P IV and H IV was permitted after chemotherapy discontinuation. Additional inclusion criteria included baseline left ventricular ejection fraction (LVEF) ≥ 50%. Key exclusion criteria were prior adjuvant/neoadjuvant treatment with any anti-HER2 agent other than H for BC, a disease-free interval of < 6 months from completion of adjuvant/neoadjuvant systemic non-hormonal treatment to recurrence of BC, and radiographic (computer tomography or magnetic resonance imaging) evidence of uncontrolled (symptomatic or requiring treatment with continuous corticosteroids) central nervous system metastases.

### Treatment

All patients received ≥ 1 dose of H SC (fixed-dose 600 mg) plus P IV (loading dose: 840 mg/kg; maintenance dose: 420 mg/kg) every 3 weeks. D IV was also administered every 3 weeks for ≥ 6 cycles with a recommended initial dose of 75 mg/m^2^; continuation after cycle 6 was at the discretion of the treating physician and patient. The dose of docetaxel could be escalated to 100 mg/m^2^ if well tolerated. Granulocyte colony-stimulating factor was used according to product license and approved prescribing information for docetaxel and American Society of Clinical Oncology clinical guidelines [21]. Treatment was continued until disease progression, unacceptable toxicity, withdrawal of consent, death, or predefined study end.

### Endpoints

The primary objective was evaluation of safety and tolerability. Adverse events (AEs) and cardiac AEs were graded according to the National Cancer Institute’s Common Terminology Criteria for Adverse Events (NCI-CTCAE) version 4.0 [22]. Heart failures were classified according to the New York Heart Association Functional Classification system.

Secondary objectives were evaluation of efficacy (investigator-assessed PFS, OS, and investigator-assessed objective response rate [ORR]) and incidence of anti-H and anti-rHuPH20 antibody formation. Investigator-assessed PFS and ORR were determined using Response Evaluation Criteria In Solid Tumors version 1.1.

### Statistics

The planned sample size was 400 patients. The primary objective was assessed at 24 months after enrollment of the last patient, with analyses performed in all patients who received ≥ 1 dose of any study drug. The Kaplan–Meier method was used to estimate the medians of PFS and OS. All results are descriptive.

## Results

### Patients and treatment exposure

**Fig. 1:**
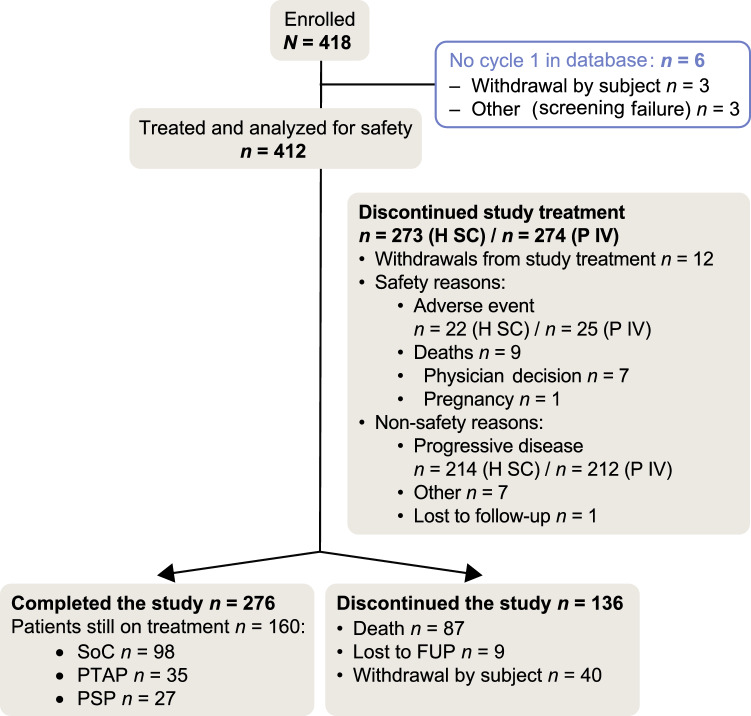
Patient dispositions. *H SC* subcutaneous trastuzumab, *FUP* follow-up period, *P IV* intravenous pertuzumab, *PSP* patient support program, *PTAP* post-trial access program, *SoC* standard of care

A total of 418 patients were enrolled in the study at 88 locations across 12 countries (May 6, 2015–February 23, 2017); 412 patients received ≥ 1 cycle of treatment and were analyzed for safety; median duration of follow-up was 27 months. At the date of clinical cutoff for final analysis (February 22, 2019), 276 patients had completed the study and 160 remained on treatment (Fig. 1).

The mean age of patients was 55.6 years (standard deviation: 11.7) (Online Resource 2). All patients enrolled had HER2-positive disease and most had visceral disease (*n* = 306 [74.3%]) and estrogen receptor- and/or progesterone receptor-positive hormonal status [*n* = 290 (70.4%)]. Approximately half of patients did not receive prior neoadjuvant or adjuvant treatment; 131 patients (31.8%) had received prior H therapy.

The median numbers of cycles for H SC, P IV, and D IV were 22.0, 21.5, and 6.0, respectively. The maximum number of cycles for H SC and P IV was 63; that of D IV was 18. Among the 195 patients who received ≥ 1 anti-cancer treatment after study treatment discontinuation and disease progression, 160 (82.1%) were treated with HER2-targeted therapies (Online Resource 3). From first cycle onwards, 100 patients (24.3%) were treated with prophylactic granulocyte colony-stimulating factor (253 treatments received).

### Safety

A safety overview is provided in Table 1. Any-grade and grade ≥ 3 AEs occurred in 406 (98.5%) and 221 (53.6%) patients, respectively. The most common any-grade AEs were diarrhea (*n* = 261 [63.3%]), alopecia (*n* = 193 [46.8%]), and asthenia (*n* = 137 [33.3%]) (Table 2). The most common grade ≥ 3 AEs were neutropenia (*n* = 52 [12.6%]), febrile neutropenia (*n* = 35 [8.5%]), and hypertension (*n* = 25 [6.1%]) (Table 2). Investigator-reported administration-related and local injection site reactions occurred in 87 patients (21.1%) (Table 2); H SC-related reactions occurred in 21 patients (5.1%), and all were grade 1. Serious AEs were reported in 107 patients (26.0%), treatment-related AEs in 399 patients (96.8%), AEs leading to withdrawal from any study treatment in 87 patients (21.1%; most frequently withdrawal of D IV [76/87; 87.4%]), and AEs leading to interruption of any study treatment in 147 patients (35.7%) (Table 1).

**Table 1:** Safety summary

	H SC + P IV + D IV (*N* = 412)
Any AE	406 (98.5)
Grade ≥ 3 AE	221 (53.6)
Serious AE	107 (26.0)
Death	87 (21.1)
Death due to disease progression	73 (17.7)
Death due to AEs	9 (2.2)
Death due to other causes	5 (1.2)
Related AE^a^	399 (96.8)
AE leading to drug withdrawal^a^	87 (21.1)
AE leading to drug interruption^a^	147 (35.7)
Cardiac AE	
Grade ≥ 3 cardiac AE^b^	3 (0.7)
Serious AE suggestive of CHF^c^	1 (0.2)
Cardiac death^d^	0

^a^Any event related to any study treatment component (H SC, P IV, or D IV)

^b^Events classified as System Organ Class “Cardiac Disorders”

^c^Serious events classified using the SMQ “Cardiac Failure”

^d^Deaths with SOC Cardiac Disorders as the primary cause

*AE* adverse event, *CHF* congestive heart failure, *D IV* intravenous docetaxel, *H SC* subcutaneous trastuzumab, *P IV* intravenous pertuzumab, *SMQ* Standardized MedDRA Query

Data are number of patients (%)

**Table 2:** Any-grade and grade ≥ 3 AEs and investigator-reported AEs

	H SC + P IV + D IV (*N* = 412)	
	Any grade	Grade ≥ 3
AE		
Leukopenia	29 (7.0)	15 (3.6)
Febrile neutropenia	35 (8.5)	35 (8.5)
Neutropenia	75 (18.2)	52 (12.6)
Diarrhea	261 (63.3)	21 (5.1)
Mucositis	68 (16.5)	3 (0.7)
Interstitial lung disease	5 (1.2)	1 (0.2)
Rash	68 (16.5)	4 (1.0)
Hypersensitivity, anaphylaxis	1 (0.2)	1 (0.2)
Investigator-reported AE		
ARR and local injection site reactions	87 (21.1)	5 (1.2)
ARR and local injection site reactions: Only H SC-related	21 (5.1)	0
ARR and local infusion site reactions: Only P IV infusion-related	22 (5.3)	1 (0.2)
ARR and local infusion site reactions: Only D IV infusion-related	48 (11.7)	4 (1.0)

*AE* adverse event, *ARR* administration-related reactions, *D IV* intravenous docetaxel, *H SC* subcutaneous trastuzumab, *P IV* intravenous pertuzumab

Data are number of patients (%)

There were 87 deaths (21.1%) (Table 1). Most common causes were disease progression (*n* = 73 [17.7%]), AEs (*n* = 9 [2.2%]), and other causes occurring after treatment discontinuation determined to have an “unknown” cause by the investigator (*n* = 5 [1.2%]). AEs leading to death were “unexplained death” (*n* = 4), aortic dissection, lactic acidosis, community-acquired pneumonia (without neutropenia), B-cell lymphoblastic leukemia, and suicide (*n* = 1 each).

Three patients (0.7%) had grade ≥ 3 cardiac AEs (Table 1), which were supraventricular tachycardia (*n* = 1) and left ventricular dysfunction (*n* = 2). Serious AEs suggestive of congestive heart failure occurred in one patient (0.2%) (Table 1) in the form of left ventricular dysfunction; there were no cardiac deaths (Table 1). Mean LVEF was stable over time (Online Resource 4), with decreases at cycles 60 and 63 and during safety follow-up at weeks 120 and 144; notably, small numbers of patients were assessed at these timepoints. Table 3 provides a summary of significant LVEF declines (reduction of ≥ 10% from baseline to LVEF < 50%). Median baseline LVEF was 64% and median post-baseline worst LVEF was 58%. Of the 396 patients with LVEF measurements at baseline and ≥ 1 post-baseline visit, 40 (10.1%) had a significant LVEF drop.

**Table 3:** Summary of significant LVEF declines, overall and by treatment phase

	H SC + P IV + D IV		
	Overall (*N* = 412)	Treatment phase (*n* = 387)	Post-treatment phase (*n* = 387)
Median baseline LVEF (range)	64 (50–83)^a^		
Median overall post-baseline worst value (range)	58 (30–74)^b^	59.0 (30–74)	60.0 (34–75)
Patients with baseline and ≥ 1 post-baseline value measured			
Increase or no change	89 (22.5)^c^	93 (24.2)^d^	65 (40.1)^e^
Decrease of < 10% points from baseline	182 (46.0)^c^	178 (46.2)^d^	61 (37.7)^e^
Decrease of ≥ 10% points from baseline	125 (31.6)^c^	114 (29.6)^d^	36 (22.2)^e^
Patients with LVEF < 50% and decrease ≥ 10% points from baseline	40 (10.1)^c^	37 (9.6)	13 (8.0)
LVEF 45%–50% and decreased ≥ 10% points from baseline	21 (5.3)^c^	21 (5.5)	4 (2.5)
LVEF < 45% and decreased ≥ 10% points from baseline	22 (5.6)^c^	19 (4.9)	9 (5.6)

^a^*n* = 411

^b^*n* = 398

^c^*n* = 396

^d^*n* = 385

^e^*n* = 162

*AE* adverse event, *D IV* intravenous docetaxel, *H SC* subcutaneous trastuzumab, *LVEF* left ventricular ejection fraction, *P IV* intravenous pertuzumab

Data are median (range) or number of patients (%)

Patients were also analyzed by hormone receptor status and treatment with hormonal therapy. The most common any-grade AEs for patients with hormone receptor-positive BC who received hormonal therapy were diarrhea (*n* = 83 [64.8%]), alopecia (*n* = 59 [46.1%]), and asthenia (*n* = 48 [37.5%]). The most common grade ≥ 3 events for the same subgroup were febrile neutropenia (*n* = 16 [12.5%]), neutropenia (*n* = 13 [10.2%]), and diarrhea (*n* = 9 [7.0%]). In both cases, this was found not to differ from patients with hormone receptor-positive BC who did not receive hormonal therapy. Patients with hormone receptor-positive BC that received ≥ 1 dose of H IV/P IV after D IV discontinuation showed a low incidence of grade ≥ 3 events that also did not differ depending on hormonal therapy.

### Efficacy

**Fig. 2:**
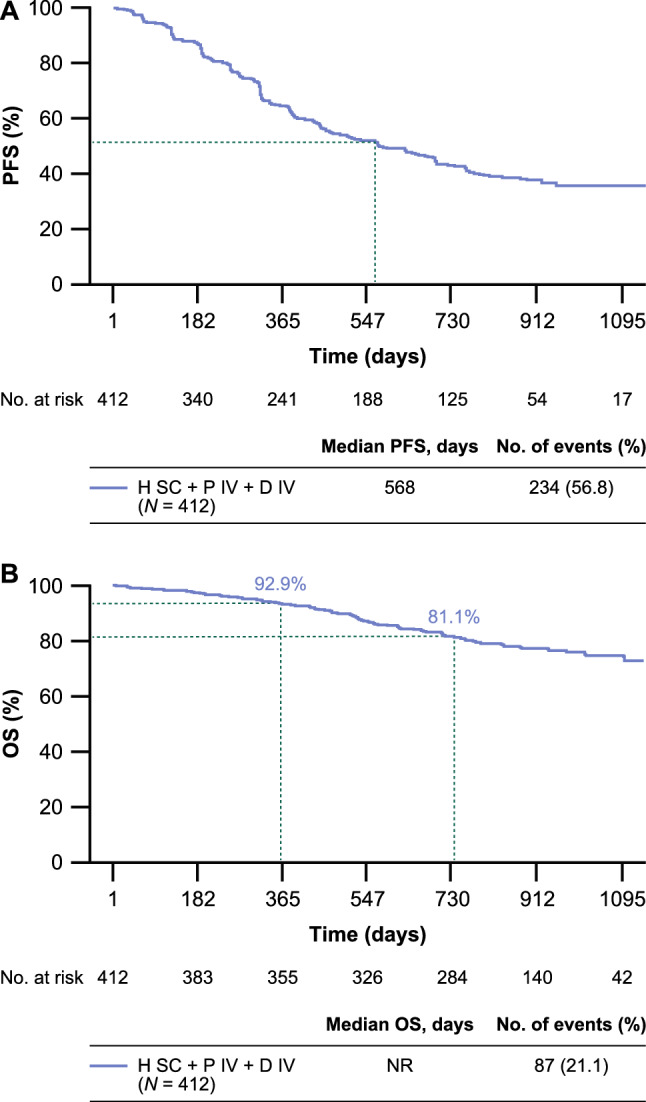
Investigator-assessed PFS and OS. **a**, investigator-assessed PFS. **b**, OS. *D IV* intravenous docetaxel, *H SC* subcutaneous trastuzumab, *NR* not reported, *OS* overall survival, *P IV* intravenous pertuzumab, *PFS* progression-free survival

Median investigator-assessed PFS was 18.7 months (234 events [56.8%]) (Fig. 2a). Median OS was not reached by study end (87 events [21.1%]), and OS rates at 12 and 24 months were 92.89% and 81.13%, respectively (Fig. 2b). ORR was 75.6%; 42 (12.5%) and 212 (63.1%) patients achieved a complete and partial response, respectively (Online Resource 5). The clinical benefit rate was 92.0% (309 patients). At 2 years, investigator-assessed PFS was greater for those given hormonal therapy compared with those that were not (55 events [53.8%] vs. 99 events [33.2%]).

### Anti-drug antibodies for H SC

Fifty-six (14.1%) and 95 (24.0%) patients were positive for anti-drug antibodies (ADAs) at baseline and post-baseline, respectively (Online Resource 6). Of the 95 patients with post-baseline ADAs, these were treatment-induced in 82 and treatment-enhanced in 13; 42/82 patients with treatment-induced ADAs had transient ADAs, while 40 had persistent ADAs. The median time to ADA onset was 3 weeks, and titers ranged from 1.00 to 512.00. Two patients (2.1%) had administration-related reactions (ARRs), both were H SC-related and occurred within 24 h of administration. Seventeen of the 300 patients who were ADA-negative post-baseline (5.7%) also had ARRs. In both cases, no patients experienced ARRs grade ≥ 3.

Anti-rHuPH20 antibodies post-baseline were observed in 11/396 patients.

## Discussion

In this primary and final analysis of 412 patients with HER2-positive mBC, the safety profile of first-line H SC plus P IV and D IV was tolerable and consistent with that of CLEOPATRA, in which H was delivered intravenously within the same combination regimen and in a similar patient population [1–3, 5]. No new safety signals were identified and AEs of particular interest to P + H therapy, including diarrhea, rash, mucosal inflammation, and febrile neutropenia, occurred less frequently in MetaPHER than in the CLEOPATRA secondary interim OS analysis [2]. The incidence of AEs was similar whether patients received additional hormone therapy or not, with diarrhea and febrile neutropenia the most common any-grade and grade ≥ 3 events, respectively.

Cardiac safety was further assessed in MetaPHER. Although grade ≥ 3 cardiac AEs and serious AEs suggestive of congestive heart failure were more frequent in the CLEOPATRA secondary analysis, no cardiac deaths were reported in either study [2]. Baseline and post-treatment median LVEFs were also similar. Although a higher proportion of patients had significant LVEF declines in MetaPHER, the majority of events were grade 1 or 2 and asymptomatic, and did not lead to study drug discontinuation.

Though efficacy results here were exploratory, investigator-assessed PFS and ORR findings support results observed with first-line H IV plus P IV and D IV in CLEOPATRA [2]. Median PFS was 18.7 months in MetaPHER and the CLEOPATRA secondary analysis [2]; ORRs were also similar, although the number of patients achieving complete response was slightly higher in CLEOPATRA vs. MetaPHER [1, 2].

The incidence of post-treatment ADAs to H SC (24%) was higher in MetaPHER than in HannaH (14.9%) [14]; pre-existing ADAs from previous H IV treatment at baseline or increased anti-framework antibodies from H + P may explain this. Analysis of safety by immunogenicity status indicated no noticeable association between the presence of treatment-emergent ADAs for H SC and increased frequency or severity of ARRs.

Despite MetaPHER and CLEOPATRA including similar numbers of de novo patients with no prior therapy for BC (*n* = 205 [49.8%] and *n* = 218 [54.2%], respectively), the proportion who received H treatment prior was higher in our study (*n* = 131 [31.8%] vs. *n* = 47 [11.7%]) [1]. This is reflective of the fact that when the CLEOPATRA study design was developed, use of H as adjuvant treatment for BC was not as common. Race/ethnic group also differed between CLEOPATRA and our study, with MetaPHER including fewer Asians (*n* = 2 [0.5%] vs. *n* = 125 [31.1%]) and more White individuals (*n* = 347 [84.2%] vs. *n* = 245 [60.9%]). These differences, as well as differences in study design/procedures (e.g., different versions of NCI-CTCAE for AE grading, and MetaPHER permitting concomitant use of hormonal therapy with study drug and excluding patients with disease-free interval of < 6 months vs. 12 months for CLEOPATRA), reflect differences in scope and timing of this study vs. CLEOPATRA [1].

The findings from the present study further add to the body of evidence indicating that SC administration does not affect the safety and efficacy profiles of H in HER2-positive BC. In particular, results from MetaPHER further confirm that a bridging approach based on pharmacokinetics is effective in the development of SC formulations of monoclonal antibodies. In specific circumstances, it may allow extrapolation of data across indications in a regimen-agnostic manner, which could save time and resources in the development of future SC formulations. In fact, the HannaH study first demonstrated that H SC had non-inferior drug exposure compared to H IV, as well as non-inferior efficacy and comparable safety, in the neoadjuvant setting and in combination with chemotherapy in patients with HER2-positive eBC [14–17]. Based on the known efficacy, safety, and pharmacokinetic similarities of H IV across eBC and mBC and the consistent role of HER2 overexpression in driving tumor growth across the HER2 spectrum, the consistent results from MetaPHER of H SC in mBC and in combination with a P-based regimen were not surprising.

The relevance of SC formulations for patients and physicians is increasing and opportunities for more flexible care outside of the traditional hospital setting are emerging. In that regard, H SC has demonstrated an acceptable safety profile both when administered in the hospital and at home [23], and a public/private partnership in Italy (HERHOME) has recently been initiated to deliver H SC therapy in a patients’ home [24–26]. A fixed-dose combination of P + H for SC injection (PH FDC SC) has been recently approved by the US Food and Drug Administration and European Medicines Agency for the treatment of patients with HER2-positive eBC and mBC [27, 28], based on the demonstration of non-inferior pre-dose cycle 8 P and H serum trough concentration and comparable efficacy and safety to the IV formulations in patients with HER2-positive eBC [29]. PH FDC SC provides increased flexibility in patient care than H SC as both H and P can be administered subcutaneously in minutes rather than hours. Similar benefits to H SC vs. H IV, including reduced patient clinic time, improved comfort during administration, and greater patient preference, were also reported with PH FDC SC [30].

## Conclusions

As MetaPHER was a single-arm study, no comparator arm is available for direct comparisons of H SC plus P IV and D IV with H IV plus P IV and D IV. However, safety and efficacy results from this large cohort of patients with HER2-positive mBC in our study are consistent with the results of H IV plus P IV and D IV in CLEOPATRA and further support the conclusion of the pivotal HannaH study for H SC in eBC [1–3, 5, 14]. Together, these results indicate that efficacy and safety of H given within standard regimens including P for HER2-positive eBC and mBC are not affected by administration route. Results in the present study also complement evidence of increased flexibility of H SC to patients with BC, shortening administration time, and reducing patient clinic time. This novel route of administration of H is the first step in taking patients with HER2-positive BC to the next level of treatment convenience, opening the door to homecare therapy options.

## Supplementary Information

Below is the link to the electronic supplementary material.

Supplementary file1 (pdf 1900 kb)

Supplementary file2 (pdf 152 kb)

Supplementary file3 (pdf 139 kb)

Supplementary file4 (pdf 155 kb)

Supplementary file5 (pdf 139 kb)

Supplementary file6 (pdf 146 kb)

## Data Availability

Qualified researchers may request access to individual patient level data through the clinical study data request platform (https://vivli.org/). Further details on Roche's criteria for eligible studies are available here: https://vivli.org/members/ourmembers/. For further details on Roche's Global Policy on the Sharing of Clinical Information and how to request access to related clinical study documents, see here: https://www.roche.com/research_and_development/who_we_are_how_we_work/clinical_trials/our_commitment_to_data_sharing.htm

## Abbreviations

ADA: Anti-drug antibody
AE: Adverse event
ARR: Administration-related reaction
D IV: Intravenous docetaxel
eBC: Early breast cancer
H IV: Intravenous trastuzumab
H SC: Subcutaneous trastuzumab
HER2: Human epidermal growth factor receptor 2
HR: Hazard ratio
LVEF: Left ventricular ejection fraction
mBC: Metastatic breast cancer
NCI-CTCAE: National Cancer Institute’s Common Terminology Criteria for Adverse Events
ORR: Objective response rate
OS: Overall survival
P IV: Intravenous pertuzumab
PFS: Progression-free survival
PH FDC SC: Fixed-dose combination of pertuzumab and trastuzumab for subcutaneous injection
rHuPH20: Recombinant human hyaluronidase
