# Supplementary material for: Subcutaneous trastuzumab with pertuzumab and docetaxel in HER2-positive metastatic breast cancer: Final analysis of MetaPHER, a phase IIIb single-arm safety study
Source: Breast Cancer Res Treat. 2021 Mar 21;187(2):467–76. doi: 10.1007/s10549-021-06145-3 (PMC8189949; doi:10.1007/s10549-021-06145-3)
Supplement: Supplementary file 1 — Supplementary file1 (pdf 1900 kb) [file 10549_2021_6145_MOESM1_ESM.pdf]

Article type: Original Research Article

Title: Subcutaneous trastuzumab with pertuzumab and docetaxel in HER2-positive metastatic breast cancer: Final analysis of MetaPHER, a phase IIIb single-arm safety study

Authors: Sherko Kuemmel[1,2]*, Carlo A. Tondini[3], Jacinta Abraham[4], Zbigniew Nowecki[5], Bartosz Itrych[6,†], Erika Hitre[7], Bogusława Karaszewska[8], Alejandro Juárez-Ramiro[9], Flavia Morales-Vásquez[10], Jose Manuel Pérez García[11‡], Servando Cardona-Huerta[12], Estefania Monturus[13], Marco Sequi[14,15§], Eleonora Restuccia[13], Mark Benyunes[16] and Miguel Martín[17]

Author affiliations:

[1] Breast Unit, Kliniken Essen-Mitte, Essen, Germany

[2] Clinic for Gynecology with Breast Center, Charité – Universitätsmedizin Berlin, Berlin, Germany

[3] Department of Onco-Hematology, ASST Papa Giovanni XXIII, Bergamo, Italy

[4] Department of Clinical Oncology, Velindre Cancer Centre, Cardiff, UK

[5] Klinika Nowotworów Piersi i Chirurgii Rekonstrukcyjnej, Centrum Onkologii-Instytut, Warsaw, Poland

[6] Department of Oncology, Magodent, Warsaw, Poland

[7] Department of Medical Oncology and Clinical Pharmacology “B”, National Institute of Oncology, Budapest, Hungary

[8] Przychodnia Lekarska KOMED, Konin, Poland

[9] Medical Oncology, CME Consultorio de Medicina Especializada, Mexico City, Mexico

[10] FUCAM, Instituto Nacional de Cancerología de Mexico, Mexico City, Mexico

[11] Medical Oncology Department, Vall d´Hebron Institute of Oncology (VHIO), Hospital Universitari Vall d'Hebron, Barcelona, Spain

[12] Centro de Cáncer de Mama, Hospital Zambrano-Hellion, Tecnológico de Monterrey, Monterrey, Mexico

[13] Global Product Development, F. Hoffmann-La Roche Ltd, Basel, Switzerland

[14] Biostatistics, F. Hoffmann-La Roche Ltd, Basel, Switzerland

[15] Biostatistics, PAREXEL, Milan, Italy

[16] Global Product Development, Genentech, Inc., South San Francisco, CA, USA

[17] Instituto de Investigación Sanitaria Gregorio Marañón, CIBERONC, Departamento de Medicina, Universidad Complutense de Madrid, Madrid, Spain

*** Corresponding author:** Sherko Kuemmel, MD, PhD

Address: Breast Unit, Kliniken Essen-Mitte, Henricistrasse 92, 45136 Essen, Germany

Telephone: +49-201-174-33001

Email: s.kuemmel@kem-med.com

[†] **Current address:** Department of Oncology and Hematology, Central Clinical Hospital MSWiA, Warsaw, Poland (Itrych)

[‡] **Current address:** International Breast Cancer Center (IBCC), Quiron Group, Barcelona, Spain (Pérez García)

[§] Current address: CROS Academy, Bologna, Italy (Sequi)

Target journal: *Breast Cancer Research and Treatment*

PROTOCOL

**TITLE:**	**A MULTICENTER, OPEN-LABEL, SINGLE-ARM SAFETY STUDY OF HERCEPTIN® SC IN COMBINATION WITH PERJETA® AND DOCETAXEL IN TREATMENT OF PATIENTS WITH HER2–POSITIVE ADVANCED BREAST CANCER (METASTATIC OR LOCALLY RECURRENT)**
**PROTOCOL NUMBER:**	BO29159
**VERSION NUMBER:**	2
**EUDRACT NUMBER:**	2014-001458-40
**TEST PRODUCTS:**	Herceptin® for subcutaneous administration (RO0452317)<br>Perjeta® (RO4368451)
**MEDICAL MONITORS:**	[redacted], Pharm.D.<br>[redacted], M.D.
**SPONSOR:**	F. Hoffmann-La Roche Ltd
**DATE FINAL:**	20 October 2014
**DATE AMENDED:**	Version 2: See electronic date stamp below.

PROTOCOL AMENDMENT APPROVAL

Approver's Name	Title	Date and Time (UTC)
[redacted]	Company Signatory	12-Mar-2015 04:48:36

CONFIDENTIAL

This clinical study is being sponsored globally by F. Hoffmann-La Roche Ltd of Basel, Switzerland. However, it may be implemented in individual countries by Roche's local affiliates, including Genentech, Inc. in the United States. The information contained in this document, especially any unpublished data, is the property of F. Hoffmann-La Roche Ltd (or under its control) and therefore is provided to you in confidence as an investigator, potential investigator, or consultant, for review by you, your staff, and an applicable Ethics Committee or Institutional Review Board. It is understood that this information will not be disclosed to others without written authorization from Roche except to the extent necessary to obtain informed consent from persons to whom the drug may be administered.

"Use of this document is governed by the terms of use on the first page of this document."

PROTOCOL AMENDMENT, VERSION 2: RATIONALE

Protocol BO29159 has been amended to correct a typographical error in one of exclusion criteria from “at least” to “less than.”

No additional changes have been made. Substantive new information appears in italics. This amendment represents cumulative changes to the original protocol.

"Use of this document is governed by the terms of use on the first page of this document."

PROTOCOL AMENDMENT, VERSION 2:
SUMMARY OF CHANGES

PROTOCOL SYNOPSIS

The protocol synopsis has been updated to reflect the changes to the protocol, where applicable.

SECTION 4.1.2: Exclusion Criteria

Patients who meet any of the following criteria will be excluded from study entry. Assessments must be performed according to the timing specified in the Schedule of Assessments (see Appendix 1):

2. Disease-free interval of ~~at least~~ *less than* 6 months from completion of adjuvant or neoadjuvant systemic non-hormonal treatment to recurrence of breast cancer

"Use of this document is governed by the terms of use on the first page of this document."

TABLE OF CONTENTS

PROTOCOL AMENDMENT ACCEPTANCE FORM 10

PROTOCOL SYNOPSIS 11

1. BACKGROUND 21

1.1 Background on Breast Cancer 21

1.2 Background on Study Treatments 21

1.2.1 Human Epidermal Growth Factor Receptors 21

1.2.1.1 Herceptin® (Trastuzumab) for Subcutaneous Administration 22

1.2.1.2 Recombinant Human Hyaluronidase (rHuPH20) 22

1.2.1.3 Clinical Studies with Herceptin for Subcutaneous Administration 23

1.2.1.4 Perjeta® (Pertuzumab) 24

1.2.2 Docetaxel 29

1.3 Study Rationale and Benefit-Risk Assessment 30

2. OBJECTIVES 30

2.1 Primary Objective 30

2.2 Secondary Objectives 31

3. STUDY DESIGN 31

3.1 Description of Study 31

3.1.1 Internal Monitoring Committee 34

3.2 End of Study 34

3.3 Rationale for Study Design 34

3.4 Outcome Measures 35

3.4.1 Efficacy Outcome Measures 35

3.4.2 Safety Outcome Measures 35

4. MATERIALS AND METHODS 36

4.1 Patients 36

4.1.1 Inclusion Criteria 36

4.1.2 Exclusion Criteria 37

4.2 Method of Treatment Assignment and Blinding 39

"Use of this document is governed by the terms of use on the first page of this document."

4.3 Study Treatment ... 39
4.3.1 Formulation, Packaging, and Handling ... 39
4.3.1.1 Subcutaneous Herceptin ... 39
4.3.1.2 Intravenous Perjeta ... 39
4.3.1.3 Intravenous Docetaxel ... 40
4.3.2 Dosage, Administration, and Compliance ... 40
4.3.2.1 Subcutaneous Herceptin ... 40
4.3.2.2 Intravenous Perjeta ... 41
4.3.2.3 Intravenous Docetaxel ... 41
4.3.3 Investigational Medicinal Product Accountability ... 43
4.3.4 Post-Trial Access to Subcutaneous Herceptin ... 43
4.4 Concomitant Therapy ... 44
4.4.1 Permitted Therapy ... 44
4.4.2 Prohibited Therapy ... 45
4.5 Study Assessments ... 46
4.5.1 Informed Consent Form and Screening Log ... 46
4.5.2 Medical History and Demographic Data ... 46
4.5.3 Physical Examination and Vital Signs ... 47
4.5.4 Performance Status ... 47
4.5.5 Tumor and Response Evaluation ... 47
4.5.6 Cardiac Assessments ... 50
4.5.7 Laboratory Assessments ... 51
4.5.8 Herceptin Serum Concentration Assessments ... 52
4.6 Patient, Study, and Site Discontinuation ... 53
4.6.1 Patient Discontinuation ... 53
4.6.2 Study Treatment Discontinuation ... 53
4.6.3 Study and Site Discontinuation ... 54

5. ASSESSMENT OF SAFETY ... 54
5.1 Safety Plan ... 54
5.1.1 Toxicity Management Guidelines ... 54
5.1.1.1 Cardiac Safety ... 54

"Use of this document is governed by the terms of use on the first page of this document."

5.1.1.2 Administration-Related Reactions, Hypersensitivity Reactions, and Local-Site Reactions 57
5.1.1.3 Incomplete Dose or Dose Delay 58
5.1.1.4 Docetaxel Dose Modification for Toxicity 59
5.1.2 Warning and Precautions for Herceptin SC 61
5.1.2.1 Administration-Related Reactions, Allergic-Like Reactions, and Hypersensitivity 61
5.1.2.2 Pulmonary Events 62
5.1.2.3 Cardiac Dysfunction 62
5.1.3 Warning and Precautions for Perjeta 63
5.1.3.1 Risk of Hypersensitivity Reactions, Including Anaphylaxis and Administration-Related Symptoms 63
5.1.3.2 Risk of Cardiac Dysfunction 63
5.1.3.3 Risk of EGFR-Related Toxicities 64
5.1.4 Pregnancy 65
5.2 Safety Parameters and Definitions 66
5.2.1 Adverse Events 66
5.2.2 Serious Adverse Events (Immediately Reportable to the Sponsor) 67
5.2.3 Non-Serious Adverse Events (Immediately Reportable to the Sponsor) 68
5.3 Methods and Timing for Capturing and Assessing Safety Parameters 68
5.3.1 Adverse Event Reporting Period 69
5.3.2 Eliciting Adverse Event Information 70
5.3.3 Assessment of Severity of Adverse Events 70
5.3.4 Assessment of Causality of Adverse Events 71
5.3.5 Procedures for Recording Adverse Events 71
5.3.5.1 Administration-Related Reactions and Local Injection-Site Reactions 71
5.3.5.2 Diagnosis versus Signs and Symptoms 71
5.3.5.3 Adverse Events Occurring Secondary to Other Events 72
5.3.5.4 Persistent or Recurrent Adverse Events 72

"Use of this document is governed by the terms of use on the first page of this document."

5.3.5.5 Abnormal Laboratory Values ... 73
5.3.5.6 Abnormal Vital Sign Values ... 73
5.3.5.7 Abnormal Liver Function Tests ... 74
5.3.5.8 Deaths ... 74
5.3.5.9 Preexisting Medical Conditions ... 75
5.3.5.10 Lack of Efficacy or Worsening of Breast Cancer ... 75
5.3.5.11 Hospitalization or Prolonged Hospitalization ... 75
5.3.5.12 Adverse Events Associated with an Overdose or Error in Drug Administration ... 76
5.3.5.13 Adverse Events in Individuals Not Enrolled in the Study ... 76
5.4 Immediate Reporting Requirements from Investigator to Sponsor ... 76
5.4.1 Emergency Medical Contacts ... 77
5.4.2 Reporting Requirements for Serious Adverse Events and Non-Serious Adverse Events that are Immediately Reportable ... 77
5.4.3 Reporting Requirements for Pregnancies ... 77
5.4.3.1 Pregnancies in Female Patients ... 77
5.4.3.2 Abortions ... 78
5.4.3.3 Congenital Anomalies/Birth Defects ... 78
5.5 Follow-Up of Patients with Ongoing Adverse Events ... 78
5.5.1 Investigator Follow-Up ... 78
5.5.2 Sponsor Follow-Up ... 79
5.6 Post-Study Adverse Events ... 79
5.7 Expedited Reporting to Health Authorities, Investigators, Institutional Review Boards, and Ethics Committees ... 79
5.8 Review of Safety by an Internal Monitoring Committee ... 79
6. STATISTICAL CONSIDERATIONS AND ANALYSIS PLAN ... 80
6.1 Determination of Sample Size ... 80
6.2 Summaries of Conduct of Study ... 80
6.3 Summaries of Treatment Group Comparability ... 81

"Use of this document is governed by the terms of use on the first page of this document."

6.4 Efficacy Analyses 81
6.5 Safety Analyses 81
6.6 Immunogenicity Analyses 83
6.7 Interim Analyses 83

7. DATA COLLECTION AND MANAGEMENT 83
7.1 Data Quality Assurance 83
7.2 Electronic Case Report Forms 84
7.3 Source Data Documentation 84
7.4 Use of Computerized Systems 85
7.5 Retention of Records 85

8. ETHICAL CONSIDERATIONS 85
8.1 Compliance with Laws and Regulations 85
8.2 Informed Consent 86
8.3 Independent Ethics Committee 86
8.4 Confidentiality 87
8.5 Financial Disclosure 87

9. STUDY DOCUMENTATION, MONITORING, AND ADMINISTRATION 87
9.1 Study Documentation 87
9.2 Protocol Deviations 88
9.3 Site Inspections 88
9.4 Administrative Structure 88
9.5 Publication of Data and Protection of Trade Secrets 88
9.6 Protocol Amendments 89

10. REFERENCES 90

LIST OF TABLES

Table 1 Summary of Study Treatment Dose and Schedule 42
Table 2 Docetaxel Dose Adjustments 60
Table 3 Adverse Event Severity Grading Scale 70

"Use of this document is governed by the terms of use on the first page of this document."

LIST OF FIGURES

Figure 1 Independently Assessed Progression-Free Survival in Study TOC4129g/WO20698 27
Figure 2 Final Overall Survival Analysis 29
Figure 3 Study Design 33
Figure 4 Asymptomatic Decline in Left Ventricular Ejection Fraction: Algorithm for Continuation and Discontinuation of Perjeta and Herceptin Based on Left Ventricular Ejection Fraction Assessments 56

LIST OF APPENDICES

Appendix 1 Schedule of Assessments 93
Appendix 2 ICH Guidelines for Clinical Safety Data Management, Definitions, and Standards for Expedited Reporting, Topic E2a 97
Appendix 3 Eastern Cooperative Oncology Group Performance Status 99
Appendix 4 New York Heart Association Classification and Left Ventricular Systolic Dysfunction National Cancer Institute Common Terminology Criteria for Adverse Events Version 4.0 Grading 100
Appendix 5 Tumor Assessments (Response Evaluation Criteria in Solid Tumors) Version 1.1 102

"Use of this document is governed by the terms of use on the first page of this document."

PROTOCOL AMENDMENT ACCEPTANCE FORM

**TITLE:**	**A MULTICENTER, OPEN-LABEL, SINGLE-ARM SAFETY STUDY OF HERCEPTIN® SC IN COMBINATION WITH PERJETA® AND DOCETAXEL IN TREATMENT OF PATIENTS WITH HER2–POSITIVE ADVANCED BREAST CANCER (METASTATIC OR LOCALLY RECURRENT)**
**PROTOCOL NUMBER:**	BO29159
**VERSION NUMBER:**	2
**EUDRACT NUMBER:**	2014-001458-40
**TEST PRODUCTS:**	Herceptin® for subcutaneous administration (RO0452317)<br>Perjeta® (RO4368451)
**MEDICAL MONITORS:**	[redacted], Pharm.D.<br>[redacted], M.D.
**SPONSOR:**	F. Hoffmann-La Roche Ltd

I agree to conduct the study in accordance with the current protocol.

____

Principal Investigator's Name (print)

____ ____________________

Principal Investigator's Signature Date

Please return the signed original copy of the form as instructed by your local study monitor. Please retain a copy for your study files.

"Use of this document is governed by the terms of use on the first page of this document."

PROTOCOL SYNOPSIS

**TITLE:**	**A MULTICENTER, OPEN-LABEL, SINGLE-ARM SAFETY STUDY OF HERCEPTIN® SC IN COMBINATION WITH PERJETA® AND DOCETAXEL IN TREATMENT OF PATIENTS WITH HER2–POSITIVE ADVANCED BREAST CANCER (METASTATIC OR LOCALLY RECURRENT)**
**PROTOCOL NUMBER:**	BO29159
**VERSION NUMBER:**	2
**EUDRACT NUMBER:**	2014-001458-40
**TEST PRODUCT:**	Herceptin® for subcutaneous administration (RO0452317)<br>Perjeta® (RO4368451)
**PHASE:**	IIIb
**INDICATION:**	Advanced breast cancer (metastatic or locally recurrent)
**SPONSOR:**	F. Hoffmann-La Roche Ltd

Objectives

Primary Objectives

Primary Safety Objective

The primary objective for this study is to evaluate the safety and tolerability of Herceptin subcutaneous (SC) in combination with Perjeta intravenous (IV) plus docetaxel in patients with human epidermal growth factor 2 (HER2)–positive advanced (metastatic or locally recurrent) breast cancer.

- Overall safety profile as determined by adverse events of any grade of severity, and adverse events Grade ≥ 3 according to National Cancer Institute Common Terminology Criteria for Adverse Events (NCI CTCAE), Version 4.0) and cardiac function will be assessed including the following: cardiac events Grade ≥ 3, congestive heart failure (CHF), and cardiac death (see protocol)

Secondary Objectives

The secondary objectives for this study are to evaluate Herceptin SC in combination with Perjeta IV plus docetaxel with respect to:

- Efficacy parameters (see protocol)
  - Progression-free survival (PFS)
  - Overall Survival (OS)
  - Objective response rate (ORR)
- Incidence of anti-Herceptin and anti-rHuPH20 antibody formation

Study Design

Description of Study

This is an open-label, single-arm, multicenter, Phase IIIb study to evaluate the safety and tolerability of Herceptin SC in combination with Perjeta IV plus docetaxel (see figure below). Patients with HER2–positive advanced breast cancer (metastatic or locally recurrent) who have not previously received systemic non–hormonal anti-cancer therapy in the metastatic setting are

"Use of this document is governed by the terms of use on the first page of this document."

eligible to participate in the study. Enrollment is defined as first dose of study drug administration.

Four hundred patients are planned to be enrolled into the study at approximately 110 centers worldwide. Details of the study treatment are given in protocol.

Treatment Period

Every 3 weeks (21 days) the patient will receive Herceptin SC (fixed dose of 600 mg/5 mL), Perjeta IV (loading dose of 840 mg followed by 420 mg on Day 1 of each subsequent cycle), and docetaxel IV (at least six cycles with recommended initial dose of 75 mg/m^2). After Cycle 6, continuation of docetaxel treatment is at the discretion of the treating physician in agreement with the patient. The dose of docetaxel may be escalated to 100 mg/m^2 at the investigator's discretion on subsequent cycles if the initial dose is well tolerated.

End of Treatment

End of treatment for each patient is defined as receiving study medication until disease progression, unacceptable toxicity, withdrawal of consent, death, or predefined study end, whichever occurs first.

Post-Treatment Follow-up

If the patient discontinues all study treatments because of unacceptable toxicity, she will enter in follow-up and will have tumor assessments every 9 weeks (±3 days) until disease progression or predefined end of the study (see protocol), whichever occurs first.

In addition, patients with disease progression will continue to be followed until death, loss to follow-up, withdrawal of consent, predefined study end (see protocol), or study termination by Roche.

Figure: Study Design

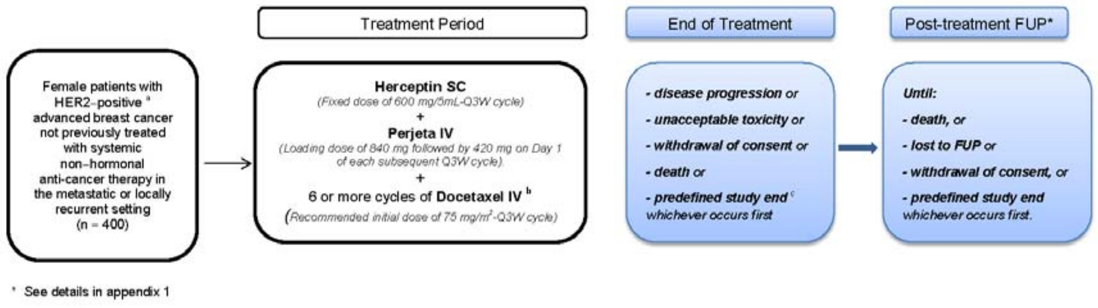

FUP = follow-up; HER2 = human epidermal growth factor receptor 2; IV = intravenous; Q3W = every 3 weeks; SC = subcutaneous.

See protocol for dose administration guidelines.

[a] Defined as either immunohistochemistry 3 + or in situ hybridization positive.

[b] After Cycle 6, continuation of docetaxel treatment is at the discretion of the treating physician in agreement with the patient. The dose of docetaxel may be escalated to 100 mg/m^2 at the investigator's discretion on subsequent cycles if the initial dose is well tolerated.

[c] Cutoff of Final Safety Analysis will occur 24 months after the last patients is recruited (see protocol).

Number of Patients

The proposed sample size to be enrolled in this study is 400 patients with the following rationale:

- The width of the 95% Pearson Clopper CI of the incidence of Grade ≥3 adverse events is reasonably small (71.8%, 80.3%) with 400 treated patients based on the observed incidence of Grade ≥3 adverse events of 76.2% for patients who were treated with Perjeta in combination with Herceptin IV in the TOC4129g/WO20698 study.

"Use of this document is governed by the terms of use on the first page of this document."

- Furthermore, with 400 treated patients on the observed incidence of cardiac events Grade ≥ 3 of 1.7% reported in the TOC4129g/WO20698 study, the width of the 95% Pearson Clopper CI of the incidence of Grade ≥ 3 cardiac adverse events is reasonably small (0.7%, 3.6%).

Target Population

Patients must meet the following criteria for study entry according to the timing specified in the Schedule of Assessments (see protocol):

1. Signed, written informed consent approved by the relevant Institutional Review Board (IRB) or Independent Ethics Committee (IEC)
2. Female patients aged 18 years or older
3. Histologically or cytologically confirmed and documented adenocarcinoma of the breast with metastatic or locally recurrent disease not amenable to curative resection. Patients with measurable and/or non-measurable disease evaluable according to Response Evaluation Criteria in Solid Tumors (RECIST), Version 1.1 (see protocol) are eligible.

   Notes:

   - Patients with only bone metastases are eligible provided that they have some bone metastases that have not been previously irradiated and tumor tissue samples from the primary tumor are available for local HER2 testing.
   - Locally recurrent disease must not be amenable to resection with curative intent.
   - Patients with de novo Stage IV disease are eligible.
4. HER2-positive disease (defined as either immunohistochemistry [IHC] 3+ or in situ hybridization [ISH] positive) as assessed by local laboratory on primary tumor or metastatic site if primary tumor not available (positive ISH is defined as a ratio of ≥ 2.0 for the number of HER2 gene copies to the number of signals for CEP17, or for single probe tests, a HER2 gene count > 4).
5. Eastern Cooperative Oncology Group (ECOG) Performance Status 0 or 1 (see protocol)
6. Left ventricular ejection fraction (LVEF) of at least 50%
7. Negative serum pregnancy test result in women of childbearing potential (WOCBP; defined as premenopausal or < 12 months of amenorrhea postmenopause and who have not undergone surgical sterilization)
8. WOCBP must agree to use a highly effective, non-hormonal form of contraception (such as surgical sterilization) or <u>two effective</u> forms of non-hormonal contraception or true abstinence during the treatment period and for at least 7 months after discontinuation of study treatment (see protocol for details).
9. Life expectancy of at least 12 weeks

Patients who meet any of the following criteria will be excluded from study entry. Assessments must be performed according to the timing specified in the Schedule of Assessments (see protocol):

1. Previous systemic non-hormonal anti-cancer therapy for the metastatic or locally recurrent disease. Note: Prior to study entry, up to two lines of hormonal therapy for metastatic or locally recurrent disease are permitted, one of which may be in combination with everolimus.
2. Disease-free interval of *less than* 6 months from completion of adjuvant or neoadjuvant systemic non-hormonal treatment to recurrence of breast cancer
3. Previous approved or investigative anti-HER2 agents as neoadjuvant or adjuvant therapy for any breast cancer treatment, except Herceptin
4. History of persistent Grade 2 or higher hematological toxicity resulting from previous adjuvant or neoadjuvant therapy

"Use of this document is governed by the terms of use on the first page of this document."

5. Patients with radiographic evidence of CNS metastases as assessed by computed tomography or magnetic resonance imaging (MRI) that are not well controlled (i.e., are symptomatic or require control with continuous corticosteroid therapy [e.g., dexamethasone]). Note: Patients with CNS metastases are permitted to participate in the study if they have been stable in the 3 months prior to screening (as assessed by the investigator) after receiving local therapy (irradiation, surgery, etc.) but have not received anti-HER2 therapy.
6. Current peripheral neuropathy of Grade 3 or greater
7. History of other malignancy within the last 5 years prior to first dose of study drug administration (dosing), except for carcinoma in situ of the cervix or basal cell carcinoma
8. Inadequate organ function, evidenced by the following laboratory results:
    a) ANC $<$ 1500 cells/mm^3
    b) Platelet count $<$ 100,000 cells/mm^3
    c) Hemoglobin $<$ 9 g/dL
    d) Total bilirubin greater than the upper limit of normal (ULN; unless the patient has documented Gilbert's syndrome)
    e) AST (SGOT) or ALT (SGPT) $>$ 2.5 $\times$ ULN
    f) AST (SGOT) or ALT (SGPT) $>$ 1.5 $\times$ ULN with concurrent serum alkaline phosphatase $>$ 2.5 $\times$ ULN. Serum alkaline phosphatase may be $>$ 2.5 $\times$ ULN only if bone metastases are present and AST (SGOT) and ALT (SGPT) are $<$ 1.5 $\times$ ULN
    g) Serum creatinine $>$ 2.0 mg/dL or 177 μmol/L
    h) INR and aPTT or PTT $>$ 1.5 $\times$ ULN (unless on therapeutic anticoagulation)
9. Uncontrolled hypertension (systolic blood pressure $>$ 150 mmHg and/or diastolic blood pressure $>$ 100 mmHg) with or without medication
10. Clinically significant cardiovascular disease as follows:
    a) Cerebrovascular accident/stroke or myocardial infarction within 6 months prior to first study medication, or
    b) Unstable angina, or
    c) History of or active CHF of any NYHA criteria, or
    d) History of or ongoing serious cardiac arrhythmia requiring medication (except controlled atrial fibrillation or paroxysmal supraventricular tachycardia)
11. History of LVEF decline to below 50% during or after prior Herceptin neoadjuvant or adjuvant therapy
12. Current known infection with HIV, hepatitis B virus, or hepatitis C virus
13. Severe uncontrolled concomitant disease that would contraindicate the use of any of the investigational drugs used in this study or that would put the patient at high risk for treatment-related complications: such as uncontrolled systemic disease (e.g., pulmonary [including interstitial lung disease]) or metabolic disease, wound healing disorders, ulcers, or bone fractures
14. Pregnant or lactating women
15. Dyspnea at rest due to complications of advanced malignancy or other disease requiring continuous oxygen therapy
16. Major surgical procedure or significant traumatic injury within 14 days prior to first dose of study drug administration (dosing) or anticipation of need for major surgery during the course of study treatment. Note: Should surgery be necessary during the course of the study, patients should be allowed to recover for a minimum of 14 days prior to subsequent study treatment.
17. Receipt of IV antibiotics for infection within 14 days prior to first dose of study drug administration

"Use of this document is governed by the terms of use on the first page of this document."

18. Current chronic daily treatment (continuous for > 3 months) with corticosteroids (dose ≥ 10 mg/day methylprednisolone), excluding inhaled steroids
19. Known hypersensitivity to any of the study medications or to excipients of recombinant human or humanized antibodies
20. History of receiving any investigational treatment within 28 days prior to first dose of study drug administration (dosing)
21. Assessed by the investigator as unable or unwilling to comply with the requirements of the protocol
22. Concurrent participation in any interventional clinical trial

Length of Study

The study duration will be approximately 42 months, including 18 months of recruitment and 24 months of treatment/follow-up after the last patient has been enrolled.

End of Study

The primary objective of this study is the safety outcome. The study will end at the time of the cutoff for the final analysis, **24 months** after the last patient has been enrolled, or all patients in the study have withdrawn consent, died, or if the study is prematurely terminated by the Sponsor, whichever occurs first.

All patients who are still receiving study treatment at time of cutoff for the final analysis will have their post-treatment safety follow-up visit 28–35 days after the last dose of study treatment, and then will be considered as finished with their participation in the study; these patients will continue to be followed in accordance with the local standard of care.

These patients may be provided with commercial drug for continuation of treatment. This will depend on the local availability of commercial Herceptin SC, Herceptin IV, and Perjeta.

The post-study adverse events (see protocol) should be reported directly to Roche Safety Risk Management and not on the eCRF. This data collection will be restricted to reporting of serious adverse events that are believed to be related to study drug treatment.

Outcome Measures

Efficacy Outcome Measures

Efficacy outcome measures (secondary objectives) for this study are to evaluate Herceptin SC in combination with Perjeta IV plus docetaxel with respect to:

- PFS based on investigator assessment is defined as the time from first dose of study drug administration to the first radiographically documented progression of disease, as determined by the investigator using current RECIST v1.1 (see protocol) or death from any cause, whichever occurs first. Carcinomatous meningitis diagnosed by cytologic evaluation of cerebral spinal fluid will also define progressive disease (PD). Medical photography will also be allowed to monitor chest wall recurrences of subcutaneous lesions.
- OS is defined as the time from the date of first dose of study drug administration to the date of death from any cause.
- ORR is defined as a complete response (CR) or partial response (PR) determined by the investigator using RECIST v1.1 (see protocol) on two consecutive occasions ≥ 4 weeks apart. Patients with disease localized only to the bone will not be included in the analysis of objective response.

Safety Outcome Measures

The safety outcome measures for this study are as follows:

- Incidence and severity of adverse events Grade ≥ 3
- Incidence and severity of all serious adverse events
- Incidence and severity of all adverse events
- Incidence of congestive heart failure (CHF) and cardiac death

"Use of this document is governed by the terms of use on the first page of this document."

- LVEF over the course of the study and decline in LVEF from baseline
- Cause of death while in the study
- Incidence of adverse events leading to discontinuation
- Incidence of adverse events leading to dose modification
- Clinically relevant laboratory test abnormalities (see protocol for details)
- Incidence of anti-Herceptin and anti-rHuPH20 antibodies

Investigational Medicinal Products

Herceptin SC and Perjeta IV are considered to be the investigational medicinal products in this study.

Herceptin SC, Perjeta IV, and docetaxel should be administered sequentially as per table below: Herceptin SC will be administered first, followed by Perjeta IV and then docetaxel IV. An observation period of 30–60 minutes must be followed between each injection.

Herceptin SC

A fixed dose of 600 mg/5 mL Herceptin SC, irrespective of the patient's weight, will be administered throughout the treatment phase. All doses of Herceptin SC will be administered as an SC injection into the thigh by a trained health care professional over a period of 2–5 minutes. New injections should be given at least 2.5 cm from the old injection site(s) and never into areas where the skin is red, bruised, tender, or hard. During the course of treatment with Herceptin SC, other medicinal products for SC administration should preferably be injected at different sites.

Patients should be observed for 6 hours after the first injection (Cycle 1 Day 1) of Herceptin SC and for 2 hours after subsequent injections of Herceptin SC (from Cycle 2 onward) for signs or symptoms of administration-related reactions.

Perjeta IV

Perjeta will be administered as an IV infusion on Day 1 of the first treatment cycle as a loading dose of 840 mg, followed by 420 mg on Day 1 of each subsequent 3-week cycle. The medicinal products should be administered sequentially: Herceptin SC first, Perjeta IV, and then docetaxel IV, as per table below.

Initial infusions of Perjeta will be administered over 60 (±10) minutes and patients will be observed for an additional 60 minutes from the end of infusion for infusion-associated symptoms such as fever, chills, etc. Interruption or slowing of the infusion may reduce such symptoms. If the infusion is well tolerated, subsequent infusions may be administered over 30–60 (±10) minutes, with patients observed for an additional 30 minutes from the end of the infusion for infusion-associated symptoms.

Non-Investigational Medicinal Products

Docetaxel IV Chemotherapy

Docetaxel is considered to be a non-investigational medicinal product in this study.

Docetaxel will be administered in line with the respective product information and/or recognized clinical practice guidelines. It will be administered after Herceptin SC and Perjeta IV (see table below).

"Use of this document is governed by the terms of use on the first page of this document."

Summary of Study Treatment Dose and Schedule

Cycle	Herceptin SC	Perjeta IV	Docetaxel IV
Cycle 1	Day 1:<br><br>A fixed dose of **600 mg/5 mL** Herceptin SC administered over 2–5 minutes	Day 1: Perjeta IV administration **60 minutes** after the end of the Herceptin SC administration<br>**840 mg loading dose,** administered over 60 minutes	Day 1: Docetaxel IV administration **60 minutes** after the end of the Perjeta IV administration<br>**75 mg/m^2** administered over 60 minutes Cycle 1
	Patients to be observed **for 6 hours after the first injection of Herceptin SC**	Followed by a 60-minute observation period after the first Perjeta IV administration	Observe according to institution standards for docetaxel IV administration
From Cycle 2	Day 1:<br><br>A fixed dose of **600 mg/5 mL** Herceptin SC, administered over 2–5 minutes	Day 1: Perjeta IV administered **60 minutes** after the end of the Herceptin SC administration<br>**420 mg dose** administered over 60 minutes (or 30–60 minutes if well tolerated)	Day 1: Docetaxel IV administered **30–60 minutes** after the end of the Perjeta IV administration<br>**75 mg/m^2 (or 100 mg/m^2)**[b] administered over 60 minutes
	Patients to be observed for **2 hours after subsequent injections of Herceptin SC**	Followed by a 30 to 60-minute observation period after the subsequent Perjeta IV administration	Observe according to institution standards for docetaxel IV administration

IV = intravenous; SC = subcutaneous.

[a] One cycle every 3 weeks (21 days).

[b] Docetaxel IV for at least 6 cycles: After Cycle 6, continuation of docetaxel treatment is at the discretion of the treating physician and in agreement with the patient.

Statistical Methods

Final Safety Analysis

The safety analyses will include all enrolled patients who received at least one dose of any study drug.

The safety variables are all adverse events, adverse events Grade ≥ 3 according to the NCI CTCAE v4.0, adverse events leading to treatment interruption and discontinuation, serious adverse events, causes of deaths, incidence of CHF, incidence of cardiac adverse events Grade ≥ 3, LVEF decline (≥ 10% points from baseline to below 50%), premature discontinuation from study and treatment, and laboratory parameters. The primary interest in this study will be to estimate the incidence of adverse events Grade ≥ 3 for the treatment of Herceptin SC in combination with Perjeta and docetaxel.

The analysis of adverse events will focus on treatment-emergent adverse events (i.e., adverse events that occur during or after the first administration of study drug). Non–treatment-emergent adverse events (i.e., those that occur during screening) will be listed only during the screening period. Only the serious adverse event related to a protocol-mandated procedure will be reported and all adverse events that occurred before Day 1 (first administration) would be reported in medical history.

The incidence, type, and severity of adverse events will be summarized according to the primary System Organ Class (SOC) and within each SOC, by MedDRA preferred term.

Adverse events Grade ≥ 3, adverse events leading to treatment modification and discontinuation, and serious adverse events will be analyzed in a similar way to all adverse events. Causes of deaths will also be summarized and listed.

"Use of this document is governed by the terms of use on the first page of this document."

LVEF as well as changes from baseline over time will be analyzed using descriptive statistics for continuous variable and presented graphically over time with associated 95% CI. The percentage of patients with an LVEF decline $\geq$10% points from baseline to below 50% will be summarized.

The number of patients who prematurely discontinue from study treatment with a corresponding reason for discontinuation will be summarized and listed. The discontinuation from study will be also summarized and listed.

Descriptive statistics will be presented for cumulative study medication doses and duration of exposure.

Subgroup analysis of all grade adverse event variables will be performed for patients receiving at least one cycle of 100 mg/m^2 docetaxel.

The following subgroup analysis will be performed for LVEF decline of more than 10% points from baseline to below 50%, CHF, cardiac adverse events Grade $\geq$3:

- Other selected safety variables—race
- Known risk factors for development of cardiac related events—age, medical history of hypertension, prior treatment with anthracyclines, LVEF at baseline

The incidence and severity of administration-related reaction (ARR) adverse events will be summarized. Time to onset of the first ARRs and time from onset to resolution of ARRs will also be summarized. In addition, the ARR analyses will also be performed for each treatment cycle.

Laboratory parameters, hematology, and serum biochemistry will be presented in shift tables of NCI CTCAE v4.0 grade at baseline versus worst grade during the treatment period. The summary of laboratory parameters will also be presented by means, standard deviation, minimum, and maximum. The selected laboratory parameters will be also graphically presented over time.

Determination of Sample Size

The main objective of this safety study is the characterization of the safety profile and tolerability of Herceptin SC in combination with Perjeta IV and docetaxel based on an estimation of the incidence of adverse events. This is not a hypothesis testing study but an exploratory study with predefined precision of estimates for key safety parameters for sample size determination; there are no formal statistical hypothesis tests to be performed, and there will be no adjustments for multiplicity of endpoints or within-subgroups comparisons.

The proposed sample size to be enrolled in this study is 400 patients with the following rationale:

- The width of the 95% Pearson Clopper CI of the incidence of Grade $\geq$3 adverse events is reasonably small (71.8%, 80.3%) with 400 treated patients based on the observed incidence of Grade $\geq$3 adverse events of 76.2% for patients who were treated with Perjeta in combination with Herceptin IV in the TOC4129g/WO20698 study.
- Furthermore, with 400 treated patients on the observed incidence of cardiac events Grade $\geq$3 of 1.7% reported in the TOC4129g/WO20698 study, the width of the 95% Pearson Clopper CI of the incidence of Grade $\geq$3 cardiac adverse events is reasonably small (0.7%, 3.6%).

Interim Analyses

In addition to the final analysis, there will be an interim analysis approximately 6 months after recruitment of the last patient to determine overall safety and tolerability with special emphasis on cardiac safety and efficacy. Given the objective of the study, the Sponsor may choose to adapt the timepoint of the interim analysis or to conduct additional interim analyses if appropriate. The decision to adapt the timepoint of the interim analysis or to conduct an additional interim analysis will be documented in the Sponsor's trial master file prior to the conduct of the respective interim analysis. The interim analysis will be performed and interpreted by Sponsor study team personnel.

There will also be an annual review of safety data by the IMC.

"Use of this document is governed by the terms of use on the first page of this document."

<u>**LIST OF ABBREVIATIONS AND DEFINITIONS OF TERMS**</u>

Abbreviation	Definition
ADA	anti-drug antibody
ANC	absolute neutrophil count
ARDS	acute respiratory distress syndrome
ARR	administration-related reaction
AUC	area under the concentration curve
CHF	congestive heart failure
CR	complete response
CRO	clinical research organization
CTC	Common Terminology Criteria
CTCAE	Common Terminology Criteria for Adverse Events
C_{trough}	serum trough concentration
CVAD	central venous access device
EBC	early breast cancer
ECHO	echocardiogram
ECOG	Eastern Cooperative Oncology Group
eCRF	electronic Case Report Form
EDC	electronic data capture
EFS	event-free survival
EGFR	epidermal growth factor receptor
EPP	efficacy per protocol (population)
ER	estrogen receptor
ESMO	European Society of Medical Oncology
FDA	U.S. Food and Drug Administration
G-CSF	granulocyte colony-stimulating factor
HER	human epidermal growth factor
HER2	human epidermal growth factor 2
Herceptin IV	Herceptin for intravenous administration
Herceptin SC	Herceptin for subcutaneous administration
HR	hazard ratio
ICH	International Conference on Harmonisation
ICMJE	International Committee of Medical Journal Editors
IEC	Independent Ethics Committee
IHC	immunohistochemistry
IMC	Internal Monitoring Committee
IMP	investigational medicinal product

"Use of this document is governed by the terms of use on the first page of this document."

Abbreviation	Definition
IND	Investigational New Drug
IRB	Institutional Review Board
IRF	Independent Review Facility
ISH	in situ hybridization
IUD	intrauterine device
IUS	intrauterine system
IV	intravenous
IxRS	interactive voice/Web response system
LVEF	left ventricular ejection fraction
LVSD	left ventricular systolic dysfunction
MBC	metastatic breast cancer
MRI	magnetic resonance imaging
MUGA	multiple-gated acquisition (scan)
NCI	National Cancer Institute
NYHA	New York Heart Association
ORR	objective response rate
OS	overall survival
pCR	pathologic complete response
PD	progressive disease
PFS	progression-free survival
PI3-K	phosphatidylinositol 3-kinase
PR	partial response
Q3W	every 3 weeks
RECIST	Response Evaluation Criteria in Solid Tumors
rHuPH20	recombinant human hyaluronidase
SC	subcutaneous
SD	stable disease
SID	single-use injection device
SOC	System Organ Class
TK	tyrosine kinase
ULN	upper limit of normal
WOCBP	women of childbearing potential

"Use of this document is governed by the terms of use on the first page of this document."

1. BACKGROUND

1.1 BACKGROUND ON BREAST CANCER

Breast cancer is the most common cancer in women with a global prevalence of more than 1 million patients and approximately 450,000 deaths annually (Ferlay et al. 2010). While improved early detection and advances in systemic therapy for early-stage disease have resulted in a small decline in breast cancer mortality since 1989, metastatic breast cancer (MBC) remains largely incurable with a median survival of approximately 24 months (National Cancer Institute [NCI] 2011). Factors associated with poor survival include age $\geq$50 years, visceral disease, shorter disease-free intervals, aneuploid tumors, tumors with a high S-phase fraction, p53 accumulation, low bcl-2 expression, negative hormone receptor status, and positive human epidermal growth factor 2 (HER2) status (Chang et al. 2003).

Although the treatment of MBC is palliative rather than curative in intent, improvement in length of survival is an important treatment goal. There is a significant need for new agents with novel mechanisms of action and non-overlapping toxicity that can be combined with established treatments for breast cancer.

1.2 BACKGROUND ON STUDY TREATMENTS

1.2.1 Human Epidermal Growth Factor Receptors

Evidence suggests that dysregulation of ligands and receptors of the human epidermal growth factor (HER) receptor family are important in the pathogenesis of cancer. The HER tyrosine kinase (TK) receptor family comprises four receptors: HER1 (epidermal growth factor receptor [EGFR]), HER2, HER3, and HER4. These receptors mediate tumor cell growth, survival, and differentiation (Sundaresan et al. 1999; Yarden and Sliwkowski 2001). HER receptors normally exist as inactive monomers.

Activation of HER receptors occurs following ligand binding, leading to receptor dimerization and cell signaling through the phosphatidylinositol 3-kinase (PI3-K)/AKT pathway for promotion of tumor cell survival and through the mitogen–activated protein kinase pathway for cellular proliferation.

HER2 has no known ligand and, in a state of overexpression, can form active homodimers and initiate TK signaling without ligand stimulation. Additionally, as HER2 concentrations increase, the incidence of HER2 interactions with other receptors also increases, resulting in a broad recruitment of a number of proteins (Jones et al. 2006). Recent data obtained using micro-array technology suggest that the HER2 receptor can bind to more than 17 different proteins and may recruit proteins that other HER receptors cannot recruit. These activities highlight the promiscuity of HER2 in its ability to bind to other HER receptors and initiate TK signaling through several mechanisms (Jones et al. 2006).

"Use of this document is governed by the terms of use on the first page of this document."

In approximately 15%–20% of patients, overexpression of HER2 is observed in primary breast cancers. Overexpression of HER2 in breast cancer has been correlated with high histologic grade, increased mitotic activity, p53 mutation, negative estrogen receptor (ER) status, absence of bcl-2, and absence of lobular architecture. In addition to associations with other known negative prognostic factors, HER2 overexpression has been independently associated with worse disease-free survival and overall survival (OS) compared with tumors that do not overexpress HER2 (Pauletti et al. 2000). Approximately 65% of breast cancers are ER–positive and progesterone receptor–positive (American Cancer Society 2011).

1.2.1.1 Herceptin® (Trastuzumab) for Subcutaneous Administration

Herceptin® (trastuzumab) is a humanized monoclonal antibody directed at the HER2 receptor. Herceptin IV is indicated for the treatment of patients with HER2–positive breast cancer both in the adjuvant and metastatic settings. The addition of Herceptin IV to standard chemotherapy increases time to progressive disease (PD) or the length of progression-free survival (PFS) and improves survival when given with chemotherapy to women with HER2–positive breast cancer (Slamon et al. 2001; Romond et al. 2005).

Clinical benefits are greatest in patients with tumors that strongly overexpress HER2, graded 3+ by immunohistochemistry (IHC) and/or tumors that have HER2 gene amplification (see the Herceptin Investigator's Brochure for details on nonclinical and clinical studies).

Herceptin for subcutaneous (SC) administration (Herceptin SC) has been developed by the Sponsor to address the known limitations of intravenous (IV) administration (e.g., long administration times, treatment barriers for patients with poor venous access, continued use of port-a-cath systems). The key excipient in the SC formulation of Herceptin is the enzyme recombinant human hyaluronidase (rHuPH20), which enables larger volumes to be administered without reduced tolerability and with improved patient acceptability. Hyaluronidase acts primarily as a permeation enhancer to increase the dispersion and absorption of other co-administered drugs. SC injection of Herceptin takes significantly less time (2–5 minutes) compared with an IV infusion (Herceptin IV; 30–90 minutes) and is expected to improve treatment convenience and patient compliance. Convenience and compliance are particularly important for patients treated over prolonged periods of time, such as in the adjuvant setting.

Herceptin SC is marketed across Europe and in other areas of the world as treatment for patients with HER2-positive early breast cancer (EBC) and MBC.

1.2.1.2 Recombinant Human Hyaluronidase (rHuPH20)

The feasibility and patient acceptability of SC administration of any drug is dependent on the volume of drug that must be administered. The key excipient in the SC formulation of Herceptin is the enzyme rHuPH20, recombinant human hyaluronidase, which enables larger volumes to be administered without reduced tolerability and with improved patient

"Use of this document is governed by the terms of use on the first page of this document."

acceptability. Hyaluronidase acts primarily as a permeation enhancer to increase the dispersion and absorption of other co-administered drugs. Hyaluronidase transiently hydrolyses hyaluronan, a component of the SC matrix, leading to reduced viscosity of the extracellular matrix of the hypodermis and, thus, to an improved delivery of subcutaneously administered drugs to the systemic circulation.

rHuPH20 has been developed to improve dispersion and absorption of co-administered drugs. This recombinant human molecule has a higher purity and is associated with improved tolerability compared with the animal-derived enzyme. The rHuPH20 (Hylenex® Prescribing Information 2013) is licensed in the United States to facilitate the absorption and dispersion of drugs when given subcutaneously at doses between 50 IU and 300 IU (Frost 2007). The rHuPH20 used in the current study is produced from a second generation of the Hylenex process that has improved yield and purity. This formulation has been combined with Herceptin to allow safe and comfortable SC injection of volumes of 2–5 mL.

1.2.1.3 Clinical Studies with Herceptin for Subcutaneous Administration

Herceptin SC (formulated with rHuPH20) Vial has been studied in two clinical trials (Studies BP22023 [CP2] and BO22227 [HannaH]) that used conventional handheld syringes and hypodermic needles to administer the study drug.

A study to assess the safety of assisted- and self-administered Herceptin SC Vial and Herceptin SC single-use injection device (SID) as therapy in patients with operable HER2–positive EBC (Study MO28048 [SafeHER]) is ongoing.

The primary objective of the Phase Ib dose–finding study (BP22023 [CP2]) was to select the dose of Herceptin SC that results in exposure comparable to that achieved with an IV dose of Herceptin.

The pharmacokinetic modeling of fixed Herceptin SC dose selection indicated that a flat and a weight-based dosing strategy would result in a comparable range of exposure with a relationship to body weight that is inverse, and a fixed dose of 600 mg/5 mL would result in serum trough concentration (C_{trough}) values that are at least as high as with the every 3 weeks (Q3W) IV regimen (8-mg/kg loading dose followed by 6-mg/kg maintenance dose).

The Phase III Study BO22227 demonstrated the non-inferiority of the C_{trough} and pathologic complete response (pCR) for Herceptin SC versus Herceptin IV (Ismael et al. 2012).

The overall safety profile of trastuzumab SC (including cardiac safety) was in line with the known safety profile of the trastuzumab IV formulation. A slightly higher difference between the treatment arms were observed in the incidences of serious adverse events,

"Use of this document is governed by the terms of use on the first page of this document."

serious adverse events of infection, adverse events leading to death, cardiac serious adverse events, and adverse events leading to treatment withdrawal, which were often based on rare events. There was no pattern of events observed in the serious adverse event reports. Exploratory analyses did not indicate an association between toxicity and exposure. Although the incidence of serious adverse events was higher for trastuzumab SC, the incidence of Grade $\geq$ 3 adverse events was similar in both treatment arms. Herceptin SC injections were generally well tolerated. As expected, Herceptin-related injection-site reactions were observed at a higher rate in the Herceptin SC arm (11.1% vs. 0.3% in the SC and IV arms, respectively). With the exception of five Grade 2 adverse events, all injection-site reactions were of Grade 1 intensity. Administration-related reactions (ARRs) were more common in the Herceptin SC arm (47.8% vs. 37.2% Herceptin SC and IV arms, respectively). Erythema and cough were the primary adverse events that caused the difference observed between treatment arms. In both study arms, most adverse events ($>$97%) were of Grade 1 or 2 intensity. The incidence of Grade $\geq$3 events was comparable across arms (54% in the Herceptin SC arm vs. 52% in the Herceptin IV arm). Fourteen percent of patients in the Herceptin IV arm experienced an adverse event that met “serious” criteria (i.e., a serious adverse event) compared with 22% of patients in the Herceptin SC arm. More patients in the Herceptin SC arms had infection events reported as serious. No pattern in types of events, affected System Organ Classes (SOCs), or latency accounted for the difference in rates. No relationship between the difference in serious adverse events and Herceptin exposure (area under the curve) or body weight was found.

A higher rate of anti-drug antibodies against Herceptin was observed for the Herceptin SC formulation compared with Herceptin IV (14.6% of patients [43/295] in the Herceptin SC arm vs. 7.1% of patients [21/296] in the Herceptin IV arm). One patient treated with the IV formulation and two patients treated with the SC formulation developed neutralizing anti-Herceptin antibodies. The higher incidence of antibody development in the SC arm was not associated with adverse events or altered pharmacokinetics.

See the Herceptin Investigator’s Brochure for details on nonclinical and clinical studies.

1.2.1.4 Perjeta® (Pertuzumab)

Perjeta® (pertuzumab) is a fully humanized monoclonal antibody based on the human Ig G1(κ) framework sequences. It consists of two heavy chains (449 residues) and two light chains (214 residues). Similar to Herceptin, Perjeta is directed against the extracellular domain of HER2; however, it differs from Herceptin in the epitope-binding regions of the light chain (12 amino acid differences) and heavy chain (29 amino acid differences). As a result, Perjeta binds to an epitope within what is known as sub-domain 2 of HER2, whereas the epitope for Herceptin is localized to sub-domain 4 (Cho et al. 2003; Franklin et al. 2004).

"Use of this document is governed by the terms of use on the first page of this document."

Perjeta acts by blocking the dimerization of HER2 with other HER family members, including HER1 (EGFR), HER3, and HER4. As a result, Perjeta inhibits ligand-initiated intracellular signaling through two major signal pathways: MAP-kinase and PI3-K. Inhibition of these signaling pathways can result in growth arrest and apoptosis, respectively (Baselga 2010).

Because of the different binding sites of Perjeta and Herceptin, ligand-activated downstream signaling is blocked by Perjeta but not by Herceptin. Because of their complementary modes of action, the combination of Perjeta and Herceptin was shown to act synergistically in HER2-overexpressing diseases.

The efficacy of Perjeta was demonstrated in a multicenter, randomized, double-blind, placebo-controlled, Phase III study (WO20698/TOC4129g; CLEOPATRA) in patients with HER2-positive metastatic or locally recurrent unresectable breast cancer who had not received previous anti-HER2 therapy or chemotherapy for metastatic disease. A total of 808 patients were enrolled at sites in 25 countries. The primary efficacy endpoint was IRF-assessed PFS. Key secondary efficacy endpoints included OS and Independent Review Facility (IRF)-assessed ORR.

In this Phase III study, CLEOPATRA, patients were randomized in a 1:1 ratio to Perjeta plus Herceptin plus docetaxel (Perjeta arm; $n = 402$) or placebo plus Herceptin plus docetaxel (placebo arm; $n = 406$). Perjeta was given by IV infusion at an initial dose of 840 mg, followed every 3 weeks thereafter by a dose of 420 mg. Placebo was given by IV infusion every 3 weeks. Herceptin was given by IV infusion at an initial dose of 8 mg/kg, followed every 3 weeks thereafter by a dose of 6 mg/kg. Docetaxel was given by IV infusion at an initial dose of 75 mg/m^2 every 3 weeks. The dose of docetaxel could be escalated to 100 mg/m^2 at the investigator's discretion if the initial dose was well tolerated. Patients were treated with Perjeta/placebo and Herceptin until disease progression, withdrawal of consent, or unmanageable toxicity. Patients were treated with docetaxel for at least six cycles.

Demographic and baseline characteristics were generally well balanced between treatment groups.

At the time of the data cutoff for the primary analysis (13 May 2011), 433 IRF-confirmed PFS events had occurred in 242 patients (59.6%) in the placebo arm and 191 patients (47.5%) in the Perjeta arm.

Study WO20698/TOC4129g demonstrated a statistically significant and clinically meaningful improvement in IRF-assessed PFS in the Perjeta arm compared with the placebo arm (HR: 0.62; 95% CI: 0.51, 0.75; $p < 0.0001$), with an increase of 6.1 months in median PFS (12.4 months in the placebo arm vs. 18.5 months in the Perjeta arm). The Kaplan-Meier curve (see Figure 1; Baselga et al. 2012) shows an early separation beginning at the first tumor assessment (9 weeks), which is maintained from this point

"Use of this document is governed by the terms of use on the first page of this document."

onward. Primary efficacy results for OS and IRF-assessed ORR supported the PFS benefit. Analyses of PFS by clinically relevant patient subgroups suggested that the benefit of Perjeta in combination with Herceptin and docetaxel was observed consistently in all prespecified subgroups tested, including those based on geographic region, prior treatment, age, race, presence of visceral disease, hormone receptor status, and HER2 IHC or fluorescent in situ hybridization (ISH) status.

After 1 year of additional follow-up (14 May 2012 data cutoff), the efficacy results (primary efficacy endpoint of IRF-assessed PFS) for Study WO20698/TOC4129g remained robust, confirming the results of the primary analysis and supporting the current labeling for Perjeta.

In addition, a second interim analysis of the secondary efficacy endpoint of OS achieved statistical significance. The main efficacy findings are as follows:

- The second interim analysis of OS crossed the predefined stopping boundary for statistical significance ($p \leq 0.0138$), demonstrating that treatment with Perjeta arm significantly improved OS compared with placebo arm (HR: 0.66; 95% CI: 0.52, 0.84; $p = 0.0008$). Thus, Study WO20698/TOC4129g met its OS endpoint (14 May 2012 data cutoff).
- Subgroup analyses of OS were consistent with the overall OS analysis (14 May 2012 data cutoff).
- An updated analysis of PFS (14 May 2012 data cutoff), on the basis of the investigator's assessment alone, demonstrated that the PFS benefit observed at the primary analysis was maintained after an additional year of follow-up. An HR of 0.69 and an increase of 6.3 months in median PFS (from 12.4 months in the placebo arm to 18.7 months in the Perjeta arm) were consistent with those from the primary analysis of IRF-assessed PFS (13 May 2011 data cutoff) and provide further confirmation of the positive benefit-risk ratio of treatment with Perjeta plus Herceptin plus docetaxel in patients with HER2-positive metastatic or locally recurrent unresectable breast cancer.
- Under a fixed-sequence testing hierarchy implemented for confirmatory testing of secondary endpoints (OS, IRF-assessed ORR), the updated results for IRF-assessed ORR were considered statistically significant (ORR: 69.3% in placebo + trastuzumab + docetaxel vs. 80.2% in Perjeta arm + trastuzumab + docetaxel; difference in response rates between treatment arms of 10.8; 95% CI: 4.2, 17.5; $p = 0.001$).

"Use of this document is governed by the terms of use on the first page of this document."

Figure 1 Independently Assessed Progression-Free Survival in Study TOC4129g/WO20698

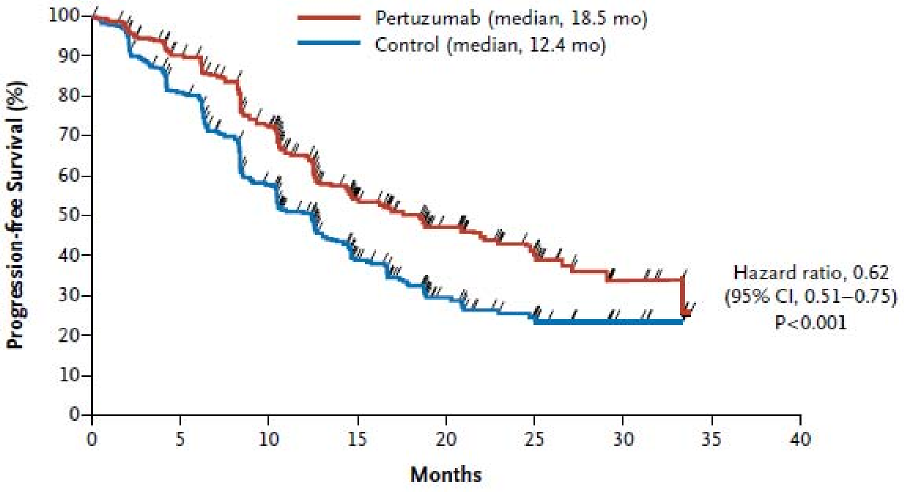

No. at Risk

	0	5	10	15	20	25	30	35	40
Pertuzumab	402	345	267	139	83	32	10	0	0
Control	406	311	209	93	42	17	7	0	0

mo = month; No. = number.

The combination of Perjeta and Herceptin IV plus docetaxel increased rates of diarrhea, rash, mucosal inflammation, febrile neutropenia, and dry skin compared with the control group. These adverse events were primarily Grades 1 and 2, manageable, and occurred during docetaxel therapy. There was no increase in cardiac adverse events or left ventricular systolic dysfunction (LVSD; Baselga et al. 2012).

LVSD, any grade, was reported more frequently in the control group than in the Perjeta group (8.3% vs. 4.4%, respectively). LVSD of Grade 3 or higher was reported in 2.8% of the patients in the control group and in 1.2% of the patients in the Perjeta group. Among patients in whom the LVEF was assessed at baseline and post-baseline, 6.6% of patients in the control group and 3.8% of patients in the Perjeta group had declines of ≥10% from baseline that resulted in an LVEF of <50% (Baselga et al. 2012).

In the placebo plus Herceptin plus docetaxel arm, 45.8% of patients experienced neutropenia and 7.6% experienced febrile neutropenia of Grade ≥3 compared with 48.9% and 13.8% of patients, respectively, in the Perjeta and Herceptin plus docetaxel arm (Baselga et al. 2012).

After an additional year of follow-up (14 May 2012 data cutoff), data from Study WO20698/TOC4129g indicate that the safety profile of Perjeta arm is essentially unchanged. Safety findings in the Perjeta arm remain comparable to those in the placebo arm, apart from a higher incidence of Grade 1 or 2 diarrhea, rash, mucosal inflammation, pruritus, dry skin, and Grade 3 or 4 febrile neutropenia. Although the

"Use of this document is governed by the terms of use on the first page of this document."

incidence of serious adverse events remained higher in the Perjeta arm (29.0% placebo arm vs. 36.3% Perjeta arm), the proportion of patients who discontinued therapy because of an adverse event or death was 9.0% in both treatment arms. The incidence of treatment-related deaths remained low in both arms ($\leq$1.5% of patients) with no new treatment-related deaths reported in the Perjeta arm since the first data cutoff date.

Importantly, despite dual targeting of the same HER2-receptor pathway with an additional year of follow-up, the incidence and severity of cardiac toxicity in the Perjeta arm remained similar to those in the placebo arm. The incidence of cardiac adverse events (all grades) was 16.4% in the placebo arm and 14.5% in the Perjeta arm with LVSD (all grades) being the most frequently reported event (8.3% vs. 4.4% in the placebo and Perjeta arm).

Declines in LVEF by $\geq$10% points from baseline and to $<$50% were reported in 6.6% and 3.8% of patients in the placebo and Perjeta arms, respectively. Seventy-two percent (placebo arm) and 86.7% (Perjeta arm) of those patients recovered to a value $\geq$50%. The incidence of symptomatic LVSD was low, occurring in 1.8% (n = 7) versus 1.0% (n = 4) of patients in the placebo and Perjeta arms, respectively. In 8 out of 11 patients, the symptomatic LVSD had resolved at data cutoff (Swain et al. 2013a).

The results of the final OS analysis after a median follow-up of 50 months were presented at the European Society of Medical Oncology (ESMO) 2014; median OS has now been reached for patients receiving the Perjeta regimen. Adding Perjeta IV to Herceptin IV and docetaxel IV extended OS by 15.7 months compared with Herceptin and docetaxel (median OS: 56.5 vs. 40.8 months). The risk of death was reduced by 32% for patients who received the Perjeta regimen compared with those who received Herceptin IV and docetaxel (HR = 0.68, 95% CI: 0.56, 0.84; p = 0.0002). The OS results were consistent across patient subgroups. The median PFS improvement of more than 6 months was maintained (median PFS of 18.7 months for people who received Perjeta, Herceptin IV, and docetaxel compared with 12.4 months for those who received Herceptin IV and docetaxel). Patients who received the Perjeta regimen had a 32% reduction in the risk of their disease worsening or death (PFS; HR = 0.68, 95% CI: 0.58, 0.80) compared with patients who received Herceptin and docetaxel. The safety profile of Perjeta was consistent with that observed previously in the CLEOPATRA study, including long-term cardiac safety. No new safety signals were observed.

"Use of this document is governed by the terms of use on the first page of this document."

Figure 2 Final Overall Survival Analysis

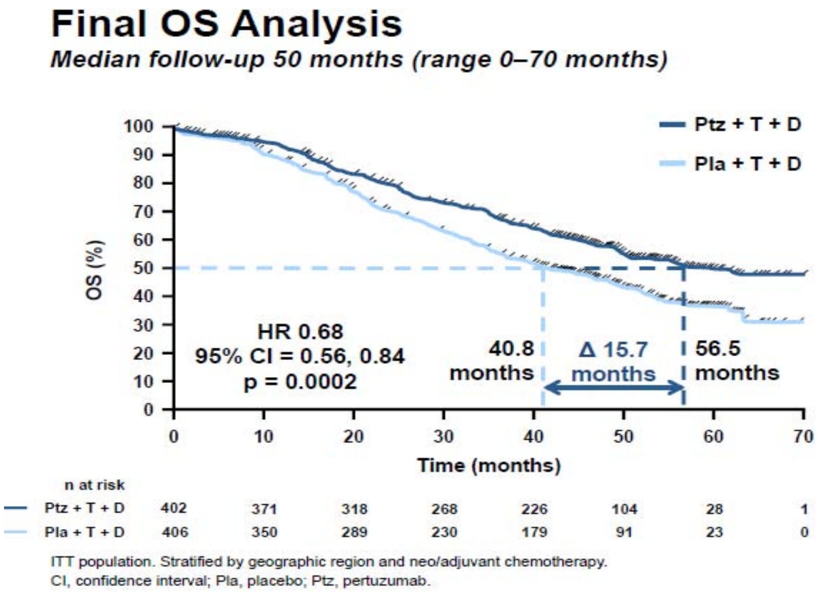

D = docetaxel; ESMO = European Society of Medical Oncology; HR = hazard ratio; ITT = intent-to-treat; OS = overall survival; Pla = placebo; Ptz = pertuzumab; T = trastuzumab.
S. Swain ESMO 2014

The incorporation of Herceptin SC in place of Herceptin IV in the standard of care regimen of Herceptin IV, Perjeta IV, and chemotherapy should have a low additional impact on tolerability and safety, and together with rigorous monitoring of the known toxicities of the agents involved, the proposed study poses an acceptable risk in this patient population with advanced breast cancer.

The complementary mechanisms and good tolerability profile of each of the HER2-directed antibodies, Herceptin and Perjeta, strongly supported by the results of the randomized, double-blind Phase III TOC4129g/WO20698 study, provide a strong rationale to further explore and better characterize the safety and tolerability profile of the combination of the anti-HER2 antibodies Herceptin and Perjeta given in combination with docetaxel.

See the Perjeta Investigator’s Brochure for details on nonclinical and clinical studies.

1.2.2 Docetaxel

Taxanes are anti-neoplastic agents that bind to free tubulin within the cell and promote the assembly of tubulin into stable microtubules while simultaneously inhibiting their disassembly. This mode of action leads to the production of microtubule bundles without normal function and to the stabilization of microtubules, blocking cells in the M-phase of the cell cycle and leading to cell death. Extensive Phase II and Phase III data have led

"Use of this document is governed by the terms of use on the first page of this document."

to regulatory approvals for the use of docetaxel either in combination or as monotherapy for the treatment of breast cancer.

Docetaxel is a semi-synthetic analog of paclitaxel, which was the first taxane to be identified. Both Herceptin and Perjeta have been successfully administered with docetaxel in doses ranging between 60 mg/m^2 and 100 mg/m^2.

When administered with Herceptin and Perjeta, the recommended initial dose of docetaxel is 75 mg/m^2, administered thereafter on an every 3-week schedule. The dose of docetaxel may be escalated to 100 mg/m^2 at the investigator's discretion on subsequent cycles if the initial dose is well tolerated.

See the local prescribing information for docetaxel for details on nonclinical and clinical studies.

1.3 STUDY RATIONALE AND BENEFIT-RISK ASSESSMENT

This is an open-label, single-arm, multicenter, Phase IIIb study with the primary objective to evaluate the safety and tolerability of Herceptin SC in combination with Perjeta IV plus docetaxel in patients with HER2–positive advanced (metastatic or locally recurrent) breast cancer. Improved PFS and OS, along with an acceptable safety profile, have been demonstrated in clinical trials of Perjeta in combination with Herceptin IV and docetaxel in this patient population (Study TOC4129g/WO20698). In parallel, the Phase III Study BO22227 demonstrated the non-inferiority of Herceptin SC compared with Herceptin IV in patients with EBC in the neoadjuvant and adjuvant settings. Combination therapy of Perjeta and Herceptin IV plus docetaxel has become the new standard of care for these patients. Since Herceptin SC has not been investigated in combination with Perjeta IV in patients with MBC, no safety and tolerability data are available, but the similar setup of the current study and CLEOPATRA (TOC4129g/WO20698) study (addendum CSR 1053649) will allow an indirect (historical) comparison of safety data results.

The present study will be conducted as a post-authorization safety measure to generate additional safety and tolerability data for Herceptin SC in combination with Perjeta IV and docetaxel in the HER2-positive advanced breast cancer setting, a triplet regimen that is approved and is the standard of care in this indication.

2. OBJECTIVES

2.1 PRIMARY OBJECTIVE

The primary objective for this study is to evaluate the safety and tolerability of Herceptin SC in combination with Perjeta IV plus docetaxel in patients with HER2–positive advanced (metastatic or locally recurrent) breast cancer.

"Use of this document is governed by the terms of use on the first page of this document."

- Overall safety profile as determined by adverse events of any grade of severity, and adverse events Grade ≥ 3 according to National Cancer Institute Common Terminology Criteria for Adverse Events (NCI CTCAE), Version 4.0 and cardiac function will be assessed including the following: cardiac events Grade ≥ 3, congestive heart failure (CHF), and cardiac death (see Section 6.5)

2.2 SECONDARY OBJECTIVES

The secondary objectives for this study are to evaluate Herceptin SC in combination with Perjeta IV plus docetaxel with respect to:

- Efficacy parameters (see Section 6.4)

  PFS

  OS

  Objective Response Rate (ORR)

- Incidence of anti-Herceptin and anti-rHuPH20 antibody formation

3. STUDY DESIGN

3.1 DESCRIPTION OF STUDY

This is an open-label, single-arm, multicenter, Phase IIIb study to evaluate the safety and tolerability of Herceptin SC in combination with Perjeta IV plus docetaxel (see Figure 3). Patients with HER2–positive advanced breast cancer (metastatic or locally recurrent) who have not previously received systemic non–hormonal anti-cancer therapy in the metastatic setting are eligible to participate in the study. Enrollment is defined as first dose of study drug administration.

Four hundred patients are planned to be enrolled into the study at approximately 110 centers worldwide. Details of the study treatment are given in Section 4.3.

Treatment Period

Every 3 weeks (21 days) the patient will receive Herceptin SC (fixed dose of 600 mg/5 mL), Perjeta IV (loading dose of 840 mg followed by 420 mg on Day 1 of each subsequent cycle), and docetaxel IV (at least six cycles with recommended initial dose of 75 mg/m^2). After Cycle 6, continuation of docetaxel treatment is at the discretion of the treating physician in agreement with the patient. The dose of docetaxel may be escalated to 100 mg/m^2 at the investigator's discretion on subsequent cycles if the initial dose is well tolerated.

End of Treatment

End of treatment for each patient is defined as receiving study medication until disease progression, unacceptable toxicity, withdrawal of consent, death, or predefined study end, whichever occurs first.

"Use of this document is governed by the terms of use on the first page of this document."

Post-Treatment Follow-up

If the patient discontinues all study treatments because of unacceptable toxicity, she will enter in follow-up and will have tumor assessments every 9 weeks (±3 days) until disease progression or predefined end of the study (see Section 3.2), whichever occurs first.

In addition, patients with disease progression will continue to be followed until death, loss to follow-up, withdrawal of consent, predefined study end (see Section 3.2), or study termination by Roche.

A Schedule of Assessments is provided in Appendix 1.

"Use of this document is governed by the terms of use on the first page of this document."

Figure 3 Study Design

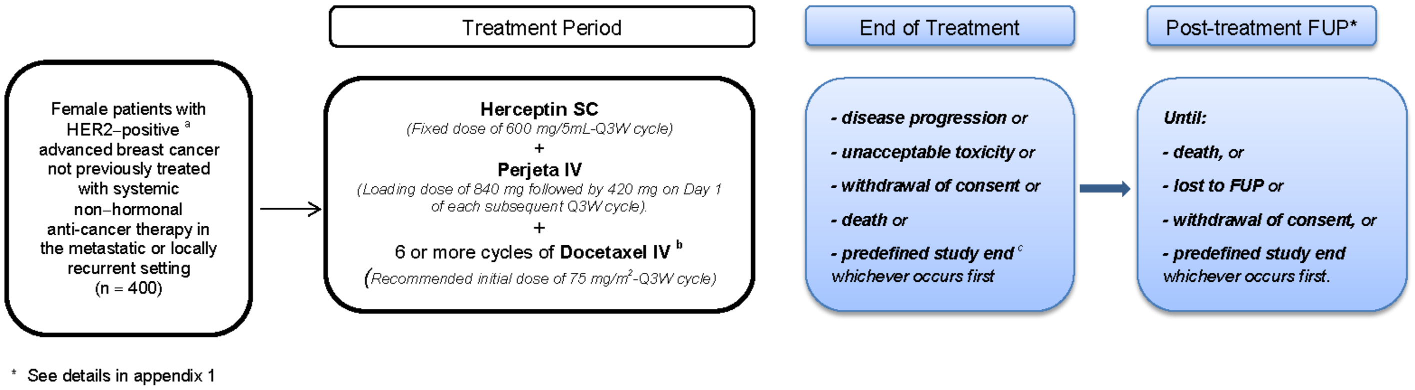

FUP = follow-up; HER2 = human epidermal growth factor receptor 2; IV = intravenous; Q3W = every 3 weeks; SC = subcutaneous.

See Table 1 for dose administration guidelines.

[a] Defined as either immunohistochemistry 3+ or in situ hybridization positive.

[b] After Cycle 6, continuation of docetaxel treatment is at the discretion of the treating physician in agreement with the patient. The dose of docetaxel may be escalated to 100 mg/m^2 at the investigator's discretion on subsequent cycles if the initial dose is well tolerated.

[c] Cutoff of Final Safety Analysis will occur 24 months after the last patients is recruited (see Section 3.2).

"Use of this document is governed by the terms of use on the first page of this document."

3.1.1 Internal Monitoring Committee

An Internal Monitoring Committee (IMC) will be established for the study with Roche members who are independent from the BO29159 study team. The IMC membership will include representatives from clinical science, safety science, and biostatistics. Specific policies on the operation of the IMC will be documented in an IMC Charter.

The IMC will meet on a regular basis over the course of the study, with the first review when 50 patients are treated for at least 3 cycles, and annually thereafter, as specified in the IMC Charter. The IMC may also meet on an unscheduled basis if any unexpected safety concerns arise.

The details of the IMC will be provided in a separate IMC Charter document.

3.2 END OF STUDY

The primary objective of this study is the safety outcome. The study will end at the time of the cutoff for the final analysis, **24 months** after the last patient has been enrolled, or all patients in the study have withdrawn consent, died, or if the study is prematurely terminated by the Sponsor, whichever occurs first.

All patients who are still receiving study treatment at time of cutoff for the final analysis will have their post-treatment safety follow-up visit 28–35 days after the last dose of study treatment, and then will be considered as finished with their participation in the study; these patients will continue to be followed in accordance with the local standard of care.

These patients may be provided with commercial drug for continuation of treatment. This will depend on the local availability of commercial Herceptin SC, Herceptin IV, and Perjeta.

The post-study adverse events (see Section 5.6) should be reported directly to Roche Safety Risk Management and not on the eCRF. This data collection will be restricted to reporting of serious adverse events that are believed to be related to study drug treatment.

3.3 RATIONALE FOR STUDY DESIGN

This is an open-label, single-arm, multicenter, Phase IIIb study with the primary objective to evaluate the safety and tolerability of Herceptin SC in combination with Perjeta IV plus docetaxel in patients with HER2–positive advanced (metastatic or locally recurrent) breast cancer. Improved PFS and OS, along with an acceptable safety profile, have been demonstrated in clinical trials of Perjeta in combination with Herceptin IV and docetaxel in this patient population (Study TOC4129g/WO20698). In parallel, the Phase III Study BO22227 demonstrated the non-inferiority of Herceptin SC compared with Herceptin IV in patients with EBC in the neoadjuvant and adjuvant settings. Combination therapy of Perjeta and Herceptin IV plus docetaxel has become the new

"Use of this document is governed by the terms of use on the first page of this document."

standard of care for these patients. Since Herceptin SC has not been investigated in combination with Perjeta in patients with MBC, no safety and tolerability data are available, but the similar setup of the current study and CLEOPATRA (TOC4129g/WO20698) study (addendum CSR 1053649) will allow an indirect (historical) comparison of safety data results.

As this is a safety study where all patients must receive the active treatment, the study design will be open-label and non-randomized.

Safety and tolerability will be carefully evaluated, and the type of data collected and the frequency with which patients are monitored will ensure the safety of the patients at all times, as well as fulfill local and international regulatory requirements.

3.4 OUTCOME MEASURES

3.4.1 Efficacy Outcome Measures

Efficacy outcome measures (secondary objectives) for this study are to evaluate Herceptin SC in combination with Perjeta IV plus docetaxel with respect to:

- PFS based on investigator assessment is defined as the time from first dose of study drug administration to the first radiographically documented progression of disease, as determined by the investigator using current Response Evaluation Criteria in Solid Tumors (RECIST), Version 1.1 (see Appendix 5; Eisenhauer et al. 2009) or death from any cause, whichever occurs first. Carcinomatous meningitis diagnosed by cytologic evaluation of cerebral spinal fluid will also define PD. Medical photography will also be allowed to monitor chest wall recurrences of subcutaneous lesions.
- OS is defined as the time from the date of first dose of study drug administration to the date of death from any cause.
- ORR is defined as a complete response (CR) or partial response (PR) determined by the investigator using RECIST v1.1 (see Appendix 5; Eisenhauer et al. 2009) on two consecutive occasions ≥4 weeks apart. Patients with disease localized only to the bone will not be included in the analysis of objective response.

3.4.2 Safety Outcome Measures

The safety outcome measures for this study are as follows:

- Incidence and severity of adverse events Grade ≥3
- Incidence and severity of all serious adverse events
- Incidence and severity of all adverse events
- Incidence of CHF and cardiac death
- LVEF over the course of the study and decline in LVEF from baseline
- Cause of death while in the study
- Incidence of adverse events leading to discontinuation

"Use of this document is governed by the terms of use on the first page of this document."

- Incidence of adverse events leading to dose modification
- Clinically relevant laboratory test abnormalities (see Section 5.3.5.5 for details)
- Incidence of anti-Herceptin and anti-rHuPH20 antibodies

Grading of non-serious and serious adverse events is performed according to NCI CTCAE, v4.0, (published 28 May 2009 and v4.03: 14 June 2010). Heart failures will also be classified according to the New York Heart Association (NYHA) functional classification system (Weatherall et al. 1996; see Appendix 4).

4. MATERIALS AND METHODS

4.1 PATIENTS

The target population for this study is patients with HER2–positive advanced (metastatic or locally recurrent) breast cancer without previous systemic non-hormonal anti-cancer therapy for the metastatic or locally recurrent disease.

4.1.1 Inclusion Criteria

Patients must meet the following criteria for study entry according to the timing specified in the Schedule of Assessments (see Appendix 1):

1. Signed, written informed consent approved by the relevant Institutional Review Board (IRB) or Independent Ethics Committee (IEC)
2. Female patients aged 18 years or older
3. Histologically or cytologically confirmed and documented adenocarcinoma of the breast with metastatic or locally recurrent disease not amenable to curative resection. Patients with measurable and/or non-measurable disease evaluable according to RECIST v1.1 (see Appendix 5) are eligible.

   Notes:

   - Patients with only bone metastases are eligible provided that they have some bone metastases that have not been previously irradiated and tumor tissue samples from the primary tumor are available for local HER2 testing.
   - Locally recurrent disease must not be amenable to resection with curative intent.
   - Patients with de novo Stage IV disease are eligible.
4. HER2-positive disease (defined as either IHC 3+ or ISH positive) as assessed by local laboratory on primary tumor or metastatic site if primary tumor not available (positive ISH is defined as a ratio of $\geq$2.0 for the number of HER2 gene copies to the number of signals for CEP17, or for single probe tests, a HER2 gene count $>$4).
5. Eastern Cooperative Oncology Group (ECOG) Performance Status 0 or 1 (see Appendix 3)
6. LVEF of at least 50%

"Use of this document is governed by the terms of use on the first page of this document."

7. Negative serum pregnancy test result in women of childbearing potential (WOCBP; defined as premenopausal or $<$12 months of amenorrhea postmenopause and who have not undergone surgical sterilization)

8. WOCBP must agree to use a highly effective, non-hormonal form of contraception (such as surgical sterilization) or two effective forms of non-hormonal contraception or true abstinence during the treatment period and for at least 7 months after discontinuation of study treatment (see Section 5.1.4, Pregnancy, for details).

9. Life expectancy of at least 12 weeks

4.1.2 Exclusion Criteria

Patients who meet any of the following criteria will be excluded from study entry. Assessments must be performed according to the timing specified in the Schedule of Assessments (see Appendix 1):

1. Previous systemic non-hormonal anti-cancer therapy for the metastatic or locally recurrent disease. Note: Prior to study entry, up to two lines of hormonal therapy for metastatic or locally recurrent disease are permitted, one of which may be in combination with everolimus.

2. Disease-free interval of *less than* 6 months from completion of adjuvant or neoadjuvant systemic non-hormonal treatment to recurrence of breast cancer

3. Previous approved or investigative anti–HER2 agents as neoadjuvant or adjuvant therapy for any breast cancer treatment, except Herceptin

4. History of persistent Grade 2 or higher hematological toxicity resulting from previous adjuvant or neoadjuvant therapy

5. Patients with radiographic evidence of CNS metastases as assessed by computed tomography or magnetic resonance imaging (MRI) that are not well controlled (i.e., are symptomatic or require control with continuous corticosteroid therapy [e.g., dexamethasone]). Note: Patients with CNS metastases are permitted to participate in the study if they have been stable in the 3 months prior to screening (as assessed by the investigator) after receiving local therapy (irradiation, surgery, etc.) but have not received anti-HER2 therapy.

6. Current peripheral neuropathy of Grade 3 or greater

7. History of other malignancy within the last 5 years prior to first dose of study drug administration (dosing), except for carcinoma in situ of the cervix or basal cell carcinoma

8. Inadequate organ function, evidenced by the following laboratory results:

    a) ANC $<$1500 cells/mm^3

    b) Platelet count $<$100,000 cells/mm^3

    c) Hemoglobin $<$9 g/dL

    d) Total bilirubin greater than the upper limit of normal (ULN; unless the patient has documented Gilbert's syndrome)

    e) AST (SGOT) or ALT (SGPT) $>$2.5 $\times$ ULN

"Use of this document is governed by the terms of use on the first page of this document."

f) AST (SGOT) or ALT (SGPT) $>1.5 \times$ ULN with concurrent serum alkaline phosphatase $>2.5 \times$ ULN. Serum alkaline phosphatase may be $>2.5 \times$ ULN only if bone metastases are present and AST (SGOT) and ALT (SGPT) are $<1.5 \times$ ULN

g) Serum creatinine >2.0 mg/dL or 177 µmol/L

h) INR and aPTT or PTT $>1.5 \times$ ULN (unless on therapeutic anticoagulation)

9. Uncontrolled hypertension (systolic blood pressure >150 mmHg and/or diastolic blood pressure >100 mmHg) with or without medication

10. Clinically significant cardiovascular disease as follows:

    a) Cerebrovascular accident/stroke or myocardial infarction within 6 months prior to first study medication, or

    b) Unstable angina, or

    c) History of or active CHF of any NYHA criteria, or

    d) History of or ongoing serious cardiac arrhythmia requiring medication (except controlled atrial fibrillation or paroxysmal supraventricular tachycardia)

11. History of LVEF decline to below 50% during or after prior Herceptin neoadjuvant or adjuvant therapy

12. Current known infection with HIV, hepatitis B virus, or hepatitis C virus

13. Severe uncontrolled concomitant disease that would contraindicate the use of any of the investigational drugs used in this study or that would put the patient at high risk for treatment-related complications: such as uncontrolled systemic disease (e.g., pulmonary [including interstitial lung disease]) or metabolic disease, wound healing disorders, ulcers, or bone fractures

14. Pregnant or lactating women

15. Dyspnea at rest due to complications of advanced malignancy or other disease requiring continuous oxygen therapy

16. Major surgical procedure or significant traumatic injury within 14 days prior to first dose of study drug administration (dosing) or anticipation of need for major surgery during the course of study treatment. Note: Should surgery be necessary during the course of the study, patients should be allowed to recover for a minimum of 14 days prior to subsequent study treatment.

17. Receipt of IV antibiotics for infection within 14 days prior to first dose of study drug administration

18. Current chronic daily treatment (continuous for >3 months) with corticosteroids (dose ≥ 10 mg/day methylprednisolone), excluding inhaled steroids

19. Known hypersensitivity to any of the study medications or to excipients of recombinant human or humanized antibodies

20. History of receiving any investigational treatment within 28 days prior to first dose of study drug administration (dosing)

"Use of this document is governed by the terms of use on the first page of this document."

21. Assessed by the investigator as unable or unwilling to comply with the requirements of the protocol
22. Concurrent participation in any interventional clinical trial

4.2 METHOD OF TREATMENT ASSIGNMENT AND BLINDING

Not applicable; this is an open-label, non-randomized single-arm study.

4.3 STUDY TREATMENT

4.3.1 Formulation, Packaging, and Handling

Study drug packaging will be overseen by the Sponsor's Clinical Trial Supplies department and bear a label with the identification required by local law, the protocol number, drug identification, and dosage.

The packaging and labeling of the study drug will be in accordance with Sponsor standards and local regulations.

The study drug must be stored according to the details on the Product Information. The drug label indicates the storage temperature.

Local packaging in some countries may be different.

Upon arrival of investigational products at the site, site personnel should check them for damage, verify proper identity, quantity, integrity of seals, and temperature conditions and report any deviations or product complaints upon discovery.

4.3.1.1 Subcutaneous Herceptin

Herceptin for SC administration will be supplied in a vial, ready-to-use liquid formulation with a nominal content of 600 mg/5 mL Herceptin. The drug product contains rHuPH20, histidine/histidine-HCl (buffer), α,α-trehalose dehydrate (bulking agent), methionine (stabilizer), and polysorbate 20 (stabilizer/emulsifier) in water for injection at a pH of 5.5 ± 0.6. rHuPH20, the human recombinant hyaluronidase (manufactured in a Chinese hamster ovary cell line), is a permeation enhancer to allow SC administration of higher volumes.

The recommended storage conditions are 2–8°C, protected from light. Batch-specific details and information on shelf life are given in the packaging label.

For further details, see the Herceptin Investigator's Brochure.

4.3.1.2 Intravenous Perjeta

Perjeta IV will be supplied by the Sponsor to the investigational sites.

"Use of this document is governed by the terms of use on the first page of this document."

Perjeta drug product is provided as a single-use formulation containing 30 mg/mL Perjeta in 20 mM L-histidine acetate (pH 6.0), 120 mM sucrose, and 0.02% polysorbate 20. Each 20-mL vial contains 420 mg of Perjeta (14.0 mL/vial).

Upon receipt, Perjeta vials are to be refrigerated at 2–8°C (36–46°F) until use. Perjeta vials should not be used beyond the expiration date provided by the manufacturer. Because the formulation does not contain a preservative, the vial seal may be punctured only once. Any remaining solution should be discarded. Vial contents should be protected from light and should not be frozen.

For further details, see the Perjeta Investigator's Brochure.

4.3.1.3 Intravenous Docetaxel

Commercial docetaxel for IV administration will be obtained locally by the investigational sites.

For further details, see the local prescribing information for docetaxel and/or recognized clinical practice guidelines.

4.3.2 Dosage, Administration, and Compliance

4.3.2.1 Subcutaneous Herceptin

A fixed dose of 600 mg/5 mL Herceptin SC, irrespective of the patient's weight, will be administered throughout the treatment phase. All doses of Herceptin SC will be administered as an SC injection into the thigh by a trained health care professional over a period of 2–5 minutes. New injections should be given at least 2.5 cm from the old injection site(s) and never into areas where the skin is red, bruised, tender, or hard. During the course of treatment with Herceptin SC, other medicinal products for SC administration should preferably be injected at different sites. Patients should be observed for 6 hours after the first injection and for 2 hours after subsequent injections for signs or symptoms of ARRs.

Significant injection-related symptoms must have been resolved before any subsequent study treatment administration. Patients who experience injection-related symptoms may be premedicated with paracetamol and antihistamines for subsequent injections. Dose reductions for toxicity are not permitted.

The medicinal products should be administered sequentially on Day 1 of each cycle (every 3 weeks; see Table 1) as follows: Herceptin SC first, then Perjeta IV, and then docetaxel IV.

Herceptin SC administration may be delayed to assess or treat adverse events, such as cardiac adverse events, myelosuppression, or other events. No dose reduction will be allowed for Perjeta IV or Herceptin SC (see Section 5.1.1).

"Use of this document is governed by the terms of use on the first page of this document."

4.3.2.2 Intravenous Perjeta

Perjeta will be administered as an IV infusion on Day 1 of the first treatment cycle as a loading dose of 840 mg, followed by 420 mg on Day 1 of each subsequent 3-week cycle. Perjeta IV should be administered 60 minutes after the end of Herceptin SC administration. An observation period of 30–60 minutes is recommended after each Perjeta infusion.

Initial infusions of Perjeta will be administered over 60 (±10) minutes and patients will be observed for an additional 60 minutes from the end of infusion for infusion-associated symptoms such as fever, chills, etc. Interruption or slowing of the infusion may reduce such symptoms. If the infusion is well tolerated, subsequent infusions may be administered over 30–60 (±10) minutes with patients observed for an additional 30 minutes from the end of the infusion for infusion-associated symptoms.

Perjeta IV administration may be delayed to assess or treat adverse events such as cardiac adverse events, myelosuppression, or other events. No dose reduction will be allowed for Perjeta IV or Herceptin SC (see Section 5.1.1).

In the case of surgery during the study, there is no evidence that HER2 antibodies delay wound healing, but patients should have recovered from surgery and anesthesia (this includes liver function values) for a minimum of 14 days before antibody treatment.

4.3.2.3 Intravenous Docetaxel

Docetaxel will be administered in line with the respective product information and/or recognized clinical practice guidelines. It will be administered **after** Herceptin SC and Perjeta IV (see Table 1 and Sections 4.3.2.1 and 4.3.2.2).

When administered with Herceptin and Perjeta, the recommended initial dose of docetaxel is 75 mg/m^2, administered thereafter on a 3-week cycle. The dose of docetaxel may be escalated to 100 mg/m^2 at the investigator's discretion on subsequent cycles if the initial dose is well tolerated.

On or prior to Cycle 6, docetaxel should only be discontinued for PD or unacceptable toxicity. After Cycle 6, continuation of docetaxel treatment is at the discretion of the treating physician in agreement with the patient.

Guidelines for dosage modification and treatment interruption or discontinuation are provided in Section 5.1.1.4.

Any overdose or incorrect administration of study drug should be noted on the Study Drug Administration electronic Case Report Form (eCRF). Adverse events associated with an overdose or incorrect administration of study drug should be recorded on the Adverse Event eCRF.

"Use of this document is governed by the terms of use on the first page of this document."

Table 1 Summary of Study Treatment Dose and Schedule

Cycle [a]	Herceptin SC	Perjeta IV	Docetaxel IV
Cycle 1	Day 1:<br><br>A fixed dose of **600 mg/5 mL** Herceptin SC administered over 2–5 minutes	Day 1: Perjeta IV administration **60 minutes** after the end of the Herceptin SC administration<br>**840 mg loading dose,** administered over 60 minutes	Day 1: Docetaxel IV administration **60 minutes** after the end of the Perjeta IV administration<br>**75 mg/m^2** administered over 60 minutes Cycle 1
	Patients to be observed **for 6 hours after the first injection of Herceptin SC**	Followed by a 60-minute observation period after the first Perjeta IV administration	Observe according to institution standards for docetaxel IV administration
From Cycle 2	Day 1:<br><br>A fixed dose of **600 mg/5 mL** Herceptin SC, administered over 2–5 minutes	Day 1: Perjeta IV administered **60 minutes** after the end of the Herceptin SC administration<br>**420 mg dose** administered over 60 minutes (or 30–60 minutes if well tolerated)	Day 1: Docetaxel IV administered **30–60 minutes** after the end of the Perjeta IV administration<br>**75 mg/m^2 (or 100 mg/m^2)[b]** administered over 60 minutes
	Patients to be observed for **2 hours after subsequent injections of Herceptin SC**	Followed by a 30 to 60-minute observation period after the subsequent Perjeta IV administration	Observe according to institution standards for docetaxel IV administration

IV = intravenous; SC = subcutaneous.

[a] One cycle every 3 weeks (21 days).

[b] Docetaxel IV for at least 6 cycles: After Cycle 6, continuation of docetaxel treatment is at the discretion of the treating physician and in agreement with the patient.

Prior to disease progression, if a study patient is unable to tolerate:

- Docetaxel IV, then it is possible to continue treatment with Herceptin SC and Perjeta IV per study protocol until disease progression, or
- Perjeta IV, then it is possible to continue treatment with Herceptin SC and docetaxel IV per study protocol until disease progression, or
- Herceptin SC, then it is possible to continue treatment with docetaxel IV only per study protocol (Perjeta IV treatment to be stopped when Herceptin SC treatment is stopped) until disease progression or to switch to a non-study protocol standard of care treatment regimen (i.e., Herceptin IV, Perjeta IV, docetaxel IV, etc.)

The above options are to be based on the investigator's assessment of the patient's potential benefit relative to the risk and in the best interest of the study patient.

The Herceptin SC dose and Perjeta IV dose are fixed doses and do not need to be adjusted for body weight.

"Use of this document is governed by the terms of use on the first page of this document."

4.3.3 Investigational Medicinal Product Accountability

Herceptin SC and Perjeta IV are considered to be investigational medicinal products (IMPs) in this study.

Docetaxel IV is considered to be a non-IMP in this study.

All IMPs required for completion of this study (Herceptin SC and Perjeta IV) will be provided by the Sponsor. The investigational site will acknowledge receipt of IMP through use of the interactive voice/Web response system (IxRS) to confirm the shipment condition and content. Any damaged shipments will be replaced.

IMP will either be disposed of at the study site according to the study site's institutional standard operating procedure or returned to the Sponsor with the appropriate documentation. The site's method of IMP destruction must be agreed upon by the Sponsor. The site must obtain written authorization from the Sponsor before any IMP is destroyed, and IMP destruction must be documented on the appropriate form.

Accurate records of all IMPs and non-IMPs received at, dispensed from, returned to, and disposed of by the study site should be recorded on the Drug Inventory Log.

4.3.4 Post-Trial Access to Subcutaneous Herceptin

All patients will be treated until predefined study end (see Section 3.2), disease progression, unacceptable toxicity, withdrawal of consent, or death, whichever occurs first.

All patients who are still receiving study treatment at time of cutoff for the final analysis will have their post-treatment safety follow-up visit 28–35 days after the last dose of study treatment, and then will be considered as finished with their participation in the study; these patients will continue to be followed in accordance with the local standard of care.

The post-study adverse events (see Section 5.6) should be reported directly to Roche Safety Risk Management and not on the eCRF.

These patients may be provided with commercial drug for continuation of treatment. This will depend on the local availability of commercial Herceptin and Perjeta.

The Sponsor will offer post-trial access to the study drug Herceptin and Perjeta free of charge to eligible patients in accordance with the Roche Global Policy on Continued Access to Investigational Medicinal Product, as outlined below.

"Use of this document is governed by the terms of use on the first page of this document."

A patient will be eligible to receive study drug after the end of the study if all of the following conditions are met:

- The patient has a life-threatening or severe medical condition and requires continued study drug treatment for her well-being
- There are no appropriate alternative treatments available to the patient
- The patient and her doctor comply with and satisfy any legal or regulatory requirements that apply to them

A patient will not be eligible to receive study drug after the end of the study if any of the following conditions are met:

- The study drug is commercially marketed in the patient's country and is reasonably accessible to the patient (e.g., is covered by the patient's insurance or would not otherwise create a financial hardship for the patient)
- The Sponsor has discontinued development of the study drug or data suggest that the study drug is not effective for HER2-positive advance breast cancer (metastatic or locally recurrent)
- The Sponsor has reasonable safety concerns regarding the study drug as treatment for HER2-positive advance breast cancer (metastatic or locally recurrent)
- Provision of study drug is not permitted under the laws and regulations of the patient's country

The Roche Global Policy on Continued Access to Investigational Medicinal Product is available at the following Web site:

http://www.roche.com/policy_continued_access_to_investigational_medicines.pdf.

4.4 CONCOMITANT THERAPY

4.4.1 Permitted Therapy

Concomitant therapy includes any medication (e.g., prescription drugs, over-the-counter drugs, herbal/homeopathic remedies, nutritional supplements) used by a patient from 28 days prior to the first dose of study drug administration to the study completion/early termination visit. All concomitant medications should be reported to the investigator and recorded on the Concomitant Medications eCRF. Concomitant surgeries and procedures will be captured on separate dedicated eCRFs.

Patients should receive full supportive care when necessary, including transfusion of blood and blood products, antibiotics, etc., according to standard of care.

All protocol-allowed medications that are taken by the patient for concomitant disease(s) should be continued as necessary during the study and be recorded on the eCRF. The following list of allowed medications is provided as guidance. Treatments prescribed to patients should be adapted according to the local standard of care practice.

"Use of this document is governed by the terms of use on the first page of this document."

The following treatments/procedures are permitted:

- Paracetamol (acetaminophen) or other analgesics and diphenhydramine, chlorpheniramine, or other antihistamines can be used according to local clinical practice for the prevention and treatment of infusion reactions associated with Perjeta and/or Herceptin
- Medication to treat diarrhea (e.g., loperamide)
- Granulocyte colony stimulating factor (G-CSF) may be used according to the product license and according to the currently approved prescribing information for docetaxel and American Society of Clinical Oncology clinical guidelines (Smith et al. 2006).
- Steroids for docetaxel premedication and anti-emetics according to routine practice at each clinical site
- Inhaled steroids for asthma
- Bisphosphonates or a RANK ligand inhibitor may be given according to their product license and routine clinical practice at the investigator's discretion. Note: If these medications should be started during the trial, the patients must be assessed first for evidence of disease progression.
- Palliative surgical procedures. Any diagnostic, therapeutic, or surgical procedure performed during the study period should be recorded, including the dates, description of the procedure(s), and any clinical findings. In the case of surgery during the study, patients should have recovered from surgery and anesthesia (this includes liver function) for a minimum of 14 days before antibody treatment.
- As a precautionary measure, it is recommended but not strictly required that if patients require placement of a central venous access device (CVAD), the procedure should be done 7 days prior to first study treatment start. The date of CVAD placement should be noted in the medical record and recorded in the eCRF. Episodes of CVAD replacement should be recorded, as should CVAD-related thrombosis, infection, or dysfunction.
- Anticoagulation therapy for maintenance of patency of permanent indwelling IV catheters is permitted.
- Palliative radiotherapy. Radiotherapy is only allowed during the study treatment period for the indication of bone or breast lesions present at baseline as long as the lesion is not a target lesion. If a patient requires radiation therapy to a new lesion, that new lesion would, per RECIST, qualify as PD.
- Approved endocrine therapies only after discontinuation of chemotherapy

4.4.2 Prohibited Therapy

The following treatments are not permitted:

- Treatment with other systemic anti-cancer agents (e.g., chemotherapy, immunotherapy) or other treatments not part of the protocol-specified anti-cancer therapy. Note: Approved endocrine maintenance therapies will be permitted only after discontinuation of chemotherapy.

"Use of this document is governed by the terms of use on the first page of this document."

- Any oral, injected, or implanted hormonal methods of contraception
- Concurrent investigational agents of any type
- Initiation of herbal remedies for cancer treatment. Herbal remedies initiated prior to study entry and continuing during the study are permitted and must be reported on the appropriate eCRF.

The following treatments should be avoided because of the risk of immunosuppression:

- Chronic or high-dose oral corticosteroid therapy
- Tumor necrosis factor–α inhibitors
- Anti–T-cell antibodies

4.5 STUDY ASSESSMENTS

Details of the timing of assessments are presented in the Schedule of Assessments (see Appendix 1).

4.5.1 Informed Consent Form and Screening Log

Written informed consent for participation in the study (approved by the relevant IRB or IEC) must be obtained before performing any study-specific screening tests or evaluations. Informed Consent Forms for enrolled patients and for patients who are not subsequently enrolled will be maintained at the study site.

All screening test and evaluations must be completed and reviewed to confirm that patients meet all eligibility criteria within 28 days prior to the first administration of study medication (dosing), unless the procedures have already been conducted during this time period as part of the patient's routine clinical care. The investigator will maintain a screening log to record details of all patients screened and to confirm eligibility or record reasons for screening failure, as applicable.

4.5.2 Medical History and Demographic Data

Medical history includes clinically significant diseases, surgeries, cancer history (including prior cancer therapies and procedures), childbearing potential, and all medications used by the patient within 28 days prior to the first administration of study medication (dosing).

Women of childbearing potential are defined as premenopausal women and for women $<$12 months after the onset of menopause, unless they have undergone surgical sterilization.

The following criteria will be used to define postmenopause:

- No spontaneous menses for at least 12 months prior to study entry (e.g., spontaneous or secondary to hysterectomy) AND with a documented estradiol level in the postmenopausal range according to local institutional/laboratory standard, or

"Use of this document is governed by the terms of use on the first page of this document."

- A prior documented bilateral oophorectomy

Women failing to meet one of these criteria will be classified as premenopausal.

Demographic data will include age, sex, race, and self-reported ethnicity.

4.5.3 Physical Examination and Vital Signs

Physical examination and vital signs (pulse rate, blood pressure, body temperature), height, and weight will be measured at baseline. Any abnormality identified at baseline should be recorded on the General Medical History and Baseline Conditions eCRF.

During the treatment period, a window of ±3 days of scheduled treatment will apply to all visits. Physical examination and vital signs (pulse rate, blood pressure, body temperature recorded again after infusion during observation period, and weight) will be measured before every administration of study treatment and at post-treatment safety follow-up visit. Changes from baseline abnormalities should be recorded in patient notes. New or worsened clinically significant abnormalities should be recorded as adverse events on the Adverse Event eCRF.

Particular care will be taken with regard to cardiovascular signs and symptoms (jugular venous pressure, sinus tachycardia, tachypnea, the presence of an S3 heart sound, crackles on chest auscultation, etc.).

If applicable, vital sign assessments will be done during follow-up phase until disease progression (see Appendix 1).

4.5.4 Performance Status

Performance Status will be measured using the ECOG Performance Status scale (see Appendix 3).

It is recommended, where possible, that a patient's Performance Status will be assessed by the same person throughout the study.

Performance Status will be assessed at:

- Baseline
- Every three cycles of monoclonal antibody treatment
- 28-days post-treatment safety follow-up visit
- If applicable, at each follow-up visit until disease progression

4.5.5 Tumor and Response Evaluation

Screening tests and evaluations will be performed within **28 days** prior to the first administration of study medication (dosing), unless the procedures have already been conducted during this time period as part of the patient's routine clinical care.

"Use of this document is governed by the terms of use on the first page of this document."

Primary Tumor Receptor Status at Screening

HER2 Status: Patients must have HER2-positive status established prior to entering the study. Demonstrated evidence from previous testing is acceptable; otherwise HER2-positive status on fixed tissue blocks from the primary tumor (and/or metastatic site if primary tumor not available) will be assessed locally by IHC and/or ISH according to institutional criteria.

Hormone receptor status from primary tumor (and/or metastatic site if primary tumor not available) would be also reported.

Tumor Assessments Methodology

RECIST v1.1 will be used to evaluate response and assess PD (a summary of RECIST v1.1 is provided in Appendix 5).

The minimum screening examination should be done within 28 days before the first dose of study drug administration and should include:

- CT or MRI scan of the chest and abdomen (including liver, spleen, and adrenals) and pelvic scans. CT scans of the neck should be included if clinically indicated. At the investigator's discretion, CT scans may be repeated at any time if PD is suspected. PET scans will not be considered for assessment of efficacy at any time during the study (except as specified for bone scans in the absence of radioactive isotopes).
- CT scans should be performed with a contrast agent. The CT portion of a combination PET/CT scan is generally not performed with contrast; therefore, PET/CTs are generally not acceptable. However, if the site has acquired a high quality diagnostic CT scan, including the application of contrast agent (which may be performed with modern PET/CT scanners), the CT scan portion may be adequate for submission and evaluation. For patients with known allergies to the contrast media, it is acceptable to perform a chest CT scan without contrast and an MRI scan for the abdomen (ideally at baseline and every tumor assessment thereafter).
- CT or MRI scan of the brain and/or spine where there is clinical suspicion of CNS metastases and during the study if clinically indicated
- In case of suspicion of bone metastases, an isotope bone scan (with bone X-ray[s] as necessary) will be done at baseline. In the absence of radioactive isotopes, MRI scan (with gadolinium enhancement if required) or F18 PET scan is an acceptable form of assessment of the skeleton for the presence of bone metastases. Skeletal survey with plain X-ray is acceptable if there is no suitable alternative. It should be repeated in the event of clinical suspicion of progression of existing bone lesions and/or the appearance of new bone lesions.
- Medical photography to monitor chest wall recurrences (i.e., subcutaneous skin lesions)

"Use of this document is governed by the terms of use on the first page of this document."

All **measurable disease** must be documented at screening and re-assessed at each subsequent tumor evaluation. Tumor response will be assessed by the investigator on the basis of CT or MRI scans and (if indicated) isotope bone scan using RECIST v1.1 (see Appendix 5). An objective response is recommended to be confirmed by repeat assessments ≥4 weeks after initial documentation. Bone scan, PET scan, or plain films are not considered adequate imaging techniques to measure bone lesions.

For patients with **non-measurable disease only**, qualitative evaluation of the burden of non-measurable disease with reproducible imaging techniques will be required at the fixed timepoints in the protocol. In such cases, response to treatment should be assessed as meaningful change in the tumor burden defined as persistence, disappearance, or unequivocal progression of the tumor as per RECIST.

Consistency of consecutive CT scans, X-rays, or MRIs should be ensured during all assessments for each patient with the same technique being used for evaluating lesions throughout the treatment period (use of spiral CT or MRI is required for baseline lesions <20 mm and must be documented in medical records and used consistently throughout the study). The use of oral and IV contrast, etc. should, as long as it is clinically possible, be kept consistent. Tumor measurements should be made by the same investigator/radiologist for each patient during the study to the extent that this is feasible. In case of clinically measurable superficial (such as skin) lesions, repeated photographs should be used to document tumor response. These photographs must include a ruler for documentation purposes.

Tumor response needs to be confirmed a minimum of 4 weeks after the initial response was noted or at the next scheduled tumor assessment if it is to occur more than 4 weeks after the initial response.

See the RECIST v1.1 (Appendix 5) for further details of criteria for differentiating between response, SD, and PD.

Summary of Scheduling of Tumor Assessments

Baseline total tumor burden must be assessed within a maximum of 28 days before first dose of study drug treatment (see above for the details of minimum screening examination).

Post-baseline assessments are to be performed every 9 weeks (±3 days) from the date of first dose of study drug administration) until disease progression or end of the study, whichever occurs first. All patients should have a minimum of a chest CT scan and abdominal CT scan. PET scans will not be considered for assessments of efficacy at any time during the study (except as specified for bone scans in the absence of radioactive isotopes). If there is suspicion of disease progression based on clinical or laboratory findings before the next scheduled assessment, an unscheduled assessment should be performed. If a tumor assessment must be performed early or late,

"Use of this document is governed by the terms of use on the first page of this document."

subsequent assessments should be conducted according to the original schedule of every 9 weeks ($\pm$3 days) from the date of first dose of study drug administration.

All tumor assessments after baseline will be done within 3 days of the scheduled visit. If a patient inadvertently misses a prescribed tumor evaluation or a technical error prevents the evaluation, the patient may continue treatment until the next scheduled assessment, unless signs of clinical progression are present.

4.5.6 Cardiac Assessments

Left Ventricular Ejection Fraction Assessment

LVEF will be assessed during the screening period within 6 weeks prior to first dose of study drug and every three treatment cycles by either echocardiogram (ECHO) or multiple-gated acquisition (MUGA) scan (with ECHO as the preferred method).

Patients will be re-assessed **with the same technique** used for baseline cardiac evaluation throughout the study and, to the extent possible, the assessments should be obtained at the same institution for an individual patient.

Electrocardiograms

A standard 12-lead ECG recording must be obtained at screening within 6 weeks prior to first dose of study drug and every three cycles during the treatment period at the time of LVEF measurement.

ECGs for each patient should be obtained from the same machine whenever possible. To minimize variability, it is important that patients be in a resting position for $\geq$10 minutes prior to each ECG evaluation. Body position should be consistently maintained for each ECG evaluation to prevent changes in heart rate. Environmental distractions (e.g., television, radio, conversations) should be avoided during the pre-ECG resting period and during ECG recording.

ECGs should be performed prior to any scheduled vital sign measurements and blood draws.

For safety monitoring purposes, the investigator or designee must review, sign, and date all ECG tracings. Paper copies will be kept as part of the patient's permanent study file at the site. Where available, digital recordings will be stored at the site.

ECG characteristics, including heart rate, RR, QRS duration, PR, and QT intervals, changes in T-wave morphology, and overall ECG interpretation, will be monitored by the investigator or designee and, if clinically significant, will be reported as adverse events according to standard grading, classification, and reporting guidelines.

These cardiac assessments (standard 12-Lead ECG & LVEF) will occur:

- At screening within 6 weeks prior to the first dose of study drug

"Use of this document is governed by the terms of use on the first page of this document."

- During treatment phase at the same time every three cycles and within 7 days prior to drug administration. The same technique must be used for baseline cardiac evaluation and throughout the study.
- At the post-treatment follow-up visit (28–35 days) after the last dose of study drug
- After post-treatment follow-up visit, only LVEF will be assessed (see details in Appendix 1).

4.5.7 Laboratory Assessments

Baseline assessments have to be done prior to the first administration (Day 1).

Samples for laboratory tests will be assessed locally.

Hematology and biochemistry will be done as part of regular safety assessments at baseline (within 3 days prior to the first dose of study drug), every treatment cycle, and 28 days after the last dose of study treatment. Assessments must be performed at each cycle within 3 days (with results available) prior to the administration of study drug.

Specifically, the following will be assessed:

- Hematology: hemoglobin, hematocrit, platelet count, RBC, WBC with differential (neutrophils, lymphocytes, monocytes, eosinophils, basophils, and other cells)
- Biochemistry: sodium, potassium, calcium, chloride, magnesium, blood urea nitrogen, total protein, albumin, alkaline phosphatase, ALT, AST, gamma glutamyl transferase, LDH, total bilirubin, creatinine, blood glucose, and calculated creatinine clearance at baseline
- Coagulation: INR and aPTT or PTT. Tests should be repeated at each treatment cycle in all patients receiving therapeutic doses of anticoagulants.

Pregnancy test:

All WOCBP (premenopausal women and for women $<$12 months after the onset of menopause, unless they have undergone surgical sterilization) will have a **serum pregnancy test** at a local laboratory within 7 days prior to the first administration of study medication with result available at dosing.

For all other women, documentation must be present in medical history confirming that the patient is not of childbearing potential.

Urine pregnancy tests should be repeated during the treatment period

- Within 7 days prior to every third treatment cycle with result available at dosing starting at Cycle 3 (and as clinically indicated)
- At the post-treatment safety follow-up visit (28–35 days) after the last dose of study drug
- About 4 months and 7 months (during the closest follow-up visit) after discontinuation of study treatment until predefined end of the study (see Section 3.2)

"Use of this document is governed by the terms of use on the first page of this document."

Any positive urine pregnancy test result must be confirmed with a serum β-HCG evaluation at the local laboratory. Pregnancy test results must be available prior to the next scheduled study treatment. Women who have undergone surgical sterilization or are postmenopausal are exempt from pregnancy assessments.

WOCBP who are sexually active must agree to use a highly effective, non-hormonal form of contraception or two effective forms of non-hormonal contraception (see details in Section 5.1.4) during the study treatment and for at least 7 months after study treatment.

Unless otherwise specified, baseline tests and evaluations (e.g., physical examination) may be performed within 7 days prior to first dose of study drug administration, after confirmation of other eligibility criteria, unless the procedures have already been conducted during this time period as part of the patient's routine clinical care.

4.5.8 Herceptin Serum Concentration Assessments

The concentration of Herceptin will be characterized by measuring Herceptin with use of a validated method. Herceptin serum concentration will be assessed for all patients treated with Herceptin. The concentration of Herceptin will be used as part of the immunogenicity assessment.

The purpose of immunogenicity testing is to determine whether anti-drug antibodies (ADAs) against Herceptin or anti-rHuPH20 antibodies develop and whether these impact upon safety or efficacy. Blood sampling for immunogenicity testing will be done as per visiting schedule. All patients will be evaluated for antibodies against Herceptin and rHuPH20:

- At baseline,
- During treatment: at Cycle 2 prior to Herceptin SC drug administration, and
- During post-treatment follow-up phase: 28–35 days after the last dose of study drug and before the start of any subsequent line of treatment.

Plasma (for anti-rHuPH20 antibodies) and serum (for anti-Herceptin antibodies) samples will be shipped to a central laboratory on a continual basis. Details of sampling, storage, and shipping are described in the study's Sample Handling and Logistics Manual.

A three-tiered analytical testing approach will be performed for ADAs against both Herceptin and rHuPH20. A validated antibody-bridging ELISA will be used to screen for and confirm the presence of ADAs in patient samples, as well as to characterize and determine the titer of confirmed ADA-positive.

All immunogenicity samples will be analyzed centrally. Samples will be kept for retesting (if required) at the central laboratory and will be destroyed no later than 5 years after all study data have been collected.

"Use of this document is governed by the terms of use on the first page of this document."

See Appendix 1 for the schedule of screening and baseline assessments.

4.6 PATIENT, STUDY, AND SITE DISCONTINUATION

4.6.1 Patient Discontinuation

Patients have the right to voluntarily withdraw from the study at any time for any reason. In addition, the investigator has the right to withdraw a patient from the study at any time. Reasons for withdrawal from the study may include but are not limited to the following:

- Patient withdrawal of consent at any time
- Any medical condition that the investigator or Sponsor determines may jeopardize the patient's safety if she continues in the study
- Investigator or Sponsor determines it is in the best interest of the patient

Every effort should be made to obtain information on patients' reason for discontinuation. The primary reason for withdrawal from the study should be documented on the appropriate eCRF section. At the time of withdrawn consent of the patient, two options will be given to her:

- Stop the study treatment but still allow survival data collection only, until predefined end of the study
- Stop the study treatment with no further data collection.

Patients who withdraw from the study will not be replaced.

4.6.2 Study Treatment Discontinuation

Patients must discontinue all study drugs if they experience any of the following:

- Pregnancy
- Clinical signs and symptoms that are suggestive of symptomatic CHF
- Dyspnea or clinically significant hypotension (defined per investigator discretion)
- Symptomatic left ventricular dysfunction (NCI CTCAE v4.0 Grade 3 or 4) with a drop in LVEF consistent with cardiac failure
- Asymptomatic decline in LVEF as per cardiac algorithm (see Figure 4)

Details of discontinuation due to toxicity are given in Section 5.1.1.

Patients who discontinue all study drugs will be asked to return to the clinic for a post-treatment safety follow-up visit **28 days after the last dose** (see Appendix 1 and Section 4.5) and will undergo follow-up assessments until predefined end of the study (see Appendix 1 for details). The primary reason for each study drug discontinuation should be documented on the appropriate eCRF. Patients who discontinue study drug will not be replaced.

"Use of this document is governed by the terms of use on the first page of this document."

4.6.3 Study and Site Discontinuation

The Sponsor has the right to terminate **this study** at any time. Reasons for terminating the study may include but are not limited to the following:

- The incidence or severity of adverse events in this or other studies indicates a potential health hazard to patients.
- Patient enrollment is unsatisfactory.

The Sponsor will notify the investigator if the Sponsor decides to discontinue the study.

The Sponsor has the right **to close a site** at any time. Reasons for closing a site may include but are not limited to the following:

- Excessively slow recruitment
- Poor protocol adherence
- Inaccurate or incomplete data recording
- Non-compliance with the International Conference on Harmonisation (ICH) guideline for Good Clinical Practice
- No study activity (i.e., all patients have completed and all obligations have been fulfilled)

5. ASSESSMENT OF SAFETY

5.1 SAFETY PLAN

5.1.1 Toxicity Management Guidelines

The NCI CTCAE v4.0 will be used to grade toxicity.

Herceptin SC, Perjeta IV, and docetaxel will be given as specified in Section 4.3.2 and Table 1.

Before starting a new treatment cycle, toxicity must have resolved as specified in Sections 5.1.1.1, 5.1.1.2, 5.1.1.3, and 5.1.1.4.

Herceptin SC, Perjeta IV, and docetaxel IV administration may be delayed to assess or treat adverse events such as cardiac adverse events, myelosuppression, or other events. No dose reduction will be allowed for Herceptin SC or Perjeta IV. If any of the individual study medications must be delayed for $\geq$**1 day**, all three agents (Herceptin SC, Perjeta IV, and docetaxel IV) should be delayed for the same timeframe.

5.1.1.1 Cardiac Safety

All patients must have a baseline LVEF $\geq$50%. LVEF will be monitored regularly according to the Schedule of Assessments (see Appendix 1). If an investigator is concerned that an adverse event may be related to cardiac dysfunction, an additional LVEF measurement should be performed.

"Use of this document is governed by the terms of use on the first page of this document."

Symptomatic Left Ventricular Systolic Dysfunction will be reported on the basis of NCI CTCAE criteria v4.0 and NYHA classification (see Appendix 4 for details of the NYHA classification and left ventricular systolic dysfunction NCI CTCAE v4.0 grading).

Herceptin SC, Perjeta IV, and docetaxel will be discontinued in any patient who develops clinical signs and symptoms suggesting symptomatic CHF with the diagnosis confirmed by a suggestive chest X-ray and a drop in LVEF by ECHO or MUGA. CHF should be treated and monitored according to standard medical practice.

Asymptomatic LVEF Decline (LVEF assessment scheduled in Appendix 1).

There are two parts.

Part 1: Patient management according to algorithm shown in Figure 4.

To ensure the safety of patients in the trial, Herceptin SC and Perjeta IV must be withheld in all patients for whom:

- **A decline of LVEF to** < **40%** is documented. If this value is confirmed within 3 weeks of the first assessment with use of the same assessment method, study drug must be discontinued.
- **LVEF decline to values 40–45% and** ≥ **10% points** below baseline, the decision to stop or continue study treatment is based on the algorithm shown in Figure 4

Part 2: Reporting in eCRF as per NCI CTCAE criteria v4.0 (see Appendix 4).

The intensity of asymptomatic LVEF decline described above will be reported on the basis of NCI CTCAE criteria v4.0 (“Ejection fraction decreased“).

The incidence of CHF will also be recorded throughout the study.

"Use of this document is governed by the terms of use on the first page of this document."

Figure 4 Asymptomatic Decline in Left Ventricular Ejection Fraction: Algorithm for Continuation and Discontinuation of Perjeta and Herceptin Based on Left Ventricular Ejection Fraction Assessments

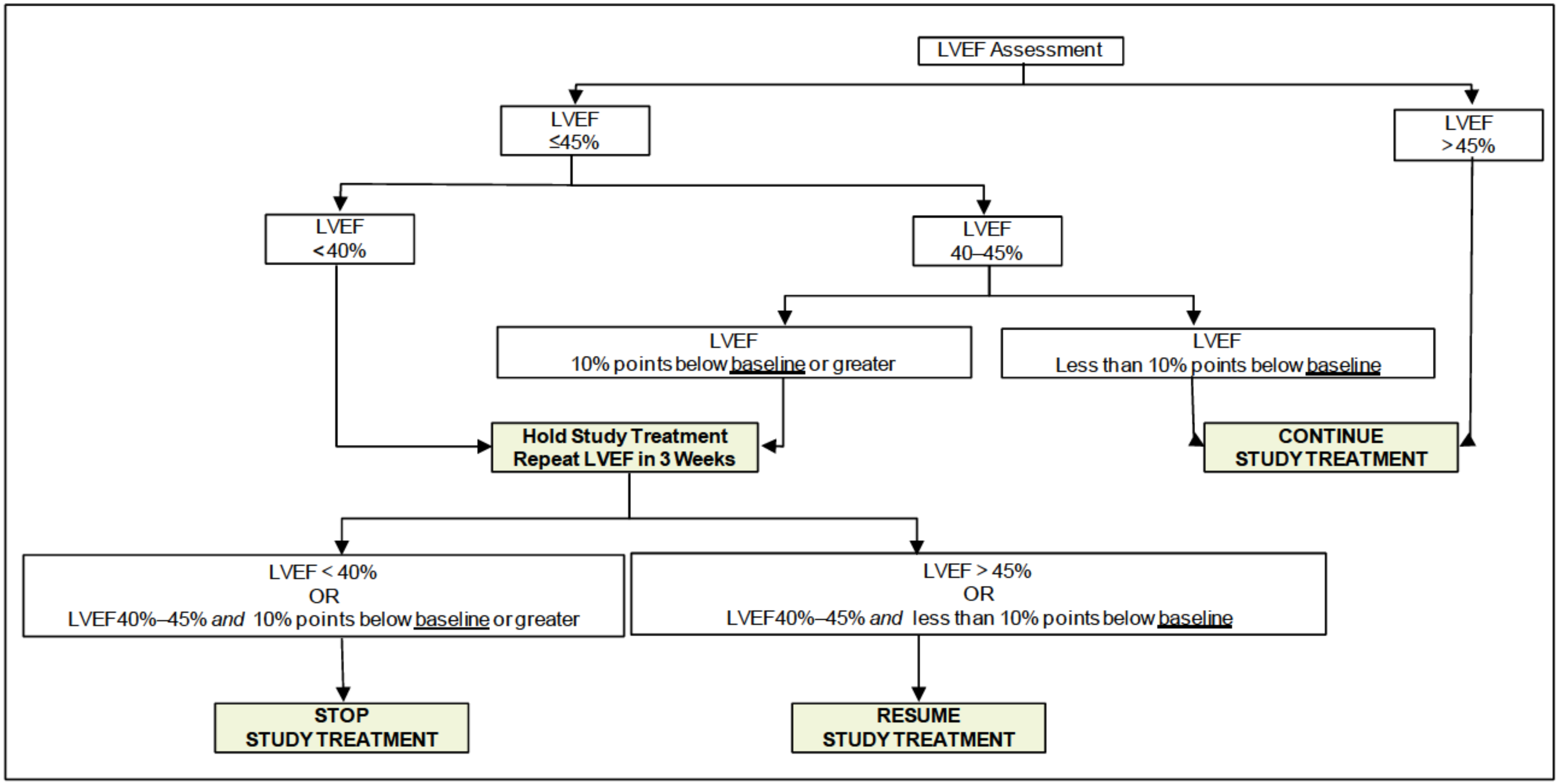

LVEF = left ventricular ejection fraction.

"Use of this document is governed by the terms of use on the first page of this document."

5.1.1.2 Administration-Related Reactions, Hypersensitivity Reactions, and Local-Site Reactions

Administration of monoclonal antibodies, including Herceptin SC and Perjeta IV, may cause ARRs. These include all adverse events leading to a systemic reaction following Herceptin SC, Perjeta IV, or docetaxel IV administration and which are considered related to the administration. Typically, adverse events such as chills and/or fever, dyspnea, hypotension, wheezing, bronchospasm, tachycardia, reduced oxygen saturation and respiratory distress, skin rashes, headache, nausea, or vomiting have been observed.

ARRs should be distinguished from local-site reactions, occurring after Herceptin SC injection or at the IV injection site of Perjeta or docetaxel. Local-site reactions are captured separately from ARRs.

ARRs may be clinically difficult to distinguish from hypersensitivity reactions.

Patients with extensive metastatic pulmonary disease involvement (e.g., lymphangitis, multiple metastases, and recurrent pleural effusions) and patients with preexisting pulmonary compromise who are treated with Herceptin may be at increased risk of severe or serious ARRs. Therefore, careful consideration must be made before enrolling patients with chronic pulmonary disease into the study.

Study treatment will be administered by staff trained to monitor for and respond to medical emergencies in a setting with emergency equipment.

Patients who experience the following events will be **discontinued** from the study treatment that is considered responsible for the event:

- Grade 4 hypersensitivity reaction,
- Acute respiratory distress syndrome (ARDS), or
- Bronchospasm

Patients who experience ARRs may be managed by:

- Slowing or stopping the Perjeta infusion for a Perjeta-related ARR
- Supportive care with oxygen, β-agonists, antihistamines, antipyretics, or corticosteroids as appropriate at the investigator's discretion

Premedication with corticosteroids, antihistamines, and antipyretics may be used before subsequent Herceptin SC or Perjeta infusions at the investigator's discretion.

In order to be able to calculate time to onset of such reactions, the occurrence of adverse events has to be documented with the **date** and **time** of the **onset** and duration of the event (i.e., **resolution** of the event).

"Use of this document is governed by the terms of use on the first page of this document."

If a subject experiences an ARR, the diagnosis should be used for the primary adverse event term (e.g., “infusion-related reaction,” “injection-site reaction,” or “anaphylactic reaction”).

The individual sign(s) and symptom(s) of the reaction, separated by local or systemic ones, should then be captured on forms dedicated for systemic injection reactions, systemic infusion reactions, local infusion-site reactions, and local injection-site reactions (see Section 5.3.5.1).

If a patient experiences a local and a systemic reaction following administration of a single dose of study drug, then two separate adverse events will need to be recorded.

Patients will be monitored until complete resolution of signs and symptoms of any systemic reactions.

5.1.1.3 Incomplete Dose or Dose Delay

Perjeta IV

Incomplete Loading Dose

In case the whole loading dose of Perjeta IV cannot be administered because of an ARR or other reason, the following guidelines apply:

- The patient should receive at **least 50% of the loading dose** in the first week. Therefore, if the patient receives $<$50% of the Cycle 1 dose, the patient should receive the remainder of the dose before Day 22, preferably within the first week. Thereafter, the patient should receive the usual maintenance dose 3 weeks after the first interrupted dose, as routinely scheduled. For example, if a patient received only 50% of the scheduled loading dose (i.e., only 420 mg instead of 840 mg of Perjeta IV), the patient should receive the remaining dose (420 mg of Perjeta IV), preferably in the first week, and then regular maintenance doses (420 mg of Perjeta IV) on Day 22, as routinely scheduled.
- If the patient receives **between 50% and 75% of** the loading dose, the patient should receive the remainder before Day 22, preferably within the first 2 weeks of Cycle 1. For example, if a patient received approximately only 60% of the scheduled loading dose, the patient should receive the remaining 40% within 2 weeks after the interrupted loading dose. Thereafter, the patient should receive the regular maintenance doses on Day 22, as routinely scheduled.
- If the patient receives $\geq$**75% of the loading dose**, additional loading is probably not necessary. However, the remainder of the loading dose may be given at the investigator's discretion. In such a case, the remainder may be given at any time before the next scheduled dose, or the patient may be given an additional loading dose on Day 22. If, after receiving an incomplete loading dose on Day 1, the patient cannot attend the site until Day 22, the patient should receive a second loading dose on Day 22. However, every effort should be made to give the remainder of the dose prior to Day 22.

"Use of this document is governed by the terms of use on the first page of this document."

Dose Delay

If a **dose is delayed** (i.e., the time between two sequential infusions is < **6 weeks**), the 420 mg dose of Perjeta IV should be administered as soon as possible. Do not wait until the next planned dose.

If a dose is missed (i.e., the time between two sequential infusions is ≥ **6 weeks**), a reloading dose of Perjeta IV (840 mg) should be given as described in the product labeling. If reloading is required for a given cycle, the three study therapies should be given on the same schedule as Cycle 1. Subsequent maintenance Perjeta IV doses of 420 mg will then be given every 3 weeks, starting 3 weeks later.

Herceptin SC

In the event a dose of Herceptin SC is incomplete, that dose will not be completed. Patients will receive the next scheduled dose per the Schedule of Assessments in Appendix 1.

No dose adjustment is needed in case of delayed administration of Herceptin SC as a fixed dose of Herceptin SC is used in this study.

5.1.1.4 Docetaxel Dose Modification for Toxicity

Docetaxel should be administered only under the supervision of a physician who is experienced in the use of cytotoxic cancer agents.

Significant hypersensitivity reactions can occur in patients who receive docetaxel, even after they receive adequate premedication. In the case of severe hypersensitivity reactions, docetaxel infusion should be discontinued immediately, symptomatic therapy should be initiated, and the patient should not be re-challenged with docetaxel. Localized skin erythema of the palms of the hands and soles of the feet with edema followed by desquamation has been observed with docetaxel.

Patients with severe fluid retention, such as pleural effusion, pericardial effusion, and ascites, should be monitored closely.

Dose reduction should occur in the case of development of severe peripheral neurotoxicity with docetaxel.

"Use of this document is governed by the terms of use on the first page of this document."

Table 2 Docetaxel Dose Adjustments

Docetaxel Dose	When
75 mg/m^2	Starting dose<br>Administer **only** if neutrophil count is $>$1500 $cells/mm^3$
100 mg/m^2	At the discretion of the treating physician, after at least 1 cycle of 75 mg/m^2 without any of the following toxicities:<br>• Febrile neutropenia<br>• Grade 4 neutropenia for $>$5 days<br>• ANC $<$100/µL for more than 1 day<br>• Other non-hematological toxicities of Grade $>$2 (NCI CTCAE, v4.0)
55 mg/m^2 (or 75 mg/m^2 if dose previously increased to 100 mg/m^2)	25% reduced dose in case of any of the following toxicities:<br>• Febrile neutropenia or neutrophils $<$500 $cells/mm^3$ for more than 1 week (after fully recovering to a neutrophil count $\geq$1500 $cells/mm^3$)<br>• Platelet count $<$100,000 $cells/mm^3$ (after recovering to a platelet count $\geq$100,000 $cells/mm^3$)<br>• Severe or cumulative cutaneous reactions
Permanently discontinue docetaxel	After any of the following toxicities:<br>• Severe hypersensitivity reactions<br>• Peripheral neuropathy $>$Grade 3<br>• Severe or cumulative cutaneous reactions that continue at a dose of 55 mg/m^2 without recovery<br>• Febrile neutropenia or neutrophils $<$500 $cells/mm^3$ without recovery<br>• Platelet $<$100,000 $cells/mm^3$ without recovery<br>• Total bilirubin $>$ULN without recovery<br>• Serum transaminase (AST/ALT) levels $>$1.5 $\times$ ULN concurrent with serum alkaline phosphatase levels $>$2.5 $\times$ ULN without recovery

NCI CTCAE = National Cancer Institute Common Terminology Criteria for Adverse Events; ULN = upper limit of normal.

Heart failure has been observed in patients who received docetaxel in combination with Herceptin. Cardiac function should be carefully monitored in patients who receive Herceptin with docetaxel. Details on monitoring of cardiac toxicity are given in Section 5.1.1.1).

Limited, non-comparative data from Phase I/II studies suggest that the combination of Perjeta and docetaxel may also result in myelosuppression. Given these data, it is expected that patients in this trial could experience hematologic adverse events while receiving treatment. For this reason, all patients will be monitored for hematologic events, and dose reductions of docetaxel with or without growth factor (e.g., G-CSF) support will be allowed in this protocol.

"Use of this document is governed by the terms of use on the first page of this document."

Warning and precautions for docetaxel IV: The IV chemotherapy drug docetaxel contains 50 vol % ethanol (alcohol) (i.e., up to 0.395 g [0.5 mL] per vial), equivalent to 10 mL of beer or 4 mL wine per vial (Taxotere EPAR product information update 16 Jun 2014; available at www.ema.europa.eu/ema).

- Harmful for those suffering from alcoholism
- To be taken into account in pregnant or breastfeeding women, children, and high-risk groups such as patients with liver disease, or epilepsy
- The amount of alcohol in this medicinal product may alter the effects of other medicinal products.
- The amount of alcohol in this medicinal product may impair the patient's ability to drive or use machines.

For further information, refer to the local prescribing information for docetaxel.

5.1.2 Warning and Precautions for Herceptin SC

Herceptin SC therapy should be initiated only under supervision of a physician who is experienced in the treatment of patients with cancer.

Serious adverse reactions, including cardiac dysfunctions, ARRs, hypersensitivity, allergic-like reactions, and pulmonary events, have been observed in patients receiving Herceptin therapy. For some patients, symptoms progressively worsened and led to further pulmonary complications. Initial improvement followed by clinical deterioration and delayed reactions with rapid clinical deterioration have also been reported.

Fatalities have occurred within hours and up to 1 week following Herceptin IV administration. On very rare occasions, patients have experienced the onset of administration-related symptoms or pulmonary symptoms more than 6 hours after the start of the Herceptin administration. Patients should be warned of the possibility of such a late onset and should be instructed to contact their physician if these symptoms occur. Patients who have dyspnea at rest due to comorbidities may be at increased risk of a fatal ARR.

5.1.2.1 Administration-Related Reactions, Allergic-Like Reactions, and Hypersensitivity

Serious adverse reactions to Herceptin IV that have been reported infrequently include dyspnea, hypotension, wheezing, bronchospasm, asthma, tachycardia, reduced oxygen saturation, anaphylaxis, respiratory distress, urticaria, and angioedema. Although such events were not reported in the clinical trial with Herceptin SC, caution should be exercised as these events have been associated with the IV formulation.

These reactions were usually associated with the first administration of Herceptin and generally occurred during or immediately following administration.

"Use of this document is governed by the terms of use on the first page of this document."

Patients should be observed for administration-related reactions for 6 hours after the first injection of Herceptin SC and for 2 hours after subsequent injections.

In the pivotal study BO22227, there was a higher rate of Herceptin SC injection-site reactions compared with the Herceptin IV infusion (11.1% in Herceptin SC vs. 0.3% in Herceptin IV). With few exceptions, all of these events were of Grade 1 intensity.

ARRs were more commonly observed in patients who received Herceptin SC (47.8% in Herceptin SC vs. 37.2% in Herceptin IV). Erythema and cough were the primary adverse events responsible for the observed difference. The large majority of events (97%) were of Grade 1 or 2 intensity.

Serious reactions to Herceptin IV have been treated successfully with supportive therapy, such as oxygen, β-agonists, and corticosteroids.

5.1.2.2 Pulmonary Events

Caution is recommended with use of the Herceptin SC formulation as severe pulmonary events have been reported with the use of the Herceptin IV formulation in the post-marketing setting. These events have occasionally been fatal. They may occur as part of an ARR or with delayed onset. In addition, cases of interstitial lung disease, including lung infiltrates, ARDS, pneumonia, pneumonitis, pleural effusion, respiratory distress, acute pulmonary edema, and respiratory insufficiency, have been reported with Herceptin IV. These have been most common with the first infusion, and their severity has decreased with subsequent infusions. Serious reactions have been treated successfully with supportive therapy, such as oxygen, β-agonists, and corticosteroids. ARDS has been reported with a fatal outcome.

5.1.2.3 Cardiac Dysfunction

Heart failure (NYHA Class II–IV) has been observed in patients who have received Herceptin therapy alone or in combination with docetaxel following anthracycline (doxorubicin or epirubicin)–containing chemotherapy. This may be moderate to severe and has been associated with death.

Risk factors for Herceptin-associated cardiac dysfunction include increased age, concomitant administration with anthracyclines, and declining LVEF while on Herceptin treatment. If symptomatic cardiac failure develops during Herceptin therapy, it should be treated with the standard medications for this purpose.

Because the half-life of Herceptin is approximately 28–38 days, Herceptin may persist in the circulation for up to 27 weeks after Herceptin treatment is stopped. Patients who receive anthracyclines after stopping Herceptin may possibly be at increased risk of cardiac dysfunction. If possible, physicians should avoid anthracycline-based therapy for up to 27 weeks after stopping Herceptin. If anthracyclines are used, the patient's cardiac function should be monitored carefully.

"Use of this document is governed by the terms of use on the first page of this document."

Most patients who developed heart failure in the Phase III trials of Herceptin in MBC improved with standard medical treatment. This treatment included diuretics, cardiac glycosides, and/or angiotensin-converting enzyme inhibitors. The majority of patients with cardiac symptoms and evidence of a clinical benefit of Herceptin treatment continued on weekly therapy with Herceptin without experiencing additional clinical cardiac events.

5.1.3 Warning and Precautions for Perjeta

Perjeta therapy should be initiated only under supervision of a physician who is experienced in the treatment of patients with cancer.

5.1.3.1 Risk of Hypersensitivity Reactions, Including Anaphylaxis and Administration-Related Symptoms

ARRs typically occur during or shortly after the administration of monoclonal antibodies but may also show a delayed onset. The true relation of an event to administration of study treatment is therefore difficult to ascertain, particularly when treatment regimens involve combination therapy.

In general, antibody administration-related adverse events are more frequent and severe with the first infusion and decrease in number and severity over time. The majority of adverse events resolve fully.

Perjeta will be administered by staff trained to monitor for and respond to medical emergencies in a setting with emergency equipment. Patients will be monitored during each Perjeta infusion and for 30–60 minutes (60 minutes for the initial administration) following the completion of the infusion for any adverse effects. If administration-related symptoms occur, patients will be monitored until complete resolution of signs and symptoms. Patients who experience administration-related symptoms may subsequently be premedicated with acetaminophen, diphenhydramine, or meperidine.

Infusion of Perjeta should be stopped in patients who develop dyspnea or clinically significant hypotension (defined per investigator discretion). Patients who experience a Grade 3 or 4 hypersensitivity reaction or ARDS should not receive additional Perjeta.

Refer to the Perjeta Investigator's Brochure for the most recent data related to the risk of hypersensitivity reactions.

5.1.3.2 Risk of Cardiac Dysfunction

Like Herceptin, Perjeta is directed at the HER2 receptor and may be associated with a risk of cardiac dysfunction.

Patients with significant cardiac disease or baseline LVEF $<50\%$ are not eligible for this study.

"Use of this document is governed by the terms of use on the first page of this document."

In the pivotal Phase III study CLEOPATRA in patients with MBC, Perjeta in combination with Herceptin and docetaxel was not associated with an increase in the incidence of symptomatic LVSD (CHF) or decreases in LVEF compared with placebo in combination with Herceptin and docetaxel. However, patients who have received prior anthracyclines or prior radiotherapy to the chest area may be at higher risk of decreased LVEF.

In the neoadjuvant setting (NEOSPHERE), the incidence of LVSD was higher in patients treated with Perjeta than in patients treated with Herceptin and docetaxel. An increased incidence of LVEF decline was observed in patients treated with Perjeta in combination with Herceptin and docetaxel; LVEF recovered to $\geq$50% in all patients during the treatment period.

Perjeta has not been studied in patients with a pretreatment LVEF value of $<$50%, a prior history of CHF, decreases in LVEF to $<$50% during prior Herceptin adjuvant therapy, conditions that could impair left ventricular function such as uncontrolled hypertension, recent myocardial infarction, serious cardiac arrhythmia requiring treatment, or a cumulative prior anthracycline exposure to $>$360 mg/m^2 of doxorubicin or its equivalent.

Risk factors for Perjeta-associated cardiac dysfunction should be carefully weighed against the potential benefit in patients who have received prior anthracyclines. During the screening/baseline period, complete medical history information will be collected from all patients to explore possible risk factors for treatment-associated CHF.

Monitoring of LVEF is required while patients are receiving study treatment.
If symptomatic left ventricular dysfunction develops (NCI CTCAE v4.0 Grade 3 or 4) with a drop in LVEF consistent with cardiac failure, the patient must discontinue study treatment. Left ventricular dysfunction, whether symptomatic or not, should be treated and followed according to standard medical practice.

The Perjeta Investigator's Brochure should be referred to for most recent data relating to risk of cardiac dysfunction.

5.1.3.3 Risk of EGFR-Related Toxicities

Although Perjeta targets HER2 because of its role in heterodimerization with other members of the HER family (e.g., EGFR), it may cause toxicities associated with the use of EGFR TK inhibitors. In the 7-week IV and 26-week toxicity studies in cynomolgus monkeys, there were treatment-related increases in the incidence of diarrhea.

Diarrhea has been observed in patients who are treated with Perjeta in Phase II single-agent studies and in combination therapy studies. For patients who experience diarrhea, early intervention with loperamide should be considered.

Rash has also been observed with EGFR TK inhibitors.

"Use of this document is governed by the terms of use on the first page of this document."

The Perjeta Investigator's Brochure should be referred to for most recent data relating to the risk of EGFR-related toxicities.

5.1.4 Pregnancy

ICH M3 Guidance requires precautions to be taken to minimize risk to fetus or embryo when including WOCBP. This includes the use of highly effective contraceptive measures, excluding pregnancy at baseline (serum test), continued pregnancy monitoring, and continued pregnancy testing up to 7 months following last dose of study drug (follow-up period based on pharmacokinetic considerations).

Reproductive toxicity was identified during preclinical studies; both Herceptin and Perjeta administered to pregnant cynomolgus monkeys during organogenesis led to oligohydramnios, delayed renal development, and embryo-fetal deaths. There are no clinical studies of Herceptin or Perjeta in pregnant women. IgGs are known to cross the placental barrier. Therefore, neither Perjeta nor Herceptin should be used during pregnancy.

WOCBP (who have not undergone surgical sterilization) must agree to use a highly effective non-hormonal form of contraception or two effective forms of non-hormonal contraception by the patient and/or partner.

Methods of birth control that result in a low failure rate (i.e., less than 1% per year) when used consistently and correctly are considered **highly effective forms** of contraception. The following non-hormonal methods of contraception are acceptable:

- True abstinence when this is in line with the preferred and usual lifestyle of the patient. (Periodic abstinence [e.g., calendar, ovulation, symptothermal postovulation methods] and withdrawal are not acceptable methods of contraception.)
- Male sterilization (with appropriate postvasectomy documentation of the absence of sperm in the ejaculate). For female patients, the vasectomized male partner should be the sole partner.

Or two of the following effective forms of contraception:

- Placement of intrauterine device (IUD) or intrauterine system (IUS). Consideration should be given to the type of device being used as there are higher failure rates quoted for certain types (e.g., steel or copper wire). The risks (in terms of potential stimulation of hormone-responsive breast cancer by systemically absorbed hormones) and benefits (effective contraception) of hormone-releasing IUDs/IUSs should also be carefully considered for individual patients.
- Condom with spermicidal foam/gel/film/cream/suppository
- Occlusive cap (diaphragm or cervical/vault caps) with spermicidal foam/gel/film/cream/suppository

"Use of this document is governed by the terms of use on the first page of this document."

The use of barrier contraceptives should always be supplemented with the use of a spermicide. The following should be noted:

- Failure rates indicate that, when used alone, the diaphragm and condom are not highly effective forms of contraception. Therefore, the use of additional spermicides does confer additional theoretical contraceptive protection.
- However, spermicides alone are ineffective at preventing pregnancy when the whole ejaculate is spilled. Therefore, spermicides are not a barrier method of contraception and should not be used alone.

It should be noted that **two forms of effective contraception are required**. A double-barrier method is acceptable, which is defined as condom and occlusive cap (diaphragm or cervical/vault caps) with spermicidal foam/gel/film/cream/suppository.

Postmenopausal is defined as $\geq$12 months of amenorrhea (see details in Section 4.5.2).

On the basis of pharmacokinetic considerations, contraception must continue for the duration of study treatment and for **at least 7 months** after the last dose of study treatment.

It is not known whether Herceptin or Perjeta is excreted in human milk. As maternal IgG is excreted in milk and either monoclonal antibody could harm infant growth and development, women should be advised to discontinue nursing during Perjeta or Herceptin therapy and not to breastfeed for at least 7 months following the last dose of either monoclonal antibody.

5.2 SAFETY PARAMETERS AND DEFINITIONS

Safety assessments will consist of monitoring and recording adverse events including serious adverse events and non-serious adverse events that are immediately reportable, performing of protocol-specified safety laboratory assessments, measuring protocol-specified vital signs, and conducting other protocol-specified tests that are deemed critical to the safety evaluation of the study.

Certain types of events require immediate reporting to the Sponsor, as outlined in Section 5.4.

5.2.1 Adverse Events

According to the ICH guideline for Good Clinical Practice, an adverse event is any untoward medical occurrence in a clinical investigation subject administered a pharmaceutical product, regardless of causal attribution. An adverse event can therefore be any of the following:

- Any unfavorable and unintended sign (including an abnormal laboratory finding), symptom, or disease temporally associated with the use of a medicinal product, whether or not considered related to the medicinal product

"Use of this document is governed by the terms of use on the first page of this document."

- Any new disease or exacerbation of an existing disease (a worsening in the character, frequency, or severity of a known condition), except for preexisting medical condition as described in Section 5.3.5.9
- Recurrence of an intermittent medical condition (e.g., headache) not present at baseline
- Any deterioration in a laboratory value or other clinical test (e.g., ECG, X-ray) that is associated with symptoms or leads to a change in study treatment or concomitant treatment or discontinuation from study drug
- Adverse events that are related to a protocol-mandated intervention including those that occur prior to assignment of study treatment (e.g., screening invasive procedures such as biopsies)

5.2.2 Serious Adverse Events (Immediately Reportable to the Sponsor)

A serious adverse event is any adverse event that meets any of the following criteria:

- Is fatal (i.e., the adverse event actually causes or leads to death)
- Is life-threatening (i.e., the adverse event, in the view of the investigator, places the patient at immediate risk of death)

  This does not include any adverse event that had it occurred in a more severe form or was allowed to continue might have caused death.
- Requires or prolongs inpatient hospitalization (for details see Section 5.3.5.11)
- Results in persistent or significant disability/incapacity (i.e., the adverse event results in substantial disruption of the patient's ability to conduct normal life functions)
- Is a congenital anomaly/birth defect in a neonate/infant born to a mother exposed to study drug
- Is a significant medical event in the investigator's judgment (e.g., may jeopardize the patient or may require medical/surgical intervention to prevent one of the outcomes listed above)

The terms "severe" and "serious" are not synonymous. Severity refers to the intensity of an adverse event (rated as mild, moderate, severe, life-threatening or fatal according to NCI CTCAE v4.0 criteria; see Section 5.3.3); the event itself may be of relatively minor medical significance (such as severe headache without any further findings).

Severity and seriousness need to be independently assessed for each adverse event recorded on the eCRF.

Serious adverse events are required to be reported by the investigator to the Sponsor within 24 hours after learning of the event (see Section 5.4.2 for reporting instructions).

"Use of this document is governed by the terms of use on the first page of this document."

5.2.3 Non-Serious Adverse Events (Immediately Reportable to the Sponsor)

Non-serious adverse events that are immediately reportable are required to be reported by the investigator to the Sponsor within 24 hours after learning of the event (see Section 5.4.2 for reporting instructions).

For this study, this applies for the following adverse events:

- CHF
- An asymptomatic decline in LVEF that requires treatment or that leads to discontinuation of study treatment
- Cases of potential drug-induced liver injury that include an elevated ALT or AST in combination with either an elevated bilirubin or clinical jaundice, as defined by Hy's law (see Section 5.3.5.7)
- Suspected transmission of an infectious agent by the study drug
- Any organism, virus, or infectious particle (e.g., prion protein transmitting transmissible spongiform encephalopathy), pathogenic or non-pathogenic, is considered an infectious agent. A transmission of an infectious agent may be suspected from clinical symptoms or laboratory findings that indicate an infection in a patient exposed to a medicinal product. This term applies only when a contamination of the study drug is suspected.

Note: In general, asymptomatic declines in LVEF should not be reported as adverse events since LVEF data are collected separately in the eCRF. Exceptions to this rule are as follows:

- An asymptomatic decline in LVEF to a value of 10 percentage points below baseline or lower and $<$50% must be reported as an adverse event
- An asymptomatic decline in LVEF that requires treatment or that leads to discontinuation of study treatment must be reported in an expedited manner with use of the Serious Adverse Event form and classifying the event as a Non-Serious Event that is Immediately Reportable

For reporting in eCRF: Both cases should be reported as "Ejection fraction decreased" and graded according to NCI CTCAE v4.0.

5.3 METHODS AND TIMING FOR CAPTURING AND ASSESSING SAFETY PARAMETERS

The investigator is responsible for ensuring that all adverse events (see Section 5.2.1 for definition) are recorded on the Adverse Event eCRF and reported to the Sponsor in accordance with instructions provided in this section and in Sections 5.4, 5.5, and 5.6.

For each adverse event recorded on the Adverse Event eCRF, the investigator will make an assessment of seriousness (see Section 5.2.2 for seriousness criteria), severity (see Section 5.3.3), and causality (see Section 5.3.4).

"Use of this document is governed by the terms of use on the first page of this document."

For each local-site reactions and systemic ARR recorded on the corresponding dedicated eCRF, the **date** and **time of onset** and the **date and time of resolution** has to be recorded (see Sections 5.1.1.2 and 5.3.5.1).

5.3.1 Adverse Event Reporting Period

Investigators will seek information on adverse events at each patient contact. All adverse events, whether reported by the patient or noted by study personnel, will be recorded in the patient's medical record and on the Adverse Event eCRF.

After informed consent has been obtained **but prior to initiation of study drug**, only serious adverse events considered to be related to a protocol-mandated intervention should be reported (e.g., serious adverse events related to invasive procedures such as biopsies).

After initiation of study drug, all adverse events will be reported until the post-treatment safety follow-up visit (i.e., approximately 28 days after the last dose of study drug).

Adverse events that are ongoing at the time of the post-treatment follow-up visit should be followed depending on the event type:

- All cardiac adverse events (regardless of seriousness or causality) and all serious adverse events (regardless of causality) should be followed until resolution/stabilization/death up to 1 year after the last dose or end of the study, whichever comes first.
- Non-cardiac, non-serious adverse events (regardless of causality) should be followed **only** until the post-treatment follow-up visit.

Only the following new adverse events that start after the post-treatment follow-up visit should be reported to the clinical database:

- Cardiac events (regardless of causality or seriousness) that start up to 2 years after the last dose should be reported. These events should be followed until resolution/stabilization/death up to 2 years after the start of the event or end of the study, whichever comes first.
- Herceptin SC and Perjeta IV-related serious adverse events should be reported at any time regardless of the start date. These events should be followed until resolution/stabilization/death up to 1 year after the start of the event.

After the end of the study, the investigator should report any serious adverse events that are believed to be related to study drug treatment (see Section 5.6).

"Use of this document is governed by the terms of use on the first page of this document."

5.3.2 Eliciting Adverse Event Information

A consistent methodology of non-directive questioning should be adopted for eliciting adverse event information at all patient evaluation timepoints. Examples of non-directive questions include the following:

"How have you felt since your last clinic visit?"

"Have you had any new or changed health problems since you were last here?"

5.3.3 Assessment of Severity of Adverse Events

The adverse event severity grading scale for the NCI CTCAE v4.0 will be used for assessing adverse event severity. The guidelines in Table 3 will be used to assess ONLY the severity for adverse events that are not specifically listed in the NCI CTCAE.

Table 3 Adverse Event Severity Grading Scale

Grade	Severity
1	Mild; asymptomatic or mild symptoms; clinical or diagnostic observations only; or intervention not indicated
2	Moderate; minimal, local, or non-invasive intervention indicated; or limiting age-appropriate instrumental activities of daily living [a]
3	Severe or medically significant, but not immediately life-threatening; hospitalization or prolongation of hospitalization indicated; disabling; or limiting self-care activities of daily living [b, c]
4	Life-threatening consequences or urgent intervention indicated [d]
5	Death related to adverse event [d]

NCI CTCAE = National Cancer Institute Common Terminology Criteria for Adverse Events.

Note: Based on the NCI CTCAE (version 4.0), which can be found at: http://evs.nci.nih.gov/ftp1/CTCAE/CTCAE_4.03_2010-06-14_QuickReference_8.5x11.pdf

[a] Instrumental activities of daily living refer to preparing meals, shopping for groceries or clothes, using the telephone, managing money, etc.

[b] Examples of self-care activities of daily living include bathing, dressing and undressing, feeding one's self, using the toilet, and taking medications, as performed by patients who are not bedridden.

[c] If an event is assessed as a "significant medical event," it must also be reported as a serious adverse event (see Section 5.4.2 for reporting instructions), per the definition of serious adverse event in Section 5.2.2.

[d] Grade 4 and 5 events must also be reported as serious adverse events (see Section 5.4.2 for reporting instructions), per the definition of serious adverse event in Section 5.2.2.

"Use of this document is governed by the terms of use on the first page of this document."

5.3.4 Assessment of Causality of Adverse Events

Investigators should use their knowledge of the patient, the circumstances surrounding the event, and an evaluation of any potential alternative causes to determine whether or not an adverse event is considered to be related to the study drug, indicating "yes" or "no" accordingly. The following guidance should be taken into consideration:

- Temporal relationship of event onset to the initiation of study drug
- Course of the event, considering especially the effects of dose reduction, discontinuation of study drug, or re-introduction of study drug (where applicable)
- Known association of the event with the study drug or with similar treatments
- Known association of the event with the disease under study
- Presence of risk factors in the patient or use of concomitant medications known to increase the occurrence of the event
- Presence of non–treatment-related factors that are known to be associated with the occurrence of the event

For patients who receive combination therapy, causality will be assessed individually for each protocol-mandated therapy.

5.3.5 Procedures for Recording Adverse Events

Investigators should use correct medical terminology/concepts when recording adverse events on the Adverse Event eCRF. Avoid colloquialisms and abbreviations.

Only one adverse event term should be recorded in the event field on the Adverse Event eCRF.

5.3.5.1 Administration-Related Reactions and Local Injection-Site Reactions

Adverse events that occur during or within 24 hours after study drug administration and are judged to be related to study drug infusion or injection should be captured as a diagnosis (e.g., “infusion-related reaction,” “injection-site reaction,” “anaphylactic reaction”) on the Adverse Event eCRF. If possible, avoid ambiguous terms such as “systemic reaction.”

Associated signs and symptoms should be recorded on the dedicated Administration-Related Reaction eCRF. If a patient experiences both a local and systemic reaction to the same dose of study drug, each reaction should be recorded separately on the Adverse Event eCRF with signs and symptoms also recorded separately on the dedicated Administration-Related Reaction eCRF (see Section 5.1.1.2).

5.3.5.2 Diagnosis versus Signs and Symptoms

For adverse events other than ARRs (see Section 5.3.5.1), a final diagnosis (if known) should be recorded on the Adverse Event eCRF rather than individual signs and

"Use of this document is governed by the terms of use on the first page of this document."

symptoms (e.g., record only liver failure or hepatitis rather than jaundice, asterixis, and elevated transaminases). However, if a constellation of signs and/or symptoms cannot be medically characterized as a single diagnosis or syndrome at the time of reporting, each individual event should be recorded on the Adverse Event eCRF. If a diagnosis is subsequently established, all previously reported adverse events based on signs and symptoms should be nullified and replaced by adverse event report based on the single diagnosis with a starting date that corresponds to the starting date of the first symptom of the eventual diagnosis.

5.3.5.3 Adverse Events Occurring Secondary to Other Events

In general, adverse events occurring secondary to other events (e.g., cascade events or clinical sequelae) should be identified by their primary cause, with the exception of severe or serious secondary events. However, medically significant adverse events occurring secondary to an initiating event that are separated in time should be recorded as independent events on the Adverse Event eCRF. For example:

- If vomiting results in mild dehydration with no additional treatment in a healthy adult, only vomiting should be reported on the eCRF.
- If vomiting results in severe dehydration, both events should be reported separately on the eCRF.
- If a severe gastrointestinal hemorrhage leads to renal failure, both events should be reported separately on the eCRF.
- If dizziness leads to a fall and subsequent fracture, all three events should be reported separately on the eCRF.
- If neutropenia is accompanied by a mild, non-serious infection, only neutropenia should be reported on the eCRF.
- If neutropenia is accompanied by a severe or serious infection, both events should be reported separately on the eCRF.

All adverse events should be recorded separately on the Adverse Event eCRF if it is unclear as to whether the events are associated.

5.3.5.4 Persistent or Recurrent Adverse Events

A persistent adverse event is one that extends continually, without resolution, between patient evaluation timepoints. Such events should be recorded only once on the Adverse Event eCRF. The initial severity (intensity or grade) of the event should be recorded, and the severity should be updated to reflect the most extreme severity any time the event worsens. If the event becomes serious, the Adverse Event eCRF should be updated to reflect this.

A recurrent adverse event is one that resolves between patient evaluation timepoints and subsequently recurs. Each recurrence of an adverse event should be recorded separately on the Adverse Event eCRF.

"Use of this document is governed by the terms of use on the first page of this document."

5.3.5.5 Abnormal Laboratory Values

Not every laboratory abnormality qualifies as an adverse event. A laboratory test result must be reported as an adverse event if it meets any of the following criteria:

- Is accompanied by clinical symptoms
- Results in a change in study treatment (e.g., dosage modification, treatment interruption, or treatment discontinuation)
- Results in a medical intervention (e.g., potassium supplementation for hypokalemia) or a change in concomitant therapy
- Is clinically significant in the investigator's judgment

It is the investigator's responsibility to review all laboratory findings. Medical and scientific judgment should be exercised in deciding whether an isolated laboratory abnormality should be classified as an adverse event.

If a clinically significant laboratory abnormality is a sign of a disease or syndrome (e.g., alkaline phosphatase and bilirubin 5 × ULN associated with cholestasis), only the diagnosis (i.e., cholestasis) should be recorded on the Adverse Event eCRF.

If a clinically significant laboratory abnormality is not a sign of a disease or syndrome, the abnormality itself should be recorded on the Adverse Event eCRF along with a descriptor indicating if the test result is above or below the normal range (e.g., "elevated potassium" as opposed to "abnormal potassium"). If the laboratory abnormality can be characterized by a precise clinical term per standard definitions, the clinical term should be recorded as the adverse event. For example, an elevated serum potassium level of 7.0 mEq/L should be recorded as "hyperkalemia."

Observations of the same clinically significant laboratory abnormality from visit to visit should be recorded only once on the Adverse Event eCRF, (see Section 5.3.5.4 for details on recording persistent adverse events).

Follow-up of Abnormal Laboratory Test Values

In the event of medically significant unexplained abnormal laboratory test values, the tests should be repeated and followed up until they have returned to the normal range and/or an adequate explanation of the abnormality is found. If a clear explanation is established, it should be recorded on the eCRF.

5.3.5.6 Abnormal Vital Sign Values

Not every vital sign abnormality qualifies as an adverse event. A vital sign result should be reported as an adverse event if it meets any of the following criteria:

- Is accompanied by clinical symptoms
- Results in a change in study treatment (e.g., dosage modification, treatment interruption, or treatment discontinuation)
- Results in a medical intervention or a change in concomitant therapy
- Is clinically significant in the investigator's judgment

"Use of this document is governed by the terms of use on the first page of this document."

It is the investigator's responsibility to review all vital sign findings. Medical and scientific judgment should be exercised in deciding whether an isolated vital sign abnormality should be classified as an adverse event.

If a clinically significant vital sign abnormality is a sign of a disease or syndrome (e.g., high blood pressure), only the diagnosis (i.e., hypertension) should be recorded on the Adverse Event eCRF.

Observations of the same clinically significant vital sign abnormality from visit to visit should be recorded only once on the Adverse Event eCRF, (see Section 5.3.5.4 for details on recording persistent adverse events).

5.3.5.7 Abnormal Liver Function Tests

The finding of an elevated ALT or AST ($>3\times$ baseline value) in combination with either an elevated total bilirubin ($>2\times$ ULN) or clinical jaundice in the absence of cholestasis or other causes of hyperbilirubinemia is considered to be an indicator of severe liver injury. Therefore, investigators must report as an adverse event the occurrence of either of the following:

- Treatment-emergent ALT or AST $>3\times$ baseline value in combination with total bilirubin $>2\times$ ULN (of which $\geq$35% is direct bilirubin).
- Treatment-emergent ALT or AST $>3\times$ baseline value in combination with clinical jaundice.

The most appropriate diagnosis or (if a diagnosis cannot be established) the abnormal laboratory values should be recorded on the Serious Adverse Event eCRF (see Section 5.3.5.2) and reported to the Sponsor immediately (i.e., no more than 24 hours after learning of the event), either as a serious adverse event or a non-serious adverse event of special interest (see Section 5.4.2).

5.3.5.8 Deaths

For this protocol, mortality is an efficacy endpoint. Deaths that occur during the protocol-specified adverse event reporting period (see Section 5.3.1) that are attributed by the investigator solely to progression of the underlying disease of metastatic breast cancer should be recorded only on the Study Completion/Early Discontinuation eCRF. All other on-study deaths, regardless of relationship to study drug, must be recorded on the Adverse Event eCRF and immediately reported to the Sponsor (see Section 5.4.2). An internal monitoring committee will monitor the frequency of deaths from all causes.

Death should be considered an outcome and not a distinct event. The event or condition that caused or contributed to the fatal outcome should be recorded as the single medical concept on the Adverse Event eCRF. Generally, **only one** such event should be reported.

"Use of this document is governed by the terms of use on the first page of this document."

The term **“sudden death”** should only be used for the occurrence of an abrupt and unexpected death due to presumed cardiac causes in a patient with or without preexisting heart disease, within 1 hour of the onset of acute symptoms or, in the case of an unwitnessed death, within 24 hours after the patient was last seen alive and stable.

If the cause of death is unknown and cannot be ascertained at the time of reporting, **“unexplained death”** should be recorded on the Adverse Event eCRF. If the cause of death later becomes available (e.g., after autopsy), “unexplained death” should be replaced by the established cause of death.

During survival follow-up, deaths attributed to new progression of disease should be recorded only on the Study Completion/Early Discontinuation eCRF page.

5.3.5.9 Preexisting Medical Conditions

A preexisting medical condition is one that is present at the screening visit for this study. Such conditions should be recorded on the General Medical History and Baseline Conditions eCRF.

A preexisting medical condition should be recorded as an adverse event only if the frequency, severity, or character of the condition worsens during the study.

When recording such events on the Adverse Event eCRF, it is important to convey the concept that the preexisting condition has changed by including applicable descriptors (e.g., “more frequent headaches”).

5.3.5.10 Lack of Efficacy or Worsening of Breast Cancer

Events that are clearly consistent with the expected pattern of progression of the underlying disease should not be recorded as adverse events. These data will be captured as efficacy assessment data only. In most cases, the determination of progression will be based on RECIST v1.1 (see Appendix 5). In rare cases, the determination of clinical progression will be based on symptomatic deterioration.

However, every effort should be made to document progression through use of objective criteria. If there is any uncertainty as to whether an event is due to disease progression, it should be reported as an adverse event.

5.3.5.11 Hospitalization or Prolonged Hospitalization

Any adverse event that results in hospitalization or prolonged hospitalization should be documented and reported as a serious adverse event (per the definition of serious adverse event in Section 5.2.2), except as outlined below.

The following hospitalization scenarios are not considered to be serious adverse events:

- Hospitalization for respite care

"Use of this document is governed by the terms of use on the first page of this document."

- Planned hospitalization required by the protocol (e.g., for study drug administration or insertion of access device for study drug administration)
- Hospitalization for a preexisting condition, provided that all of the following criteria are met:

  The hospitalization was planned prior to the study or was scheduled during the study when elective surgery became necessary because of the expected normal progression of the disease.

  The patient has not suffered an adverse event.
- Hospitalization due solely to progression of the underlying cancer

5.3.5.12 Adverse Events Associated with an Overdose or Error in Drug Administration

Study drug overdose is the accidental or intentional use of the drug in an amount higher than the dose being studied. An overdose or incorrect administration of study drug is not an adverse event unless it results in untoward medical effects.

Any study drug overdose or incorrect administration of study drug should be noted on the Study Drug Administration eCRF.

All adverse events associated with an overdose or incorrect administration of study drug should be recorded on the Adverse Event eCRF. If the associated adverse event fulfills seriousness criteria, the event should be reported to the Sponsor within 24 hours after learning of the event (see Section 5.4.2).

5.3.5.13 Adverse Events in Individuals Not Enrolled in the Study

If an adverse event inadvertently occurs in an individual not enrolled in the study (e.g., during administration of study drug), the Adverse Event Form provided to investigators should be completed and submitted to Roche or its designee, either by faxing or by scanning and emailing the form with use of the fax number or email address provided to investigators.

5.4 IMMEDIATE REPORTING REQUIREMENTS FROM INVESTIGATOR TO SPONSOR

The investigator must report the following events to the Sponsor within 24 hours after learning of the event, regardless of relationship to study drug:

- Serious adverse events
- Non-serious adverse events that are immediately reportable as defined in Section 5.2.3
- Pregnancies

"Use of this document is governed by the terms of use on the first page of this document."

The investigator must report new significant follow-up information for these events to the Sponsor within 24 hours after becoming aware of the information. New significant information includes the following:

- New signs or symptoms or a change in the diagnosis
- Significant new diagnostic test results
- Change in causality based on new information
- Change in the event's outcome, including recovery
- Additional narrative information on the clinical course of the event

Investigators must also comply with local requirements for reporting serious adverse events to the local health authority and IRB or IEC.

5.4.1 Emergency Medical Contacts

To ensure the safety of study patients, an Emergency Medical Call Center Help Desk will access the Sponsor Medical Emergency List, escalate emergency medical calls, provide medical translation service (if necessary), connect the investigator with a Sponsor Medical Monitor, and track all calls. The Emergency Medical Call Center Help Desk will be available 24 hours per day, 7 days per week. Toll-free numbers for the Help Desk as well as Medical Monitor contact information will be distributed to all investigators.

5.4.2 Reporting Requirements for Serious Adverse Events and Non-Serious Adverse Events that are Immediately Reportable

For reports of serious adverse events and non-serious adverse events that are immediately reportable, investigators should record all case details that can be gathered within 24 hours on the Adverse Event eCRF and submit the report via the electronic data capture (EDC) system. A report will be generated and sent to the Sponsor Safety Risk Management by the EDC system.

In the event that the EDC system is unavailable, a paper Serious Adverse Event/Non-Serious Adverse Event that is Immediately Reportable CRF and fax cover sheet should be completed and submitted to Roche or its designee immediately (i.e., no more than 24 hours after learning of the event), either by faxing or by scanning and emailing the form with use of the fax number or email address provided to investigators. Once the EDC system is available, all information will need to be entered and submitted via the EDC system.

5.4.3 Reporting Requirements for Pregnancies

5.4.3.1 Pregnancies in Female Patients

As described in Section 5.1.4, female patients of childbearing potential are required to use one highly effective form of contraception or use two effective forms of contraception.

Female patients of childbearing potential will be instructed to immediately inform the investigator if she becomes pregnant during the study or within 7 months after study

"Use of this document is governed by the terms of use on the first page of this document."

treatment. A Pregnancy Report eCRF should be completed by the investigator within 24 hours after learning of the pregnancy and submitted via the EDC system. A pregnancy report will automatically be generated and sent to the Sponsor's Safety Risk Management department. Pregnancy should not be recorded on the Adverse Event eCRF. The investigator should discontinue study drugs and counsel the patient, discussing the risks of the pregnancy and the possible effects on the fetus. Monitoring of the patient should continue **until** conclusion of the pregnancy. Additional information on any Herceptin SC or Perjeta IV-exposed pregnancy and infant will be requested by Roche Drug Safety at specific timepoints (i.e., at the end of the second trimester, 2 weeks after the expected date of delivery, and at 3, 6, and 12 months of the infant's life).

In the event that the EDC system is unavailable, the Clinical Trial Pregnancy Reporting Form provided to investigators should be completed and submitted to Roche or its designee immediately (i.e., no more than 24 hours after learning of the pregnancy), either by faxing or by scanning and emailing the form with use of the fax number or email address provided to investigators. Once the EDC system is available, all information will need to be entered and submitted via the EDC system.

5.4.3.2 Abortions

Any spontaneous abortion should be classified as a serious adverse event (as the Sponsor considers spontaneous abortions to be medically significant events), recorded on the Adverse Event eCRF, and reported to the Sponsor within 24 hours after learning of the event (see Section 5.4.2).

5.4.3.3 Congenital Anomalies/Birth Defects

Any congenital anomaly/birth defect in a child born to a female patient or female partner of a male patient exposed to study drug should be classified as a serious adverse event, recorded on the Adverse Event eCRF and reported to the Sponsor within 24 hours after learning of the event (see Section 5.4.2).

5.5 FOLLOW-UP OF PATIENTS WITH ONGOING ADVERSE EVENTS

5.5.1 Investigator Follow-Up

The investigator should follow each adverse event as described in Section 5.3.1. Every effort should be made to follow all serious adverse events considered to be related to study drug or trial-related procedures until a final outcome can be reported.

During the study period, resolution of adverse events (with dates) should be documented on the Adverse Event eCRF and in the patient's medical record to facilitate source data verification.

"Use of this document is governed by the terms of use on the first page of this document."

All pregnancies reported during the study should be followed until pregnancy outcome. If the EDC system is not available at the time of pregnancy outcome, follow reporting instructions provided in Section 5.4.3.1.

5.5.2 Sponsor Follow-Up

For serious adverse events, non-serious adverse events of special interest, and pregnancies, the Sponsor or a designee may follow-up by telephone, fax, email, and/or a monitoring visit to obtain additional case details and outcome information (e.g., from hospital discharge summaries, consultant reports, autopsy reports) in order to perform an independent medical assessment of the reported case.

5.6 POST-STUDY ADVERSE EVENTS

The Sponsor should be notified if the investigator becomes aware of any serious adverse event that occurs after the end of the adverse event reporting period (see Section 5.3.1), if the event is believed to be related to study drug treatment.

The investigator should report these events directly to Roche or its designee, either by faxing or by scanning and emailing the Serious Adverse Event/Adverse Event of Special Interest Reporting Form with use of the fax number or email address provided to investigators.

5.7 EXPEDITED REPORTING TO HEALTH AUTHORITIES, INVESTIGATORS, INSTITUTIONAL REVIEW BOARDS, AND ETHICS COMMITTEES

The Sponsor will promptly evaluate all serious adverse events and against cumulative product experience to identify and expeditiously communicate possible new safety findings to investigators, IRBs, ECs, and applicable health authorities based on applicable legislation.

To determine reporting requirements for single adverse event cases, the Sponsor will assess the expectedness of these events using the following reference documents:

- Herceptin and Perjeta Investigator's Brochures

The Sponsor will compare the severity of each event and the cumulative event frequency reported for the study with the severity and frequency reported in the applicable reference document.

Reporting requirements will also be based on the investigator's assessment of causality and seriousness with allowance for upgrading by the Sponsor as needed.

5.8 REVIEW OF SAFETY BY AN INTERNAL MONITORING COMMITTEE

An IMC will be established for the study with Roche members who are independent from the BO29159 study team. The IMC membership will include representatives from

"Use of this document is governed by the terms of use on the first page of this document."

clinical science, safety science, and biostatistics. Specific policies on the operation of the IMC will be documented in an IMC Charter.

The IMC will meet on a regular basis over the course of the study and may also meet on an unscheduled basis if any unexpected safety concerns arise.

The details of the IMC will be provided in a separate charter document.

6. STATISTICAL CONSIDERATIONS AND ANALYSIS PLAN

The cutoff for the final analysis will be **24 months** after the last patient is enrolled.

In addition to the final analysis, there will be an interim analysis approximately 6 months after recruitment of the last patient to determine overall safety and tolerability with emphasis on cardiac safety and efficacy. Given the objective of the study, the Sponsor may choose to adapt the timepoint of the interim analysis or to conduct additional interim analyses as appropriate (see Section 6.7).

Efficacy and safety populations will be identical in this study and include all enrolled patients who received at least one dose of any study drug.

6.1 DETERMINATION OF SAMPLE SIZE

The main objective of this safety study is the characterization of the safety profile and tolerability of Herceptin SC in combination with Perjeta IV and docetaxel based on an estimation of the incidence of adverse events. This is not a hypothesis testing study but an exploratory study with predefined precision of estimates for key safety parameters for sample size determination; there are no formal statistical hypothesis tests to be performed, and there will be no adjustments for multiplicity of endpoints or within-subgroups comparisons.

The proposed sample size to be enrolled in this study is 400 patients with the following rationale:

- The width of the 95% Pearson Clopper CI of the incidence of Grade $\geq$3 adverse events is reasonably small (71.8%, 80.3%) with 400 treated patients based on the observed incidence of Grade $\geq$3 adverse events of 76.2% for patients who were treated with Perjeta in combination with Herceptin IV in the TOC4129g/WO20698 study.
- Furthermore, with 400 treated patients on the observed incidence of cardiac events Grade $\geq$3 of 1.7% reported in the TOC4129g/WO20698 study, the width of the 95% Pearson Clopper CI of the incidence of Grade $\geq$3 cardiac adverse events is reasonably small (0.7%, 3.6%).

6.2 SUMMARIES OF CONDUCT OF STUDY

The major protocol deviations will be summarized.

"Use of this document is governed by the terms of use on the first page of this document."

Enrollment, patient disposition, study treatment administration, and discontinuations from the study will be summarized. The incidence of treatment discontinuation for reasons other than disease progression will be tabulated.

The duration of follow-up will be summarized by summary statistics of mean, median, range, standard deviation, and 25th–75th quartiles.

6.3 SUMMARIES OF TREATMENT GROUP COMPARABILITY

There is only one treatment group in this study.

Baseline and disease characteristics, such as demographics, medical history, etc., will be summarized by descriptive statistics (frequency tables for categorical variables and mean, median, range, standard deviation, and 25th–75th quartiles for the continual variables). These characteristics will be summarized for all patients who are enrolled and treated in the study.

6.4 EFFICACY ANALYSES

The efficacy analyses will include all enrolled patients who received at least one dose of any study drug.

Efficacy outcome measures for this study are to evaluate Herceptin SC in combination with Perjeta plus docetaxel with respect to PFS, OS, and ORR.

PFS based on investigator assessment is defined as the time from first dose of study drug administration to the first radiographically documented progression of disease, as determined by the investigator using current RECIST v1.1 (see Appendix 5; Eisenhauer et al. 2009) or death from any cause, whichever occurs first. Carcinomatous meningitis diagnosed by cytologic evaluation of cerebral spinal fluid will also define PD. Medical photography will also be allowed to monitor chest wall recurrences of subcutaneous lesions.

Overall survival: OS is defined as the time from the date of first dose of study drug administration to date of death from any cause.

Objective response: Objective response is defined as a CR or PR determined by the investigator using current RECIST v1.1 (see Appendix 5; Eisenhauer et al. 2009) on two consecutive occasions $\geq$4 weeks apart. Patients with disease localized only to the bone will not be included in the analysis of objective response.

6.5 SAFETY ANALYSES

The safety analyses will include all enrolled patients who received at least one dose of any study drug.

"Use of this document is governed by the terms of use on the first page of this document."

The safety variables are all adverse events, adverse events Grade ≥ 3 according to the NCI CTCAE v4.0, adverse events leading to treatment interruption and discontinuation, serious adverse events, causes of deaths, incidence of CHF, incidence of cardiac adverse events Grade ≥ 3, LVEF decline ($\geq 10\%$ points from baseline to below 50%), premature discontinuation from study and treatment, and laboratory parameters. The primary interest in this study will be to estimate the incidence of adverse events Grade ≥ 3 for the treatment of Herceptin SC in combination with Perjeta and docetaxel.

The analysis of adverse events will focus on treatment-emergent adverse events (i.e., adverse events that occur during or after the first administration of study drug). Non–treatment-emergent adverse events (i.e., those that occur during screening) will be listed only during the screening period. Only the serious adverse event related to a protocol-mandated procedure will be reported and all adverse events that occurred before Day 1 (first administration) would be reported in medical history.

The incidence, type, and severity of adverse events will be summarized according to the primary System Organ Class (SOC) and within each SOC, by MedDRA preferred term.

Adverse events Grade ≥ 3, adverse events leading to treatment modification and discontinuation, and serious adverse events will be analyzed in a similar way to all adverse events. Causes of deaths will also be summarized and listed.

LVEF as well as changes from baseline over time will be analyzed using descriptive statistics for continuous variable and presented graphically over time with associated 95% CI. The percentage of patients with an LVEF decline $\geq 10\%$ points from baseline to below 50% will be summarized.

The number of patients who prematurely discontinue from study treatment with a corresponding reason for discontinuation will be summarized and listed.
The discontinuation from study will be also summarized and listed.

Descriptive statistics will be presented for cumulative study medication doses and duration of exposure.

Subgroup analysis of all grade adverse event variables will be performed for patients receiving at least one cycle of 100 mg/m^2 docetaxel.

The following subgroup analysis will be performed for LVEF decline of more than 10% points from baseline to below 50%, CHF, cardiac adverse events Grade ≥ 3:

- Other selected safety variables—race
- Known risk factors for development of cardiac related events—age, medical history of hypertension, prior treatment with anthracyclines, LVEF at baseline

"Use of this document is governed by the terms of use on the first page of this document."

The incidence and severity of ARR adverse events will be summarized. Time to onset of the first ARRs and time from onset to resolution of ARRs will also be summarized. In addition, the ARR analyses will also be performed for each treatment cycle.

Laboratory parameters, hematology, and serum biochemistry will be presented in shift tables of NCI CTCAE v4.0 grade at baseline versus worst grade during the treatment period. The summary of laboratory parameters will also be presented by means, standard deviation, minimum, and maximum. The selected laboratory parameters will be also graphically presented over time.

6.6 IMMUNOGENICITY ANALYSES

Immunogenicity analyses will be performed using the safety population. Incidence of baseline and post-baseline anti-Herceptin and anti-rHuPH20 antibody rates will be summarized. All patients with at least one anti-Herceptin and/or anti- rHuPh20 confirmed positive result will be listed. Calculation of mean Herceptin concentration by antibody status (positive/negative) at each antibody timepoint will be performed to assist in the assessment of antibody development. Subgroup analysis by antibody status will be performed for incidence of ARRs.

6.7 INTERIM ANALYSES

In addition to the final analysis, there will be an interim analysis approximately 6 months after recruitment of the last patient to determine overall safety and tolerability with special emphasis on cardiac safety and efficacy. Given the objective of the study, the Sponsor may choose to adapt the timepoint of the interim analysis or to conduct additional interim analyses if appropriate. The decision to adapt the timepoint of the interim analysis or to conduct an additional interim analysis will be documented in the Sponsor’s trial master file prior to the conduct of the respective interim analysis. The interim analysis will be performed and interpreted by Sponsor study team personnel.

There will also be an annual review of safety data by the IMC.

7. DATA COLLECTION AND MANAGEMENT

7.1 DATA QUALITY ASSURANCE

The Sponsor will supply eCRF specifications for this study. A contract research organization (CRO) will be responsible for data management of this study, including quality checking of the data. Data entered manually will be collected via EDC using eCRFs. Sites will be responsible for data entry into the EDC system. In the event of discrepant data, the CRO will request data clarification from the sites, which the sites will resolve electronically in the EDC system.

The CRO will produce a Data Quality Plan that describes the quality checking to be performed on the data.

"Use of this document is governed by the terms of use on the first page of this document."

The Sponsor will perform oversight of the data management of this study, including approval of the CRO's data management plans and specifications. Data will be periodically transferred electronically from the CRO to the Sponsor, and the Sponsor's standard procedures will be used to handle and process the electronic transfer of these data.

eCRFs and correction documentation will be maintained in the EDC system's audit trail. System backups for data stored at the CRO and records retention for the study data will be consistent with the CRO's standard procedures.

7.2 ELECTRONIC CASE REPORT FORMS

eCRFs are to be completed using a EDC system. Sites will receive training and have access to a manual for appropriate eCRF completion. eCRFs will be submitted electronically to the EDC system and should be handled in accordance with instructions from the Sponsor.

All eCRFs should be completed by designated, trained site staff. eCRFs should be reviewed and electronically signed and dated by the investigator or a designee.

At the end of the study, the investigator will receive patient data for his or her site in a readable format on a compact disc that must be kept with the study records. Acknowledgement of receipt of the compact disc is required.

7.3 SOURCE DATA DOCUMENTATION

Study monitors will perform ongoing source data verification to confirm that critical protocol data entered into the eCRFs by authorized site personnel are accurate, complete, and verifiable from source documents.

Source documents (paper or electronic) are those in which patient data are recorded and documented for the first time. They include but are not limited to hospital records, clinical and office charts, laboratory notes, memoranda, patient-reported outcomes, evaluation checklists, pharmacy dispensing records, recorded data from automated instruments, copies of transcriptions that are certified after verification as being accurate and complete, microfiche, photographic negatives, microfilm or magnetic media, X-rays, patient files, and records kept at pharmacies, laboratories, and medico-technical departments involved in a clinical trial.

Before study initiation, the types of source documents that are to be generated will be clearly defined in the Trial Monitoring Plan. This includes any protocol data to be entered directly into the eCRFs (i.e., no prior written or electronic record of the data) and considered source data.

"Use of this document is governed by the terms of use on the first page of this document."

Source documents that are required to verify the validity and completeness of data entered into the eCRFs must not be obliterated or destroyed and must be retained per the Roche policy for retention of records described in Section 7.5.

To facilitate source data verification, the investigators and institutions must provide the Sponsor direct access to applicable source documents and reports for trial-related monitoring, Sponsor audits, and IRB or IEC review. The investigational site must also allow inspection by applicable health authorities.

7.4 USE OF COMPUTERIZED SYSTEMS

When clinical observations are entered directly into an investigational site's computerized medical record system (i.e., in lieu of original hardcopy records), the electronic record can serve as the source document if the system has been validated in accordance with health authority requirements pertaining to computerized systems used in clinical research. An acceptable computerized data collection system allows preservation of the original entry of data. If original data are modified, the system should maintain a viewable audit trail that shows the original data as well as the reason for the change, name of the person making the change, and date of the change.

7.5 RETENTION OF RECORDS

Records and documents pertaining to the conduct of this study and the distribution of IMP, including eCRFs, Informed Consent Forms, laboratory test results, and medication inventory records, must be retained by the Principal Investigator for at least 15 years after completion or discontinuation of the study or for the length of time required by relevant national or local health authorities, whichever is longer. After that period of time, the documents may be destroyed, subject to local regulations.

No records may be disposed of without the written approval of the Sponsor. Written notification should be provided to the Sponsor prior to transferring any records to another party or moving them to another location.

8. ETHICAL CONSIDERATIONS

8.1 COMPLIANCE WITH LAWS AND REGULATIONS

This study will be conducted in full conformance with the ICH E6 guideline for Good Clinical Practice and the principles of the Declaration of Helsinki or the laws and regulations of the country in which the research is conducted, whichever affords the greater protection to the individual. The study will comply with the requirements of the ICH E2A guideline (Clinical Safety Data Management: Definitions and Standards for Expedited Reporting; Appendix 2). Studies conducted in the United States or under a U.S. Investigational New Drug (IND) application will comply with U.S. Food and Drug Administration (FDA) regulations and applicable local, state, and federal laws. Studies conducted in the European Union/European Economic Area will comply with the EU Clinical Trial Directive (2001/20/EC).

"Use of this document is governed by the terms of use on the first page of this document."

8.2 INFORMED CONSENT

The Sponsor's sample Informed Consent Form will be provided to each site. If applicable, it will be provided in a certified translation of the local language. The Sponsor or its designee must review and approve any proposed deviations from the Sponsor's sample Informed Consent Form or any alternate consent forms proposed by the site (collectively, the "Consent Forms") before IRB or IEC submission. The final IRB or IEC-approved Consent Forms must be provided to the Sponsor for health authority submission purposes according to local requirements.

The Consent Forms must be signed and dated by the patient or the patient's legally authorized representative before her participation in the study. The case history or clinical records for each patient shall document the informed consent process and that written informed consent was obtained prior to participation in the study.

The Consent Forms should be revised whenever there are changes to study procedures or when new information becomes available that may affect the willingness of the patient to participate. The final revised IRB or IEC-approved Consent Forms must be provided to the Sponsor for health authority submission purposes.

Patients must be re-consented to the most current version of the Consent Forms (or to a significant new information/findings addendum in accordance with applicable laws and IRB or IEC policy) during their participation in the study. For any updated or revised Consent Forms, the case history or clinical records for each patient shall document the informed consent process and that written informed consent was obtained using the updated/revised Consent Forms for continued participation in the study.

A copy of each signed Consent Form must be provided to the patient or the patient's legally authorized representative. All signed and dated Consent Forms must remain in each patient's study file or in the site file and must be available for verification by study monitors at any time.

8.3 INDEPENDENT ETHICS COMMITTEE

This protocol, the Informed Consent Forms, any information to be given to the patient, and relevant supporting information must be submitted to the IRB or IEC by the Principal Investigator and reviewed and approved by the IRB or IEC before the study is initiated. In addition, any patient recruitment materials must be approved by the IRB or IEC.

The Principal Investigator is responsible for providing written summaries of the status of the study to the IRB or IEC annually or more frequently in accordance with the requirements, policies, and procedures established by the IRB or IEC. Investigators are also responsible for promptly informing the IRB or IEC of any protocol amendments (see Section 9.6).

"Use of this document is governed by the terms of use on the first page of this document."

In addition to the requirements for reporting all adverse events to the Sponsor, investigators must comply with requirements for reporting serious adverse events to the local health authority and IRB/IEC. Investigators may receive written IND safety reports or other safety-related communications from the Sponsor. Investigators are responsible for ensuring that such reports are reviewed and processed in accordance with health authority requirements and the policies and procedures established by their IRB or IEC, and archived in the site's study file.

8.4 CONFIDENTIALITY

The Sponsor maintains confidentiality standards by coding each patient enrolled in the study through assignment of a unique patient identification number. This means that patient names are not included in data sets that are transmitted to any Sponsor location.

Patient medical information obtained by this study is confidential and may only be disclosed to third parties as permitted by the Informed Consent Form (or separate authorization for use and disclosure of personal health information) signed by the patient, unless permitted or required by law.

Medical information may be given to a patient's personal physician or other appropriate medical personnel responsible for the patient's welfare, for treatment purposes.

Data generated by this study must be available for inspection upon request by representatives of the FDA and other national and local health authorities, Sponsor monitors, representatives, and collaborators, and the IRB or IEC for each study site, as appropriate.

8.5 FINANCIAL DISCLOSURE

Investigators will provide the Sponsor with sufficient, accurate financial information in accordance with local regulations to allow the Sponsor to submit complete and accurate financial certification or disclosure statements to the appropriate health authorities. Investigators are responsible for providing information on financial interests during the course of the study and for 1 year after completion of the study.

9. STUDY DOCUMENTATION, MONITORING, AND ADMINISTRATION

9.1 STUDY DOCUMENTATION

The investigator must maintain adequate and accurate records to enable the conduct of the study to be fully documented including but not limited to the protocol, protocol amendments, Informed Consent Forms, and documentation of IRB or IEC and governmental approval. In addition, at the end of the study the investigator will receive the patient data, which includes an audit trail containing a complete record of all changes to data.

"Use of this document is governed by the terms of use on the first page of this document."

9.2 PROTOCOL DEVIATIONS

The investigator should document and explain any protocol deviations. The investigator should promptly report any deviations that might have an impact on patient safety and data integrity to the Sponsor and to the IRB/EC in accordance with established IRB/EC policies and procedures.

9.3 SITE INSPECTIONS

Site visits will be conducted by the Sponsor or an authorized representative for inspection of study data, patients' medical records, and eCRFs. The investigator will permit national and local health authorities, Sponsor monitors, representatives, and collaborators, and the IRB or IECs to inspect facilities and records relevant to this study.

9.4 ADMINISTRATIVE STRUCTURE

An IxRS system will be used for enrollment of patients into the study.

A CRO will be used for data management (see Section 7.1).

Assessment of laboratory test results will be performed locally.

9.5 PUBLICATION OF DATA AND PROTECTION OF TRADE SECRETS

Regardless of the outcome of a trial, the Sponsor is dedicated to openly providing information on the trial to healthcare professionals and to the public, both at scientific congresses and in peer-reviewed journals. The Sponsor will comply with all requirements for publication of study results. For more information, refer to the Roche Global Policy on Sharing of Clinical Trials Data at the following Web site:

http://www.rochetrials.com/pdf/RocheGlobalDataSharingPolicy.pdf.

The results of this study may be published or presented at congresses. For all clinical trials in patients involving an IMP for which a marketing authorization application has been filed or approved in any country, the Sponsor aims to submit a journal manuscript reporting primary clinical trial results within 6 months after the availability of the respective clinical study report. In addition, for all clinical trials in patients involving an IMP for which a marketing authorization application has been filed or approved in any country, the Sponsor aims to publish results from analyses of additional endpoints and exploratory data that are clinically meaningful and statistically sound.

The investigator must agree to submit all manuscripts or abstracts to the Sponsor prior to submission for publication or presentation. This allows the Sponsor to protect proprietary information and to provide comments based on information from other studies that may not yet be available to the investigator.

"Use of this document is governed by the terms of use on the first page of this document."

In accordance with standard editorial and ethical practice, the Sponsor will generally support publication of multicenter trials only in their entirety and not as individual center data. In this case, a coordinating investigator will be designated by mutual agreement.

Authorship will be determined by mutual agreement and in line with International Committee of Medical Journal Editors authorship requirements. Any formal publication of the study in which contribution of Sponsor personnel exceeded that of conventional monitoring will be considered as a joint publication by the investigator and the appropriate Sponsor personnel.

Guidance from International Committee of Medical Journal Editors (ICMJE):

- Authors must meet ALL of the following authorship criteria: Substantial contributions to conception and design, acquisition of data, and analysis and interpretation of data.
- Drafting or revising the abstract/manuscript for important intellectual content
- Final approval of the version to be published
- Accountability for all aspects of the work by ensuring that questions related to the accuracy of any part of the work are appropriately investigated and resolved
- When a large, multicenter group has conducted the work, the group should identify the individuals who accept direct responsibility for manuscript. These individuals should fully meet the criteria for authorship defined above.
- Acquisition of funding, collection of data, or general supervision of the research group alone does not justify authorship.
- All persons designated as authors should qualify for authorship, and all those who qualify should be listed.
- Each author should have participated sufficiently in the work to take public responsibility for appropriate portions of the content.

Any inventions and resulting patents, improvements, and/or know-how originating from the use of data from this study will become and remain the exclusive and unburdened property of the Sponsor, except where agreed otherwise.

9.6 PROTOCOL AMENDMENTS

Any protocol amendments will be prepared by the Sponsor. Protocol amendments will be submitted to the IRB/EC and to regulatory authorities in accordance with local regulatory requirements.

Approval must be obtained from the IRB or IEC and regulatory authorities (as locally required) before implementation of any changes, except for changes necessary to eliminate an immediate hazard to patients or changes that involve logistical or administrative aspects only (e.g., change in Medical Monitor or contact information).

"Use of this document is governed by the terms of use on the first page of this document."

10. REFERENCES

American Cancer Society. Breast Cancer. Available through: www.cancer.org [Accessed 29 June 2011].

Baselga J. Treatment of HER2-overexpressing breast cancer. Ann Oncol 2010;Suppl 7:vii:36–40.

Baselga J, Cameron D, Miles D, et al. Objective response rate in a Phase II multicenter trial of pertuzumab (P), a HER2 dimerization inhibiting monoclonal antibody, in combination with trastuzumab (T) in patients (pts) with HER2-positive metastatic breast cancer (MBC) which has progressed during treatment with T. J Clin Oncol 2007 (ASCO Annual Meeting Proceedings);25:18S:1004.

Baselga J, Cortés J, Kim SB, et al. Pertuzumab plus trastuzumab plus docetaxel for metastatic breast cancer. N Engl J Med 2012;366:109–19.

Chang J, Clark GM, Allred DC, et al. Survival of patients with metastatic breast cancer. Importance of prognostic markers of the primary tumor. Cancer 2003;97:545–53.

Cho HS, Mason K, Ramyar KX, et al. Structure of the extracellular region of HER2 alone and in complex with the Herceptin Fab. Nature 2003;421:756–60.

Eisenhauer EA, Therasse P, Bogaerts J, et al. New response evaluation criteria in solid tumours: revised RECIST guideline (version 1.1). Eur J Cancer 2009;45:228–47

Ferlay J, Hai-Rim S, Bray F, et al. Estimates of worldwide burden of cancer in 2008: GLOBOCAN 2008. Int J Cancer 2010;127:2893–917.

Franklin MC, Carey KD, Vajdos FF, et al. Insights into ErbB signaling from the structure of the ErbB2-pertuzumab complex. Cancer Cell 2004;5:317–28.

Frost GI. Recombinant human hyaluronidase (rHUPH20): an enabling platform for subcutaneous drug and fluid administration. Expert Opin Drug Deliv 2007;4:427–40.

Geyer CE, Forster J, Lindquist D, et al. Lapatinib plus capecitabine for HER2-positive advanced breast cancer. N Engl J Med 2006;355:2733–43.

Herceptin Summary of Product Characteristics. www.medicines.org.uk/emc/document.aspx?documentId=3567 [Accessed 9 June 2011].

Hylenex® Prescribing Information, Halozyme Therapeutics, Inc. September 2012 [cited 29 October 2013]. Available from: www.hylenex.com/files/HylenexPackageInsertSep2012.pdf

Ismael G, Hegg R, Muehlbauer S, et al. Subcutaneous versus intravenous administration of (neo)adjuvant trastuzumab in patients with HER2-positive, clinical Stage I–III breast cancer (HannaH study): a phase 3, open-label, multicentre, randomised trial. The Lancet 2012;13:869–78.

"Use of this document is governed by the terms of use on the first page of this document."

Jones RB, Gordus A, Krall JA, et al. A quantitative protein interaction network for the ErdB receptors using protein microarrays. Nature 2006;439:168–74.

Lenihan D, Suter T, Brammer M, et al. Pooled analysis of cardiac safety in patients with cancer treated with pertuzumab. Ann Oncol 2012;23:791–800.

Marty M, Cognetti F, Maraninchi D, et al. Randomized Phase II trial of the efficacy and safety of trastuzumab combined with docetaxel in patients with human epidermal growth factor receptor 2 positive metastatic breast cancer administered at first line treatment (The M77001 Study group). J Clin Oncol 2005;23:4265–74.

National Cancer Institute. Surveillance epidemiology and end results. [resource on the internet]. [cited 9 June 2011]. Available from: www.seer.cancer.gov.

Pauletti G, Dandekar S, Rong H, et al. Assessment of methods for tissue-based detection of the HER-2neu alteration in human breast cancer: a direct comparison of fluorescent in-situ hybridization and immunohistochemistry. J Clin Oncol 2000;18:3651–64.

Pivot X, Gligorov J, Müller V, et al. Preference for subcutaneous or intravenous administration of trastuzumab in patients with HER2–positive early breast cancer (PrefHer): an open-label randomised study. Lancet Oncol 2013;14:962–70.

Romond EH, Perez EA, Bryant J, et al. Trastuzumab plus adjuvant chemotherapy for operable HER2-positive breast cancer. N Engl J Med 2005;353:1673–84.

Scheuer W, Friess T, Burtscher H, et al. Strongly enhanced antitumor activity of trastuzumab and pertuzumab combination treatment on HER2-positive human xenograft tumor models. Cancer Res. 2009;69:9330–6.

Slamon DJ, Leyland-Jones B, Shak S, et al. Use of chemotherapy plus a monoclonal antibody against HER-2 for metastatic breast cancer. N Engl J Med 2001;344:783–92.

Smith TJ, Khatcheressian J, Lyman GH, et al. Update of recommendations for the use of white blood cell growth factors: an evidence-based clinical practice guideline. J Clin Oncol 2006;24:3187–205.

Sundaresan S, Penuel E, Sliwkowski MX. The biology of human epidermal growth factor receptor 2. Curr Oncol Rep 1999;1:16–22.

Swain SM, Ewer MS, Cortes J, et al. Cardiac tolerability of pertuzumab plus trastuzumab plus docetaxel in patients with HER2-positive metastatic breast cancer in CLEOPATRA: a randomized, double-blind, placebo-controlled phase III study. Oncologist 2013a;18:257–64.

Swain SM, Kim SB, Cortes J, et al. Pertuzumab, trastuzumab, and docetaxel for HER2-positive metastatic breast cancer (CLEOPATRA study): overall survival results from a randomized, double-blind, placebo-controlled, phase 3 study. Lancet Oncol 2013b;14:461–71.

"Use of this document is governed by the terms of use on the first page of this document."

Weatherall DJ, Ledingham JGG, editors. Oxford textbook of medicine. Third edition. New York: Oxford University Press, 1996.

Yarden Y, Sliwkowski MX. Untangling the ErbB signalling network. Nature Rev Mol Cell Biol 2001;2:127–37.

"Use of this document is governed by the terms of use on the first page of this document."

Appendix 1
Schedule of Assessments

	Screening	Baseline [a]	Treatment Period	Post-Treatment Follow-Up		
				Safety Follow-Up Visit Post-Treatment 28–35 Days after Last Dose of Study Treatment [c]	Follow-Up Every 12 Weeks ± 7 Days after Safety Follow-Up Visit	
	Day −28 to Day 1	Day −7 to Day 1	Each Treatment Cycle [b] (All Visits within ± 3 Days of Scheduled Treatment Day)		Until Disease Progression	After Disease Progression Until the End of the Study
Informed consent	x					
HER2 [d] status	If positive HER2 result not available					
Hormonal receptor status from primary tumor (and/or metastatic site, if primary tumor not available)	x					
Demographics and medical history [e]	x					
Menopausal status	x					
Tumor evaluation [f]	x		Every 9 weeks (± 3 days) from the first administration until disease progression			
Brain CT/MRI	x (if clinical suspicion of brain metastasis)		If clinically indicated			
Standard 12-lead ECG [g]	x		Every 3 cycles of monoclonal ant body (within 7 days **prior** to study treatment)	x		
LVEF [h] (ECHO or MUGA)	x			x	x	
Concomitant medications [i]	x	x	x	x	x	
Physical examination [j]		x	x	x		

"Use of this document is governed by the terms of use on the first page of this document."

Appendix 1
Schedule of Assessments (cont.)

	Screening	Baseline [a]	Treatment Period	Post-Treatment Follow-Up		
				Safety Follow-Up Visit Post-Treatment 28–35 Days after Last Dose of Study Treatment [c]	Follow-Up Every 12 Weeks ± 7 Days after Safety Follow-Up Visit	
	Day –28 to Day 1	Day –7 to Day 1	Each Treatment Cycle [b] (All Visits within ± 3 Days of Scheduled Treatment Day)		Until Disease Progression	After Disease Progression Until the End of the Study
Vital signs and blood pressure [j]		x	x	x	x	
Height		x				
Weight [j]		x	x	x		
Pregnancy test [k]		x	Every 3 cycles of monoclonal antibody prior to drug administration.	x	About 4 months and 7 months after discontinuation of study treatment (during the closest follow-up visit)	
Hematology [l] and serum chemistry (local laboratory)		x	x	x		
INR and aPTT or PTT [l] (local lab)		x	x	x		
Blood sample for anti-trastuzumab and serum concentration analysis [m]		x	Pre-dose Cycle 2	x		
Blood sample for anti-rHuPH20 analysis [m]		x	Pre-dose Cycle 2	x		
ECOG Performance Status		x	Every 3 cycles of monoclonal antibody	x	x	
Serious adverse events/adverse events [n]	x	x	x	x	x (see Section 5.3.1)	x (see Section 5.3.1)
Record of post-study treatment cancer-related medical or surgical procedures [o]				x	x	x
Administration of study medication			x			

"Use of this document is governed by the terms of use on the first page of this document."

Appendix 1
Schedule of Assessments (cont.)

	Screening	Baseline [a]	Treatment Period	Post-Treatment Follow-Up		
				Safety Follow-Up Visit Post-Treatment 28–35 Days after Last Dose of Study Treatment [c]	Follow-Up Every 12 Weeks ± 7 Days after Safety Follow-Up Visit	
	Day –28 to Day 1	Day –7 to Day 1	Each Treatment Cycle [b] (All Visits within ± 3 Days of Scheduled Treatment Day)		Until Disease Progression	After Disease Progression Until the End of the Study
Administration-related reactions during infusion and observation period			x			
Survival [p]					x	x

ECHO = echocardiogram; ECOG = Eastern Cooperative Oncology Group; HER2 = human epidermal growth factor 2; LVEF = left ventricular ejection fraction; MUGA = multiple-gated acquisition; rHuPH20 = recombinant human hyaluronidase.

[a] Baseline/Screening assessments are allowable on Day 1 of first treatment cycle before dose as long as the results are available prior to first dose of study drug administration.

[b] Cycle = 3 weeks for monoclonal antibodies.

[c] At the post-treatment safety follow-up visit, all the assessments and sample collection has to be done before the start of any subsequent line of treatment.

[d] Demonstrated evidence of HER2-positive status from previous testing is acceptable. Otherwise HER2-positive status on fixed tissue blocks from the primary tumor (and/or metastatic site, if primary tumor not available) to be assessed locally by immunohistochemistry and/or in situ hybridization according to institutional criteria.

[e] Complete medical history (clinically significant diseases, surgeries, cancer history, etc.) and demographics (i.e., age, sex, race, and self-reported ethnicity, if applicable) and all medications taken within 28 days prior to the first dose administration (Day 1).

[f] A CT or MRI of the chest, abdomen, and pelvis and (if indicated) isotope bone scan (evaluation according to RECIST v1.1 criteria) should be performed at screening and as clinically indicated. Scans at screening should not be older than 28 days prior to **first study medication administration**. To be performed post-study treatment only if disease progression has not yet been established. Note: Bone scan, PET scan, or plain films are not considered adequate imaging techniques to measure bone lesions. Always schedule tumor assessments every 9 weeks ± 3 days from the date of first drug administration. If a tumor assessment must be performed early or late, subsequent assessments should be conducted according to the original schedule of every 9 weeks from the date of first dose of study drug administration. All patients should have a minimum of a **chest** and **abdominal CT scan**. PET scans will not be considered for assessments of efficacy at any time during the study (except as specified for bone scans in the absence of radioactive isotopes).

[g] ECG will be performed at screening (within 6 weeks prior to first dose of study drug) and **every three cycles** of monoclonal antibody therapy during the treatment period, at the time of LVEF measurement. ECGs should be performed within 7 days prior to administration of study drug.

"Use of this document is governed by the terms of use on the first page of this document."

[h] LVEF ≥ 50% at screening period to be determined by either ECHO or MUGA scan (with ECHO as the preferred method). The **same** method of LVEF assessment (ECHO or MUGA) must be used for the same patient throughout the study and, to the extent possible, be obtained at the same institution. All pre-study LVEF values during and following Herceptin adjuvant treatment for patients who received such adjuvant therapy prior to first dose of study drug administration into the study will be collected. LVEF assessment (ECHO or MUGA) during the screening period within 6 weeks prior to first dose of study drug does not need to be repeated. To be performed **every three cycles within 7 days of monoclonal antibody therapy** during the treatment period and at safety follow-up visit if previous result is older than 9 weeks. If an LVEF assessment must be performed early or late, subsequent assessments should be conducted according to the original schedule from the date of first dose of study drug administration. After post-treatment follow-up visit, LVEF assessment will be done **every 6 months** in the first year and then **annually for up to 2 years** after post-treatment safety follow-up visit at 6, 12, and 24 months. Patients for whom study treatment was permanently discontinued because of a drop in LVEF should continue to have LVEF assessments repeated as clinically indicated, with a maximum interval between LVEF assessments of 3 months until the LVEF values return to ≥ 50% or until 1 year after post-treatment safety follow-up visit at 3, 6, 9, 12, and 24 months, whichever occurs first. Thereafter, LVEF assessments will be performed annually for up to 2 years after post-treatment safety follow-up visit.

[i] Concomitant medication will be recorded at baseline and on an ongoing basis until disease progression or end of the study, whichever occurs first.

[j] Physical examination, including vital signs, will be performed prior to first dose of study drug administration with particular care taken with regard to cardiovascular signs and symptoms (elevated jugular venous pressure, sinus tachycardia, tachypnea, the presence of an S3 heart sound, crackles on chest auscultation, etc.). A physical examination must be performed within 7 days prior to baseline. Vital signs will be assessed **before** treatment on Day 1 of every treatment cycle (Perjeta, Herceptin, and docetaxel) with blood pressure, pulse rate, and body temperature recorded **again after** administration during the observation period **of each** study medication.

[k] All women of childbearing potential (premenopausal women and for women < 12 months after the onset of menopause, unless they have undergone surgical sterilization) will have a serum pregnancy test at a local laboratory within 7 days prior to the first administration of study medication. For all other women, documentation must be present in medical history confirming that the patient is not of childbearing potential. Urine pregnancy tests will be repeated during the treatment period within 7 days prior to every third treatment cycle starting at Cycle 3 (and as clinically indicated), as well as at the post-treatment safety follow-up visit and 4 and 7 months after discontinuation of study treatment. Any positive urine pregnancy test must be confirmed with a serum β-HCG evaluation at the local laboratory. Pregnancy test results must be available prior to the next scheduled study treatment. Women who have undergone surgical sterilization or are postmenopausal are exempt from pregnancy assessments.

[l] Assessment must be performed with results available within 3 days prior to the administration of study medication. Hematology will include hemoglobin, hematocrit, platelet count, RBC, WBC with differential (neutrophils, lymphocytes, monocytes, eosinophils, basophils, and other cells). Serum chemistry will include sodium, potassium, calcium, chloride, magnesium, BUN (urea), total protein, albumin, alkaline phosphatase, ALT, AST, gamma-glutamyl transferase, LDH, total bilirubin, creatinine, blood glucose, and calculated creatinine clearance at baseline. All patients will have INR and aPTT or PTT testing at baseline. Tests will be repeated at each treatment cycle in all patients receiving therapeutic doses of anticoagulants. Assessment of coagulation must be performed within 3 days (with results available) prior to the administration of study medication.

[m] Samples for anti-Herceptin ant body and serum concentration and anti-rHuPh20 antibody are collected at baseline, prior to dosing on Day 1 of Cycle 2 and prior to any new treatment at the post-treatment safety follow-up 28–35 days after the last dose of Herceptin SC.

[n] After informed consent, but prior to initiation of study medications, only serious adverse events considered to be related to a protocol-mandated intervention will be collected. Adverse events to be monitored continually during the treatment period. See the details of adverse events reporting period Section 5.3.1 .

[o] Collect post-study treatment cancer-related medical or surgical procedures and therapies and survival information every 12 weeks after the treatment discontinuation visit during the post-treatment follow-up period until death, loss to follow-up, withdrawal of consent, or study termination by Roche.

[p] Survival status will be recorded every 12 weeks after the post-treatment safety follow-up 28–35 days after last dose of study treatment until the end of the study.

"Use of this document is governed by the terms of use on the first page of this document."

Appendix 2
ICH Guidelines for Clinical Safety Data Management, Definitions, and Standards for Expedited Reporting, Topic E2a

A serious adverse event is any experience that suggests a significant hazard, contraindication, side effect or precaution. It is any adverse event that at any dose fulfills at least one of the following criteria:

- Is fatal (results in death) (NOTE: death is an outcome, not an event)
- Is life-threatening (NOTE: the term "life-threatening" refers to an event in which the patient was at immediate risk of death at the time of the event; it does not refer to an event that could hypothetically have caused a death had it been more severe.)
- Requires in-patient hospitalization or prolongation of existing hospitalization
- Results in persistent or significant disability/incapacity
- Is a congenital anomaly/birth defect
- Is medically significant or requires intervention to prevent one or other of the outcomes listed above.

Medical and scientific judgment should be exercised in deciding whether expedited reporting to the Sponsor is appropriate in other situations, such as important medical events that may not be immediately life-threatening or result in death or hospitalization but may jeopardize the patient or may require intervention to prevent one of the outcomes listed in the definitions above. These situations should also usually be considered serious.

Examples of such events are intensive treatment in an emergency room or at home for allergic bronchospasm, blood dyscrasias or convulsions that do not result in hospitalization, or development of drug dependency or drug abuse.

An unexpected adverse event is the nature or severity of which is not consistent with the applicable product information.

Causality is initially assessed by the investigator. For serious adverse events, possible causes of the event **are** indicated by selecting one or more options. (Check all that apply.)

- Preexisting/underlying disease—specify
- Study treatment—specify the drug(s) related to the event
- Other treatment (concomitant or previous)—specify
- Protocol-related procedure
- Other (e.g., accident, new or intercurrent illness)—specify

"Use of this document is governed by the terms of use on the first page of this document."

The term severe is a measure of intensity; thus, a severe adverse event is not necessarily serious. For example, nausea of several hours' duration may be rated as severe but may not be clinically serious.

Such preliminary reports will be followed by detailed descriptions later, which will include copies of hospital case reports, autopsy reports, and other documents when requested and applicable.

For serious adverse events, the following must be assessed and recorded on the adverse events page of the eCRF: intensity, relationship to test substance, action taken, and outcome to date.

The investigator must notify the IRB or IEC of a serious adverse event in writing as soon as is practical and in accordance with international and local laws and regulations.

SPONSOR LOCAL COUNTRY CONTACT for serious adverse events: Local Monitor.

SPONSOR HEADQUARTERS CONTACT for serious adverse events and other medical emergencies: Contact information for the Contract Research Organization responsible for drug safety will be provided separately.

24-HOUR MEDICAL COVERAGE: Identification of a contact for 24-Hour Medical Coverage is mandatory to be compliant with worldwide Regulatory Agencies and to ensure the safety of study patients.

An Emergency Medical Call Center Help Desk will access the Sponsor Medical Emergency List, escalate emergency medical calls, provide medical translation service (if necessary), connect the investigator with the Sponsor medical contact for this study and track all calls. The Emergency Medical Call Center Help Desk will be manned 24 hours, 7 days a week. Toll-free numbers will be distributed to all investigators participating in this clinical trial. The Help Desk will be used for medical emergencies outside regular business hours or when the regular International Medical Leader cannot be reached.

See the Protocol Administrative and Contact Information & List of Investigators form for details of administrative, contact information, and Emergency Medical Call Center Help Desk toll-free numbers. This information will be provided separately.

"Use of this document is governed by the terms of use on the first page of this document."

Appendix 3
Eastern Cooperative Oncology Group Performance Status

Grade	Scale
0	Fully active, able to carry on all pre-disease performance without restriction.
1	Restricted in physically strenuous activity but ambulatory and able to carry out work of a light or sedentary nature, i.e., light housework, office work.
2	Ambulatory and capable of all self-care but unable to carry out any work activities. Up and about more than 50% of waking hours.
3	Capable of only limited self-care, confined to bed or chair more than 50% of waking hours.
4	Completely disabled. Cannot carry out any self-care. Totally confined to bed or chair.
5	Dead.

"Use of this document is governed by the terms of use on the first page of this document."

Appendix 4
New York Heart Association Classification and Left Ventricular Systolic Dysfunction National Cancer Institute Common Terminology Criteria for Adverse Events Version 4.0 Grading

Class I	Patients with cardiac disease but without resulting limitations of physical activity. Ordinary physical activity does not cause undue fatigue, palpitation, dyspnoea or angina pain.
Class II	Patients with cardiac disease resulting in slight limitations of physical activity. They are comfortable at rest. Ordinary physical activity results in fatigue, palpitation, dyspnoea or anginal pain.
Class III	Patients with cardiac disease resulting in marked limitations of physical activity. They are comfortable at rest. Less than ordinary physical activity causes fatigue, palpitation, dyspnoea or anginal pain
Class IV	Patients with cardiac disease resulting in inability to carry on any physical activity without discomfort. Symptoms of cardiac insufficiency or of the angina syndrome may be present even at rest. If any physical activity is undertaken, discomfort is increased.

Weatherall DJ, Ledingham JGG, editors. Oxford Textbook of Medicine. Third Edition. New York: Oxford University Press, 1996.

NCI CTCAE v4.0 Grading

Cardiac disorders					
	Grade				
Adverse Event	1	2	3	4	5
Left ventricular systolic dysfunction	-	-	Symptomatic due to drop in ejection fraction responsive to intervention	Refractory or poorly controlled heart failure due to drop in ejection fraction; intervention such as ventricular assist device, intravenous vasopressor support, or heart transplant indicated	Death
Definition: A disorder characterized by failure of the left ventricle to produce adequate output despite an increase in distending pressure and in end-diastolic volume. Clinical manifestations mayinclude dyspnea, orthopnea, and other signs and symptoms of pulmonary congestion and edema.					
Heart failure	Asymptomatic with laboratory (e.g., BNP [B-Natriuretic Peptide]) or cardiac imaging abnormalities	Symptoms with mild to moderate activity or exertion	Severe with symptoms at rest or with minimal activity or exertion; intervention indicated	Life-threatening consequences; urgent intervention indicated (e.g., continuous IV therapy or mechanical hemodynamic support)	Death
Definition: A disorder characterized by the inability of the heart to pump blood at an adequate volume to meet tissue metabolic requirements, or, the ability to do so only at an elevation in the filling pressure.					

"Use of this document is governed by the terms of use on the first page of this document."

Appendix 4
New York Heart Association Classification and Left Ventricular Systolic Dysfunction National Cancer Institute Common Terminology Criteria for Adverse Events Version 4.0 Grading (cont.)

Investigations					
	Grade				
Adverse Event	1	2	3	4	5
Ejection fraction decreased	-	Resting ejection fraction (EF) 50 - 40%; 10 - 19% drop from baseline	Resting ejection fraction (EF) 39 - 20%; >20% drop from baseline	Resting ejection fraction (EF) <20%	-
Definition: The percentage computed when the amount of blood ejected during a ventricular contraction of the heart is compared to the amount that was present prior to the contraction.					

Common Terminology Criteria for Adverse Events. Version 4.0. Published May 28, 2009 (v4.03: 14 June 2010). US Department of Health and Human Services, National Institutes of Health, National Cancer Institute (http://evs.nci.nih.gov/ftp1/CTCAE/CTCAE-4.03-2010-06-14-QuickReference-5x7.pdf).

"Use of this document is governed by the terms of use on the first page of this document."

Appendix 5
Tumor Assessments (Response Evaluation Criteria in Solid Tumors) Version 1.1

1. Definitions

At baseline, tumor lesions/lymph nodes will be categorized measurable or non-measurable as follows:

Measurable tumor lesions: Must be accurately measured in at least one dimension (longest diameter in the plane of measurement is to be recorded) with a minimum of:

- 10 mm by CT scan (CT scan slice thickness no greater than 5 mm)
- 10 mm caliper measurement by clinical exam (lesions which cannot accurately be measured with calipers should be recorded as non-measurable)
- 20 mm by chest X-ray

Malignant lymph nodes: To be considered pathologically enlarged and measurable, a lymph node must be ≥15 mm in short axis when assessed by CT scan (CT scan slice thickness recommended to be no greater than 5 mm). At baseline and in follow-up, only the short axis will be measured and followed.

Non-measurable lesions: All other lesions including small lesions (longest diameter <10 mm or pathological lymph nodes with P10 to <15 mm short axis) as well as truly non-measurable lesions. Lesions considered truly non-measurable include: leptomeningeal disease, ascites, pleural or pericardial effusion, inflammatory breast disease, lymphangitic involvement of skin or lung, abdominal masses/abdominal organomegaly identified by physical examination that is not measurable by reproducible imaging techniques.

Special considerations regarding lesion measurability

Bone lesions, cystic lesions, and lesions previously treated with local therapy require particular comment.

Bone lesions:

- Bone scan, PET scan, or plain films are not considered adequate imaging techniques to measure bone lesions. However, these techniques can be used to confirm the presence or disappearance of bone lesions.
- Lytic bone lesions or mixed lytic-blastic lesions with identifiable soft tissue components that can be evaluated by cross-sectional imaging techniques such as CT or MRI can be considered as measurable lesions if the soft tissue component meets the definition of measurability described above.
- Blastic bone lesions are non-measurable.

"Use of this document is governed by the terms of use on the first page of this document."

Cystic lesions:

- Lesions that meet the criteria for radiographically defined simple cysts should not be considered as malignant lesions (neither measurable nor non-measurable) since they are, by definition, simple cysts.
- Cystic lesions thought to represent cystic metastases can be considered as measurable lesions if they meet the definition of measurability described above. However, if non-cystic lesions are present in the same patient, these are preferred for selection as target lesions.

Lesions with prior local treatment:

- Tumor lesions situated in a previously irradiated area or in an area subjected to other loco-regional therapy are usually not considered measurable unless there has been demonstrated progression in the lesion. Study protocols should detail the conditions under which such lesions would be considered measurable.

2. Methods of measurements

Measurement of lesions

- All measurements should be recorded in metric notation with use of calipers if clinically assessed. All baseline evaluations should be performed as close as possible to the treatment start and never more than 4 weeks (28 days) before the beginning of the treatment.

Method of assessment

The same method of assessment and the same technique should be used to characterize each identified and reported lesion at baseline and during follow-up. Imaging-based evaluation should always be done rather than clinical examination unless the lesion(s) being followed cannot be imaged but are assessable by clinical examination.

- **Clinical lesions**: Clinical lesions will only be considered measurable when they are superficial and $\geq$10 mm diameter as assessed with use of calipers (e.g., skin nodules). For the case of skin lesions, documentation by color photography, including a ruler to estimate the size of the lesion, is suggested. When lesions can be evaluated by both clinical examination and imaging, imaging evaluation should be undertaken since it is more objective and may also be reviewed at the end of the study.
- **Chest X-ray**: Chest CT is preferred over chest X-ray, particularly when progression is an important endpoint, since CT is more sensitive than X-ray, particularly in identifying new lesions. However, lesions on chest X-ray may be considered measurable if they are clearly defined and surrounded by aerated lung.
- **CT/MRI**: CT is the best currently available and reproducible method to measure lesions selected for response assessment. This guideline has defined measurability of lesions on CT scan on the basis of the assumption that CT slice thickness is

"Use of this document is governed by the terms of use on the first page of this document."

5 mm or less. As is described in “Specification for standard anatomical radiological imaging” (article Eisenhauer 2009, Appendix II), when CT scans have slice thickness greater than 5 mm, the minimum size for a measurable lesion should be twice the slice thickness. MRI is also acceptable in certain situations (e.g., for body scans).

- **Ultrasound**: Ultrasound is not useful in assessment of lesion size and should not be used as a method of measurement. Ultrasound examinations cannot be reproduced in their entirety for independent review at a later date and, because they are operator dependent, it cannot be guaranteed that the same technique and measurements will be taken from one assessment to the next. If new lesions are identified by ultrasound in the course of the study, confirmation by CT or MRI is advised. If there is concern about radiation exposure at CT, MRI may be used instead of CT in selected instances.
- **Endoscopy, laparoscopy**: The utilization of these techniques for objective tumor evaluation is not advised. However, they can be useful to confirm complete pathological response when biopsies are obtained or to determine relapse in trials where recurrence following complete response or surgical resection is an endpoint.
- **Tumor markers**: Tumor markers alone cannot be used to assess objective tumor response. If markers are initially above the upper normal limit, however, they must normalize for a patient to be considered in complete response. Because tumor markers are disease specific, instructions for their measurement should be incorporated into protocols on a disease-specific basis.
- **Cytology/histology**: These techniques can be used to differentiate between PR and CR in rare cases if required by protocol (e.g., residual lesions in tumor types such as germ cell tumors, where known residual benign tumors can remain). When effusions are known to be a potential adverse effect of treatment (e.g., with certain taxane compounds or angiogenesis inhibitors), the cytological confirmation of the neoplastic origin of any effusion that appears or worsens during treatment can be considered if the measurable tumor has met criteria for response or stable disease in order to differentiate between response (or stable disease) and PD.

3. Tumor response evaluation

Assessment of overall tumor burden and measurable disease:

To assess objective response or future progression, it is necessary to estimate the overall tumor burden at baseline and use this as a comparator for subsequent measurements. Only patients with measurable disease at baseline should be included in protocols where objective tumor response is the primary endpoint. Measurable disease is defined by the presence of at least one measurable lesion (as detailed above). In studies where the primary endpoint is tumor progression (either time to progression or proportion with progression at a fixed date), the protocol must specify if entry is restricted to those with measurable disease or whether patients having non-measurable disease only are also eligible.

"Use of this document is governed by the terms of use on the first page of this document."

Baseline documentation of ‘target’ and ‘non-target’ lesions:

When more than one measurable lesion is present at baseline, all lesions up to a maximum of five lesions total (and a maximum of two lesions per organ) representative of all involved organs should be identified as target lesions and will be recorded and measured at baseline (this means in instances where patients have only one or two organ sites involved a maximum of two and four lesions, respectively, will be recorded).

Target lesions should be selected on the basis of their size (lesions with the longest diameter), be representative of all involved organs, but in addition, should be those that lend themselves to reproducible repeated measurements. It may be the case that, on occasion, the largest lesion does not lend itself to reproducible measurement in which circumstance the next largest lesion that can be measured reproducibly should be selected.

Lymph nodes merit special mention since they are normal anatomical structures that may be visible by imaging even if not involved by tumor. Pathological nodes that are defined as measurable and may be identified as target lesions must meet the criterion of a short axis of $\geq$15 mm by CT scan. Only the short axis of these nodes will contribute to the baseline sum. The short axis of the node is the diameter normally used by radiologists to judge if a node is involved by solid tumor. Nodal size is normally reported as two dimensions in the plane in which the image is obtained (for CT scan this is almost always the axial plane; for MRI the plane of acquisition may be axial, sagittal, or coronal). The smaller of these measures is the short axis. For example, an abdominal node that is reported as being 20 mm $\times$30 mm has a short axis of 20 mm and qualifies as a malignant, measurable node. In this example, 20 mm should be recorded as the node measurement. All other pathological nodes (those with short axis $\geq$10 mm but $<$15 mm) should be considered non-target lesions. Nodes that have a short axis $<$10 mm are considered non-pathological and should not be recorded or followed.

A sum of the diameters (longest for non-nodal lesions, short axis for nodal lesions) for all target lesions will be calculated and reported as the baseline sum diameters. If lymph nodes are to be included in the sum, then as noted above, only the short axis is added into the sum. The baseline sum diameters will be used as reference to further characterize any objective tumor regression in the measurable dimension of the disease.

All other lesions (or sites of disease), including pathological lymph nodes, should be identified as non-target lesions and should also be recorded at baseline. Measurements are not required and these lesions should be followed as “present,” “absent,” or in rare cases “unequivocal progression.” In addition, it is possible to record multiple non-target lesions involving the same organ as a single item on the case record form (e.g., “multiple enlarged pelvic lymph nodes” or “multiple liver metastases”).

"Use of this document is governed by the terms of use on the first page of this document."

Response criteria

Evaluation of **target lesions**:

- Complete Response (CR): Disappearance of all target lesions. Any pathological lymph nodes (whether target or non-target) must have reduction in short axis to $<$10 mm.
- Partial Response (PR): At least a 30% decrease in the sum of diameters of target lesions, taking as reference the baseline sum diameters.
- Progressive Disease (PD): At least a 20% increase in the sum of diameters of target lesions, taking as reference the smallest sum on study (this includes the baseline sum if that is the smallest in the study). In addition to the relative increase of 20%, the sum must also demonstrate an absolute increase of at least 5 mm. (Note: the appearance of one or more new lesions is also considered progression.)
- Stable Disease (SD): Neither sufficient shrinkage to qualify for PR nor sufficient increase to qualify for PD, taking as reference the smallest sum diameters while in the study.

Evaluation of **non-target lesions:**

While some non-target lesions may actually be measurable, they need not be measured and instead should be assessed only qualitatively at the timepoints specified in the protocol.

- Complete Response (CR): Disappearance of all non-target lesions and normalization of tumor marker level. All lymph nodes must be non-pathological in size ($<$10 mm short axis).
- Non-CR/Non-PD: Persistence of one or more non-target lesion(s) and/or maintenance of tumor marker level above the normal limits.
- Progressive Disease (PD): Unequivocal progression of existing non-target lesions. (Note: the appearance of one or more new lesions is also considered progression.)

Evaluation of best overall response

The best overall response is the best response recorded from the start of the study treatment until the end of treatment taking into account any requirement for confirmation. On occasion a response may not be documented until after the end of therapy so protocols should be clear if post-treatment assessments are to be considered in determination of best overall response. Protocols must specify how any new therapy introduced before progression will affect best response designation.

The patient's best overall response assignment will depend on the findings of both target and non-target disease and will also take into consideration the appearance of new lesions. Furthermore, depending on the nature of the study and the protocol requirements, it may also require confirmatory measurement. Specifically, in non-randomized trials where response is the primary endpoint, confirmation of PR or CR

"Use of this document is governed by the terms of use on the first page of this document."

is needed to deem either one the “best overall response.” This is described further below.

Target Lesions	Non-target Lesions	New Lesions	Overall Response
CR	CR	No	CR
CR	Non-CR/Non-PD	No	PR
CR	Not evaluated	No	PR
PR	Non-PD or not all evaluated	No	PR
SD	Non-PD or not all evaluated	No	SD
Not all evaluated	Non-PD	No	Not evaluable
PD	Any	Yes or No	PD
Any	PD	Yes or No	PD
Any	Any	Yes	PD

CR = complete response; PD = progressive disease; PR = partial response; SD = stable disease.

Source: Eisenhauer EA, Therasse P, Bogaerts J, et al. New response evaluation criteria in solid tumours: revised RECIST guideline (version 1.1). Eur J Cancer 2009;45:228–47.

"Use of this document is governed by the terms of use on the first page of this document."
