# Supplementary material for: Subcutaneous trastuzumab with pertuzumab and docetaxel in HER2-positive metastatic breast cancer: Final analysis of MetaPHER, a phase IIIb single-arm safety study
Source: Breast Cancer Res Treat. 2021 Mar 21;187(2):467–76. doi: 10.1007/s10549-021-06145-3 (PMC8189949; doi:10.1007/s10549-021-06145-3)
Supplement: Supplementary file 2 — Supplementary file2 (pdf 152 kb) [file 10549_2021_6145_MOESM2_ESM.pdf]

Article type: Original Research Article

Title: Subcutaneous trastuzumab with pertuzumab and docetaxel in HER2-positive metastatic breast cancer: Final analysis of MetaPHER, a phase IIIb single-arm safety study

Authors: Sherko Kuemmel[1,2]*, Carlo A. Tondini[3], Jacinta Abraham[4], Zbigniew Nowecki[5], Bartosz Itrych[6,†], Erika Hitre[7], Bogusława Karaszewska[8], Alejandro Juárez-Ramiro[9], Flavia Morales-Vásquez[10], Jose Manuel Pérez García[11‡], Servando Cardona-Huerta[12], Estefania Monturus[13], Marco Sequi[14,15§], Eleonora Restuccia[13], Mark Benyunes[16] and Miguel Martín[17]

Author affiliations:

[1] Breast Unit, Kliniken Essen-Mitte, Essen, Germany

[2] Clinic for Gynecology with Breast Center, Charité – Universitätsmedizin Berlin, Berlin, Germany

[3] Department of Onco-Hematology, ASST Papa Giovanni XXIII, Bergamo, Italy

[4] Department of Clinical Oncology, Velindre Cancer Centre, Cardiff, UK

[5] Klinika Nowotworów Piersi i Chirurgii Rekonstrukcyjnej, Centrum Onkologii-Instytut, Warsaw, Poland

[6] Department of Oncology, Magodent, Warsaw, Poland

[7] Department of Medical Oncology and Clinical Pharmacology “B”, National Institute of Oncology, Budapest, Hungary

[8] Przychodnia Lekarska KOMED, Konin, Poland

[9] Medical Oncology, CME Consultorio de Medicina Especializada, Mexico City, Mexico

[10] FUCAM, Instituto Nacional de Cancerología de Mexico, Mexico City, Mexico

[11] Medical Oncology Department, Vall d´Hebron Institute of Oncology (VHIO), Hospital Universitari Vall d'Hebron, Barcelona, Spain

[12] Centro de Cáncer de Mama, Hospital Zambrano-Hellion, Tecnológico de Monterrey, Monterrey, Mexico

[13] Global Product Development, F. Hoffmann-La Roche Ltd, Basel, Switzerland

[14] Biostatistics, F. Hoffmann-La Roche Ltd, Basel, Switzerland

[15] Biostatistics, PAREXEL, Milan, Italy

[16] Global Product Development, Genentech, Inc., South San Francisco, CA, USA

[17] Instituto de Investigación Sanitaria Gregorio Marañón, CIBERONC, Departamento de Medicina, Universidad Complutense de Madrid, Madrid, Spain

*** Corresponding author:** Sherko Kuemmel, MD, PhD

Address: Breast Unit, Kliniken Essen-Mitte, Henricistrasse 92, 45136 Essen, Germany

Telephone: +49-201-174-33001

Email: s.kuemmel@kem-med.com

[†] **Current address:** Department of Oncology and Hematology, Central Clinical Hospital MSWiA, Warsaw, Poland (Itrych)

‡ **Current address:** International Breast Cancer Center (IBCC), Quiron Group, Barcelona, Spain (Pérez García)

§ Current address: CROS Academy, Bologna, Italy (Sequi)

Target journal: *Breast Cancer Research and Treatment*

Online Resource 2 – Baseline demographics and disease characteristics, and treatment history

	H SC + P IV + D IV (*N* = 412)
Mean age, years (SD)	55.6 (11.7)
Age group	
<65 years	310 (75.2)
≥65 years	102 (24.8)
Ethnicity	
Hispanic or Latina	69 (16.7)
Not Hispanic or Latina	305 (74)
Not stated/unknown	38 (9.3)
Race	
Asian	2 (0.5)
Black	8 (1.9)
White	347 (84.2)
American Indian or Alaska Native	26 (6.3)
Other	2 (0.5)
Unknown	27 (6.6)
Reproductive status	
Postmenopausal	257 (62.4)
Surgically/naturally sterile	45 (10.9)
With contraceptive protection	110 (26.7)
Without contraceptive protection	0
Breast cancer histology[a]	
Ductal	356 (86.4)
Lobular	28 (6.8)
Medullary	0
Tubular	2 (0.5)
Mucinous	3 (0.7)
Comedo	3 (0.7)
Inflammatory	0
Not otherwise specified	15 (3.6)
Other	9 (2.2)
Disease type at screening	
Visceral	306 (74.3)
Non-visceral	106 (25.7)
Disease status[b]	
Locally recurrent	21 (5.1)
Locally advanced unresectable	37 (9.0)
Metastatic	401 (97.3)
Hormonal status	
ER- and PgR-negative	119 (28.9)
ER- and/or PgR-positive	290 (70.4)
Unknown	3 (0.7)
Prior treatment history	
No prior neoadjuvant or adjuvant only	202 (49.0)
Trastuzumab	131 (31.8)

Taxane	135 (32.8)
Anthracyclines[c]	163 (39.6)
Prior hormonal therapy	139 (33.7)
Radiotherapy	186 (45.1)

[a] *N* = 411 as the histologic subtype of right breast cancer of one patient was confirmed as unknown at the time of data cutoff. Patients could have more than one type of breast cancer histology

[b] Patients could have more than one disease status

[c] Of the 216 (52.4%) patients who received at least one prior systematic therapy (neoadjuvant, adjuvant, metastatic, or other), 162 (75.3%) received an anthracycline regimen

D IV intravenous docetaxel, *ER* estrogen receptor, *H SC* subcutaneous trastuzumab, *PgR* progesterone receptor, *P IV* intravenous pertuzumab, *SD* standard deviation

Data are number of patients (%)
