# Supplementary material for: Subcutaneous trastuzumab with pertuzumab and docetaxel in HER2-positive metastatic breast cancer: Final analysis of MetaPHER, a phase IIIb single-arm safety study
Source: Breast Cancer Res Treat. 2021 Mar 21;187(2):467–76. doi: 10.1007/s10549-021-06145-3 (PMC8189949; doi:10.1007/s10549-021-06145-3)
Supplement: Supplementary file 5 — Supplementary file5 (pdf 139 kb) [file 10549_2021_6145_MOESM5_ESM.pdf]

Article type: Original Research Article

Title: Subcutaneous trastuzumab with pertuzumab and docetaxel in HER2-positive metastatic breast cancer: Final analysis of MetaPHER, a phase IIIb single-arm safety study

Authors: Sherko Kuemmel[1,2]*, Carlo A. Tondini[3], Jacinta Abraham[4], Zbigniew Nowecki[5], Bartosz Itrych[6,†], Erika Hitre[7], Bogusława Karaszewska[8], Alejandro Juárez-Ramiro[9], Flavia Morales-Vásquez[10], Jose Manuel Pérez García[11‡], Servando Cardona-Huerta[12], Estefania Monturus[13], Marco Sequi[14,15§], Eleonora Restuccia[13], Mark Benyunes[16] and Miguel Martín[17]

Author affiliations:

[1] Breast Unit, Kliniken Essen-Mitte, Essen, Germany

[2] Clinic for Gynecology with Breast Center, Charité – Universitätsmedizin Berlin, Berlin, Germany

[3] Department of Onco-Hematology, ASST Papa Giovanni XXIII, Bergamo, Italy

[4] Department of Clinical Oncology, Velindre Cancer Centre, Cardiff, UK

[5] Klinika Nowotworów Piersi i Chirurgii Rekonstrukcyjnej, Centrum Onkologii-Instytut, Warsaw, Poland

[6] Department of Oncology, Magodent, Warsaw, Poland

[7] Department of Medical Oncology and Clinical Pharmacology “B”, National Institute of Oncology, Budapest, Hungary

[8] Przychodnia Lekarska KOMED, Konin, Poland

[9] Medical Oncology, CME Consultorio de Medicina Especializada, Mexico City, Mexico

[10] FUCAM, Instituto Nacional de Cancerología de Mexico, Mexico City, Mexico

[11] Medical Oncology Department, Vall d´Hebron Institute of Oncology (VHIO), Hospital Universitari Vall d'Hebron, Barcelona, Spain

[12] Centro de Cáncer de Mama, Hospital Zambrano-Hellion, Tecnológico de Monterrey, Monterrey, Mexico

[13] Global Product Development, F. Hoffmann-La Roche Ltd, Basel, Switzerland

[14] Biostatistics, F. Hoffmann-La Roche Ltd, Basel, Switzerland

[15] Biostatistics, PAREXEL, Milan, Italy

[16] Global Product Development, Genentech, Inc., South San Francisco, CA, USA

[17] Instituto de Investigación Sanitaria Gregorio Marañón, CIBERONC, Departamento de Medicina, Universidad Complutense de Madrid, Madrid, Spain

*** Corresponding author:** Sherko Kuemmel, MD, PhD

Address: Breast Unit, Kliniken Essen-Mitte, Henricistrasse 92, 45136 Essen, Germany

Telephone: +49-201-174-33001

Email: s.kuemmel@kem-med.com

[†] **Current address:** Department of Oncology and Hematology, Central Clinical Hospital MSWiA, Warsaw, Poland (Itrych)

‡ **Current address:** International Breast Cancer Center (IBCC), Quiron Group, Barcelona, Spain (Pérez García)

§ Current address: CROS Academy, Bologna, Italy (Sequi)

Target journal: *Breast Cancer Research and Treatment*

Online Resource 5 – Objective response rate

	H SC + P IV + D IV<br>(*n* = 336)
ORR, no. (%) [95% CI]	254 (75.6)<br>[70.64–80.09]
CR	42 (12.5)
PR	212 (63.1)
Non-responders, no. (%)	27 (8.0)
PD	17 (5.1)
Not evaluable	1 (0.3)
Missing	9 (2.7)
Stable disease, no. (%)	55 (16.4)
CBR	309 (92.0)

CBR clinical benefit rate, *CI* confidence interval, *CR* complete response, *D IV* intravenous docetaxel, *H SC* subcutaneous trastuzumab, *ORR* objective response rate, *PD* progressive disease, *P IV* intravenous pertuzumab, *PR* partial response

Data are number of patients (%) [95% CI]
